# Autocrine SFRP2 (secreted frizzled related protein 2) enhances lung myofibroblast fibrogenic activity by suppressing PINK1-mediated mitophagy initiation

**DOI:** 10.1080/15548627.2026.2642341

**Published:** 2026-03-15

**Authors:** Yingying Lin, Tianxiang Lei, Yifan Jia, Meiling Yao, Xiaofeng Wang, Shaojie Huang, Zhongxing Wang, Xiaofan Lai

**Affiliations:** a Department of Anesthesiology, The First Affiliated Hospital, Sun Yat-sen University, Guangzhou, Guangdong Province, China; b Department of Thyroid and Hernia Surgery, Guangdong Provincial People’s Hospital (Guangdong Academy of Medical Sciences), Southern Medical University, Guangzhou, Guangdong Province, China; c Department of Neurology, The Fifth Affiliated Hospital, Sun Yat-sen University, Zhuhai, Guangdong Province, China; d Center for Precise Medicine, The First Affiliated Hospital, Sun Yat-sen University, Guangzhou, Guangdong Province, China

**Keywords:** Extracellular matrix, idiopathic pulmonary fibrosis, mitochondrial reactive oxygen species, myofibroblast fibrogenic activity, PINK1-mediated mitophagy, WNT-Ca^2+^ signaling

## Abstract

Idiopathic pulmonary fibrosis (IPF) is a fatal interstitial lung disease driven by persistent activation of pulmonary myofibroblasts, but the regulatory mechanisms sustaining this pathological state remain incompletely understood. Using single-cell RNA sequencing (scRNA-seq), we identified SFRP2 (secreted frizzled related protein 2) as a critical mediator of profibrotic myofibroblasts in IPF lungs. Functional studies revealed that SFRP2 acted in an autocrine manner to promote myofibroblast activation and extracellular matrix (ECM) production. Mechanistically, SFRP2 activated FZD5-mediated non-canonical WNT-Ca^2+^ signaling, leading to PPP3/calcineurin-dependent translocation of PINK1 from the outer to the inner mitochondrial membrane (IMM), where it was degraded, thereby inhibiting PINK1-mediated mitophagy. Furthermore, therapeutic intervention with AAV6-sh*Sfrp2*, SFRP2-neutralizing antibody, or the autophagy inducer rapamycin significantly ameliorated lung fibrosis in bleomycin (BLM)-induced mouse models. Our results define a novel autocrine SFRP2-mitophagy regulatory axis that perpetuates myofibroblast activation and represents a promising therapeutic target for pulmonary fibrosis.

**Abbreviations**: AAV: adeno-associated virus; BLM: bleomycin; CQ: chloroquine; ECM: extracellular matrix; FZD5: frizzled class receptor 5; H&E: hematoxylin and eosin; IHC: immunohistochemical; IMM: inner mitochondrial membrane; IPF: idiopathic pulmonary fibrosis; Micro-CT: micro-computed tomography; mtROS: mitochondrial reactive oxygen species; PMLFs: primary mouse lung fibroblasts; qPCR: quantitative real-time PCR; scRNA-seq: single-cell RNA sequencing; SFRP2: secreted frizzled related protein 2; TEM: transmission electron microscopy; ∆Ψm: mitochondrial membrane potential.

## Introduction

Pulmonary fibrosis, characterized by excessive accumulation of extracellular matrix (ECM), represents a pathological consequence of impaired lung tissue repair and remodeling [1]. This disruption of normal lung architecture finally leads to respiratory dysfunction and failure [2]. Among the various forms of pulmonary fibrosis, idiopathic pulmonary fibrosis (IPF) is the most common and severe. Recognized as an irreversible and progressive interstitial lung disease of unknown etiology, IPF affects over 5 million individuals globally, primarily those aged 50 and above [2]. With a median survival of only 3–5 years, IPF has a prognosis worse than lung cancer [1]. Currently, no curative medical therapy exists for this condition, highlighting the urgent need to identify novel molecular targets and develop innovative therapeutic strategies to address this clinical challenge.

Myofibroblasts are critical effector cells orchestrating pathological ECM deposition in IPF, where they persist in an aberrantly activated state through sustained TGFB/TGF-β signaling, mitochondrial dysfunction, and epigenetic modifications [3,4]. Unlike their transient counterparts in wound healing, IPF myofibroblasts exhibit 3–5-fold higher ECM production, maintain pro-survival pathways and undergo metabolic reprogramming to aerobic glycolysis, collectively contributing to the destruction of lung tissue architecture and the progression of pulmonary fibrosis [5,6]. Thus, targeting these hyperactive myofibroblasts in pulmonary fibrosis could offer a promising approach to resolve fibrosis and mitigate lung damage.

SFRP2 (secreted frizzled related protein 2), a secreted glycoprotein, was recognized for its pivotal role in promoting cell activity and the production of ECM components [7,8]. Elevated levels of SFRP2 have been observed in aged fibroblasts, which correlated with increased oxidative stress responses and enhanced invasiveness in melanoma cells [9]. Conversely, SFRP2 inhibition reduced excessive ECM deposition in cardiomyocytes, leading to improved cardiac function [10]. Recently, SFRP2 was identified as a marker of a progenitor fibroblast population that gives rise to myofibroblasts in systemic sclerosis [11]. Despite these insights, the precise mechanisms by which SFRP2 influences pulmonary fibrosis, particularly in the regulation of myofibroblast fibrogenic activity, remain poorly defined.

Myofibroblasts in fibrotic lung tissue exhibit impaired mitophagy, resulting in mitochondrial reactive oxygen species (mtROS) accumulation and subsequent cellular stress [1,12]. These alterations drive excessive ECM production and accelerate pulmonary fibrosis progression [1,13]. Notably, impaired PINK1 (PTEN induced kinase 1)-mediated mitophagy promoted TGFB/TGF-β-induced myofibroblast activation and ECM deposition [13], with PINK1 deficiency exacerbating lung fibrosis in mouse models [14]. These findings underscore the importance of PINK1-mediated mitophagy in regulating pulmonary fibrosis. Recent studies have indicated that SFRP2 modulated intracellular oxidative stress by regulating mitochondrial dynamics and quality control, thereby influencing cellular phenotype transformation and disease progression [15,16]. Specifically, SFRP2 has been shown to interact with FZD5 (frizzled class receptor 5) to activate the WNT-Ca^2+^ signaling pathway and upregulate intracellular calcium levels within pulmonary fibrosis microenvironment [17]. The increase in calcium has been demonstrated to inhibit PINK1-mediated mitophagy [18]. Whether SFRP2 is closely related to impaired PINK1-mediated mitophagy via the WNT-Ca^2+^ signaling pathway in lung myofibroblasts requires further exploration.

In this study, we revealed that myofibroblasts derived from IPF patients exhibited significantly enhanced capabilities for ECM synthesis and remodeling compared to those from healthy donors through single-cell RNA sequencing (scRNA-seq) analysis. We observed that SFRP2 was specifically highly expressed in myofibroblasts derived from fibrotic lung tissue and promoted myofibroblast activation by inhibiting PINK1-mediated mitophagy initiation. Moreover, SFRP2 promoted the translocation of PINK1 into inner mitochondrial membrane (IMM), thereby inhibiting mitophagy initiation via the FZD5-mediated WNT-Ca^2+^ signaling pathway. Targeting the autocrine SFRP2-mitophagy axis may offer a novel therapeutic strategy for this disease.

## Results

### SFRP2 was specifically highly expressed in myofibroblasts derived from fibrotic lung tissue

**Figure 1.**
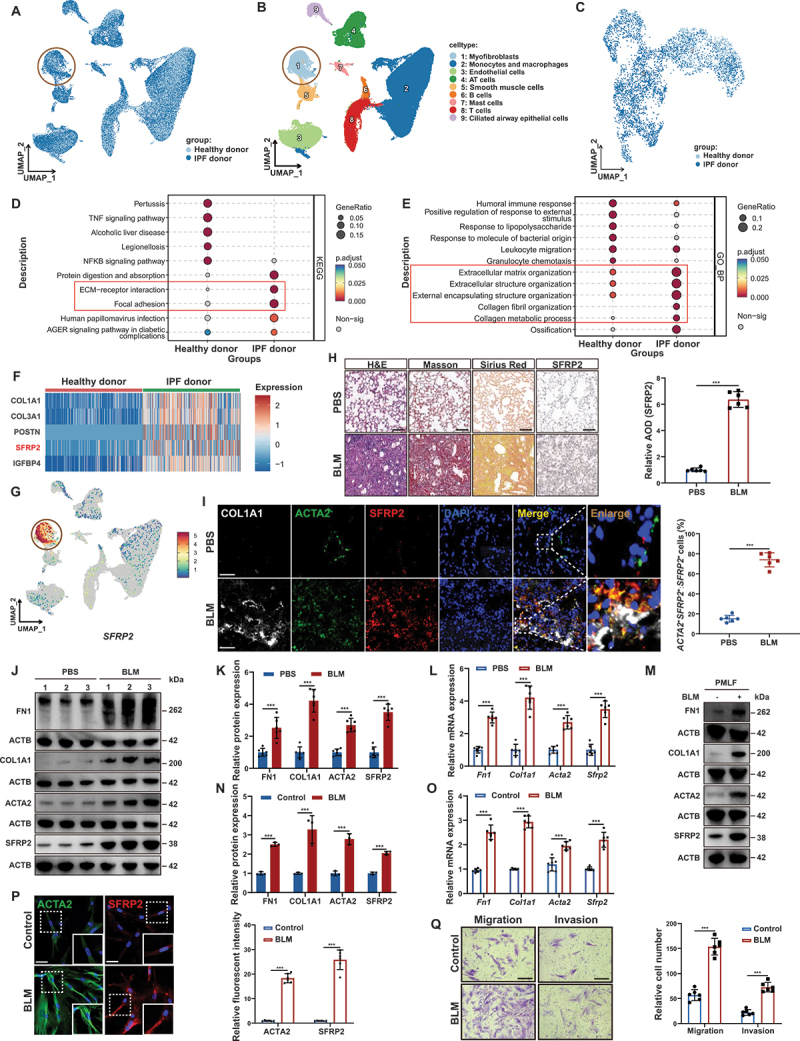
SFRP2 was specifically highly expressed in myofibroblasts derived from fibrotic lung tissue. (**A**) UMAP visualization of all lung cells from IPF patients and healthy donors, integrating data from GSE132771 and GSE128033 datasets. (**B**) UMAP representation of cell clusters identified in the scRNA-seq data (integrating GSE132771 and GSE128033). (**C**) UMAP plots highlighting the myofibroblast subset from the lungs of IPF patients and healthy donors using the scRNA-seq data (integrating GSE132771 and GSE128033). (**D**) KEGG enrichment analysis of genes extracted from myofibroblasts isolated from the lungs of IPF patients and healthy donors using the scRNA-seq data (integrating GSE132771 and GSE128033). (**E**) GO enrichment analysis of genes extracted from myofibroblasts isolated from the lungs of IPF patients and healthy donors using the scRNA-seq data (integrating GSE132771 and GSE128033). (**F**) Heatmap of the top 5 differentially expressed genes (DEGs) between myofibroblasts derived from the lungs of IPF patients and healthy donors using the scRNA-seq data (integrating GSE132771 and GSE128033). (**G**) UMAPs depicting SFRP2 expression in the lungs of IPF patients and healthy donors using the scRNA-seq data (integrating GSE132771 and GSE128033). (**H**) Representative images of H&E staining, Masson’s trichrome staining, Sirius red staining, and IHC staining for SFRP2 in lung sections from experimental mouse models treated with BLM or PBS. Scale bars: 100 µm. Quantitative analysis of the average optical density (AOD) of SFRP2 in IHC images (*n* = 6 biological repeats per group). (**I**) Representative IF images of COL1A1 (white), SFRP2 (red) and ACTA2 (green) in lung sections from experimental mice induced by BLM or PBS. Scale bars: 50 µm. Semiquantitative scoring of the percentage of *ACTA2*^+^*SFRP2*^+^ cells relative to *SFRP2*^+^ cells (*n* = 6 biological repeats per group). (**J**) Western blotting analysis of FN1, COL1A1, ACTA2 and SFRP2 protein levels in the lungs of experimental mice treated with BLM or PBS. (**K**) Quantitative analysis of FN1, COL1A1, ACTA2, and SFRP2 protein levels normalized to ACTB from (**K**) (*n* = 6 biological repeats per group). (**L**) qPCR analysis of *Fn1*, *Col1a1*, *Acta2* and *Sfrp2* mRNA levels in the lungs of experimental mice treated with BLM or PBS (*n* = 6 biological repeats per group). (**M**) Western blotting analysis of FN1, COL1A1, ACTA2, and SFRP2 protein levels in PMLFs obtained from experimental mice treated with BLM or PBS. (**N**) Quantitative analysis of FN1, COL1A1, ACTA2, and SFRP2 protein levels normalized to ACTB from (**M**) (*n* = 3 biological repeats per group). (**O**) qPCR analysis of *Fn1*, *Col1a1*, *Acta2*, and *Sfrp2* mRNA levels in PMLFs obtained from experimental mice treated with BLM or PBS (*n* = 6 biological repeats per group). (**P**) IF staining of ACTA2 and SFRP2 in PMLFs obtained from experimental mice treated with BLM or PBS. Scale bars: 50 µm. Quantitative analysis of relative fluorescent intensity (*n* = 6 biological repeats per group). (**Q**) Representative transwell images showing the migratory and invasive capabilities of PMLFs obtained from experimental mice treated with BLM or PBS. Scale bars: 100 µm. Quantitative analysis of migratory and invasive capabilities (*n* = 6 biological repeats per group). Data are presented as mean ± SD; ***p < 0.001.

In IPF, myofibroblasts represent pivotal effector cells driving aberrant ECM deposition and persistent tissue remodeling within fibrotic lesions [3,4]. To further characterize these cells, we reanalyzed published scRNA-seq datasets (GSE132771 and GSE128033) from previous studies [19,20] and identified a distinct myofibroblast population defined by specific markers (Figure 1(A-C) and Figure S1A). Gene ontology (GO) and Kyoto Encyclopedia of Genes and Genomes (KEGG) enrichment analyses revealed significant enrichment of ECM synthesis and remodeling pathways in IPF-derived versus healthy donor myofibroblasts (Figure 1(D,E)). Consistently, IPF myofibroblasts showed markedly elevated expression of canonical fibrotic markers COL1A1 (collagen, type 1, alpha 1), FN1 (fibronectin 1) and ACTA2/α-SMA (Figure S1B-E). Through differential expression analysis, we identified SFRP2 as one of the top five upregulated genes in IPF-derived myofibroblasts (Figure 1(F), Data S1), with specific enrichment in this pathological cell population (Figure 1(G), S1E). Pseudotime trajectory analysis revealed a continuous differentiation path from normal myofibroblasts to activated IPF myofibroblasts, showing a progressive increase in SFRP2 expression along the diffusion pseudotime (DPT) axis (Figure S1F, G). This coordinated upregulation of SFRP2 with key profibrotic genes (COL1A1, FN1, and ACTA2) suggested SFRP2 might drive the transition to the pathological myofibroblast state. Bulk RNA-seq of lung tissues confirmed SFRP2 upregulation in IPF lungs, showing positive correlation with ACTA2 and COL1A1 expression (Figure S1H, I). Collectively, these results indicate SFRP2 as a key mediator of myofibroblast activation in pulmonary fibrosis.

To investigate SFRP2’s functional role in pulmonary fibrosis, we employed two established mouse models of experimental fibrosis induced by bleomycin (BLM) or adenoviral TGFB1/TGF-β1 (Ad*Tgfb1*) (Figure S1J And Figure S2A). Both models exhibited key disease features, including reduced body weight, diminished survival (Figure S1K, L And Figure S2B, C), and significant COL1A1 deposition accompanied by elevated SFRP2 expression in fibrotic lungs (Figure 1(H-L), Figure S1M, N, and Figure S2D-I). SFRP2 levels showed positive correlation with ACTA2 and COL1A1 accumulation in fibrotic lungs (Figure S1O, P and Figure S2L, M). Immunofluorescent (IF) assays revealed that SFRP2 was predominantly localized in *ACTA2*^+^ myofibroblasts within fibrotic lung tissues from both experimental mouse models and IPF patients (Figure 1(I), Figure S1Q And Figure S2J, K). Moreover, primary myofibroblasts isolated from BLM-injured lungs showed upregulated expression of FN1, COL1A1, ACTA2, and SFRP2 (Figure 1(J-P)), along with enhanced migratory and invasive capacities (Figure 1(Q)). Similar results were obtained in TGFB/TGF-β-induced myofibroblasts (Figure S2N-R). Collectively, these results indicated that SFRP2 was predominantly upregulated in myofibroblasts within fibrotic lungs, indicating its role in driving fibrosis.

### SFRP2 enhanced myofibroblast fibrogenic activity through an autocrine mechanism

**Figure 2.**
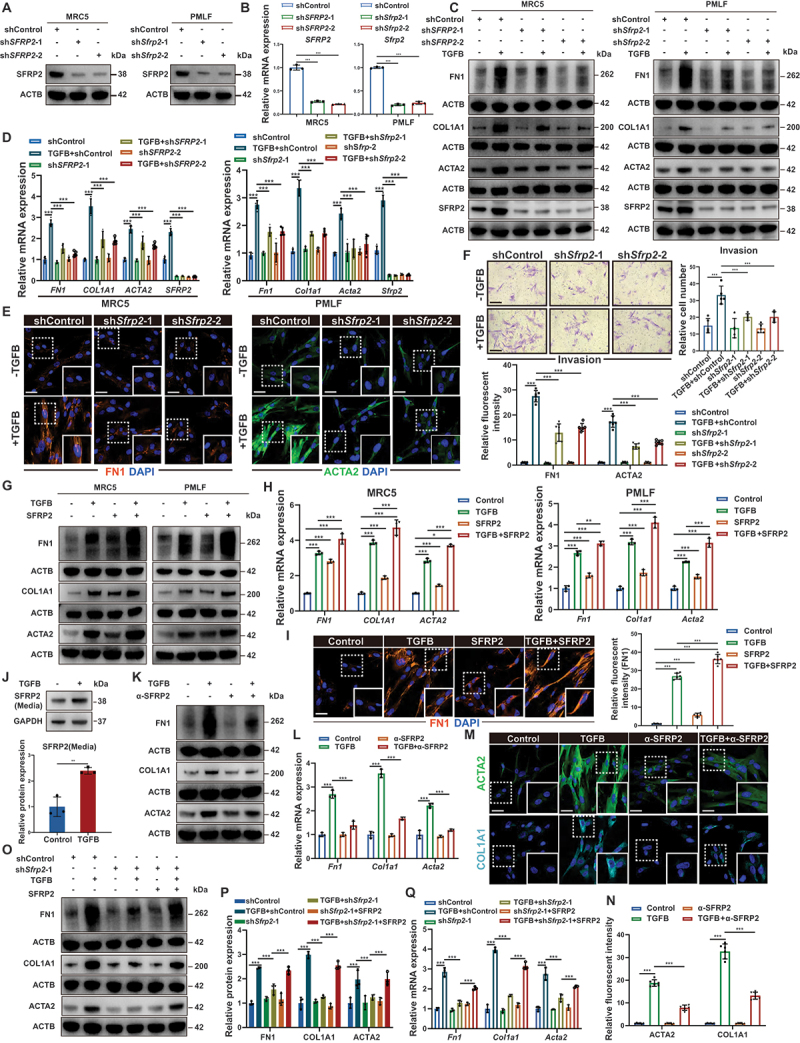
SFRP2 promoted myofibroblast fibrogenic activity through an autocrine mechanism. (**A**) Western blotting analysis of SFRP2 expression in following SFRP2 knockdown (*n* = 3 biological repeats per group). (**B**) qPCR analysis of SFRP2 expression in MRC5 cells and PMLFs following SFRP2 knockdown (*n* = 3 biological repeats per group). (**C**) Western blotting analysis of FN1, COL1A1, ACTA2 and SFRP2 expression in MRC5 cells and PMLFs activated by TGFB/TGF-β and subsequently subjected to SFRP2 knockdown. (**D**) qPCR analysis of *FN1, COL1A1, ACTA2* and *SFRP2* expression in MRC5 cells and PMLFs activated by TGFB/TGF-β and subsequently subjected to SFRP2 knockdown (*n* = 5 biological repeats per group). (**E**) IF staining of FN1 and ACTA2 in PMLFs activated by TGFB/TGF-β and subsequently subjected to SFRP2 knockdown. Scale bars: 50 µm. Quantitative analysis of relative fluorescent intensity in IF images (*n* = 6 biological repeats per group). (**F**) Representative transwell images showing the invasive capabilities of PMLFs activated by TGFB/TGF-β and subsequently subjected to SFRP2 knockdown. Scale bars: 100 µm. Quantitative analysis of invasive capabilities of PMLFs in transwell images (*n* = 5 biological repeats per group). (**G**) Western blotting analysis of FN1, COL1A1 and ACTA2 in PMLFs treated with TGFB/TGF-β and SFRP2. (**H**) qPCR analysis of *FN1*, *COL1A1*, *ACTA2* and *SFRP2* mRNA levels in MRC5 cells and PMLFs treated with TGFB/TGF-β and SFRP2 (*n* = 3 biological repeats per group). (**I**) IF staining of FN1 treated with TGFB/TGF-β and SFRP2. Scale bars: 50 µm. Quantitative analysis of relative fluorescent intensity in IF images (*n* = 6 biological repeats per group). (**J**) Western blotting analysis of secreted SFRP2 levels in the 24-h conditioned media from PMLFs activated by TGFB/TGF-β (*n* = 3 biological repeats per group). (**K**) Western blotting analysis of FN1, COL1A1, and ACTA2 expression in PMLFs activated by TGFB/TGF-β, with or without the addition of α-SFRP2. (**L**) qPCR analysis of *Fn1*, *Col1a1*, and *Acta2* mRNA levels in PMLFs activated by TGFB/TGF-β, with or without the addition of α-SFRP2 (*n* = 3 biological repeats per group). (**M**) IF staining of ACTA2 and COL1A1 in PMLFs activated by TGFB/TGF-β, with or without the addition of α-SFRP2. Scale bars: 50 µm. (**N**) Quantitative analysis of ACTA2 and COL1A1 from (**M**). (**O**) Western blotting analysis of FN1, COL1A1, and ACTA2 expression in PMLFs activated by TGFB/TGF-β and subsequently subjected to SFRP2 knockdown, with or without treatment with SFRP2. (**P**) Quantitative analysis of FN1, COL1A1, and ACTA2 protein level normalized to ACTB from (**O**) (*n* = 3 biological repeats per group). (**Q**) qPCR analysis of *Fn1*, *Col1a1*, and *Acta2* mRNA levels in PMLFs activated by TGFB/TGF-β and subsequently subjected to SFRP2 knockdown, with or without treatment with SFRP2. Data are presented as mean ± SD; *p < 0.05; **p < 0.01; ***p < 0.001.

While SFRP2 has been implicated in enhancing cellular activity and ECM production in other contexts [9,10,21,22], its specific function in myofibroblasts remained undefined. Our analysis of bulk RNA-seq data revealed that the differentially expressed genes in the high SFRP2 group were significantly enriched in ECM synthesis and remodeling pathways in lung tissues (Figure S3A). To functionally explore this association, we knocked down SFRP2 using two independent shRNAs in both MRC5 cells and primary mouse lung fibroblasts (PMLFs), confirming efficient reduction at the mRNA and protein levels (Figure 2(A,B) and Figure S3B). SFRP2 knockdown significantly suppressed the expression of key fibrotic markers (FN1, COL1A1 and ACTA2) and impaired the migratory and invasive capacities of TGFB/TGF-β-induced myofibroblasts (Figure 2(C-F), S3C-F). These findings were corroborated in primary myofibroblasts isolated from BLM-injured mice, where SFRP2 knockdown similarly attenuated fibrogenic and pro-invasive phenotypes (Figure S3G-I).

Given SFRP2’s role as a secreted modulator of cellular signaling [23,24], we investigated its mode of action. Exogenous recombinant SFRP2 significantly enhanced myofibroblast fibrogenic activity and invasive capacity (Figure 2(G-I) And Figure S3J, K). Additionally, we observed increased SFRP2 secretion in conditioned medium from TGFB/TGF-β-induced myofibroblasts (Figure 2(J)), suggesting an autocrine signaling loop. Functional blockade of this autocrine signaling loop using SFRP2 neutralizing antibody significantly reduced fibrotic marker expression (FN1, COL1A1 and ACTA2) (Figure 2(K-N), and Figure S3L). Conversely, the reintroduction of recombinant SFRP2 into SFRP2-knockdown myofibroblasts markedly restored the expression of these fibrotic markers (Figure 2(O-Q)). Collectively, these findings demonstrated that autocrine SFRP2 signaling was both necessary and sufficient for sustaining myofibroblast activation in pulmonary fibrosis.

### SFRP2 inhibited PINK1-mediated mitophagy in myofibroblasts

**Figure 3.**
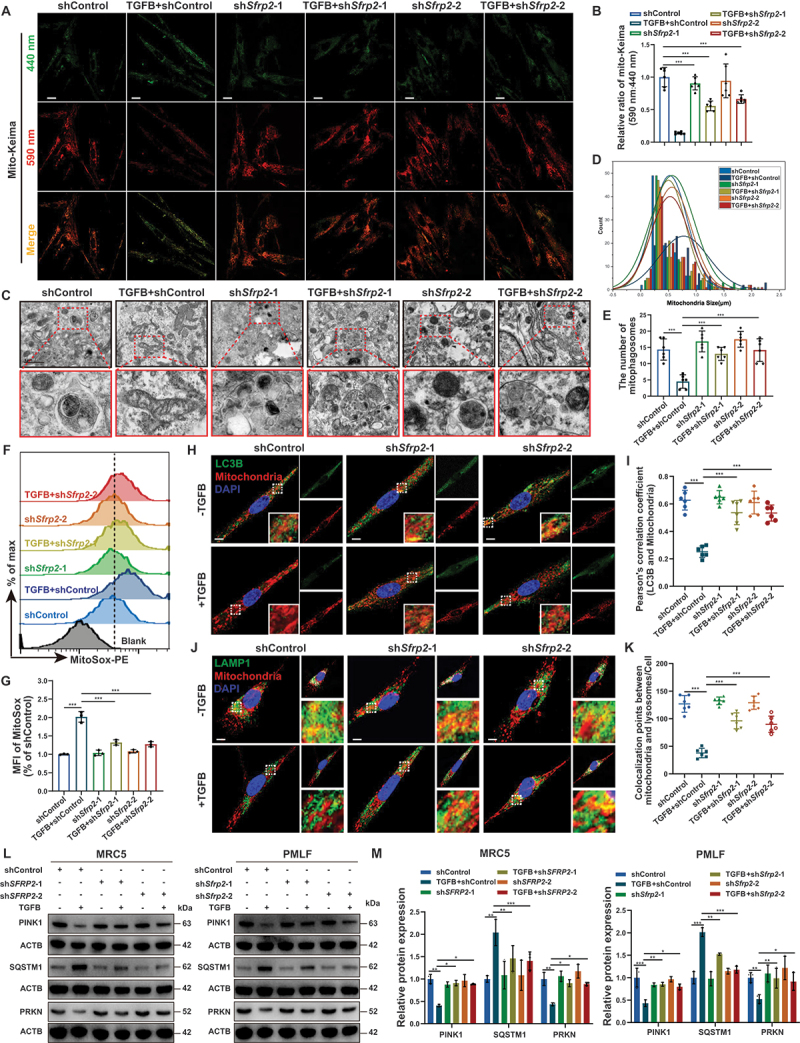
SFRP2 inhibited PINK1-mediated mitophagy in myofibroblasts. (**A**) Representative IF images of PMLFs transfected with mito-Keima lentiviral vectors, following activation by TGFB/TGF-β and subsequent SFRP2 knockdown. Fluorescence was detected at 440 nm (neutral pH) or 590 nm (acidic pH) to monitor mitophagic activity. Scale bars: 20 µm. (**B**) Quantitative analysis of the mitophagic flux index, presented as the ratio of 590 nm:440 nm fluorescence signals from (**A**) (*n* = 6 biological repeats per group). (**C**) Representative TEM images of PMLFs following activation by TGFB/TGF-β and subsequent SFRP2 knockdown. Scale bars: 1 μm. (**D**) Quantitative morphometric analyses using TEM to assess mitochondrial size from (**C**) (*n* = 100 mitochondria per group). (**E**) Quantitative analysis of the count of mitophagosomes from (**C**) (*n* = 6 biological repeats per group). (**F**) Flow cytometry analysis of mtROS levels in PMLFs activated by TGFB/TGF-β and subsequent SFRP2 knockdown. MtROS were detected using MitoSOX, with fluorescence measured in the PE channel. (**G**) Quantitative analysis of mean fluorescence intensity (MFI) of MitoSOX to assess mtROS levels from (**F**) (*n* = 3 biological repeats per group). (**H**) Representative IF images showing colocalization of LC3B (green) and mitochondria (MitoTracker, red) in PMLFs activated by TGFB/TGF-β and subsequent to SFRP2 knockdown following treatment with CQ, 20 µM, 24 h. Scale bars: 10 µm. (**I**) Pearson correlation coefficient analysis to measure the colocalization between LC3B and mitochondria from (**H**) (*n* = 6 biological repeats per group). (**J**) Representative IF images showing colocalization of LAMP1 (green) and mitochondria (MitoTracker, red) in PMLFs activated by TGFB/TGF-β and subsequent SFRP2 knockdown following treatment with CQ (20 µM, 24 h). Sites of colocalization are indicated by white points. Scale bars: 10 µm. (**K**) Quantitative analysis of the count of colocalization points between LAMP1 and mitochondria from (**J**). (*n* = 6 biological repeats per group). (**L**) Western blotting analysis of PINK1, SQSTM1/p62, and PRKN expression in MRC5 cells and PMLFs activated by TGFB/TGF-β and subsequent SFRP2 knockdown. (**M**) Quantitative analysis of PINK1, SQSTM1/p62, and PRKN protein levels normalized to ACTB (*n* = 3 biological repeats per group). Data are presented as mean ± SD; *: *p* < 0.05; **: *p* < 0.01; ***: *p* < 0.001.

Myofibroblasts in fibrotic lungs exhibit impaired mitophagy [1,4]. Given SFRP2’s established role in mitochondrial regulation [12,15,16], we investigated its potential involvement in mitophagic regulation. Using the pH-sensitive biosensor mito-Keima [25], we found that TGFB/TGF-β-induced myofibroblasts displayed reduced 590 nm excitation signal, indicating suppressed mitophagic flux, which was significantly rescued by SFRP2 knockdown (Figure 3(A,B)). Transmission electron microscopy (TEM) confirmed these findings, revealing enlarged, damaged mitochondria and impaired mitophagosome formation in TGFB/TGF-β-induced myofibroblasts, both of which were reversed upon SFRP2 knockdown (Figure 3(C-E) And Figure S4A, B). As TGFB/TGF-β-induced myofibroblasts overproduce mtROS due to mitophagy impairment [12]. Consistent with mitophagy impairment, TGFB/TGF-β-induced mtROS overproduction was ameliorated by SFRP2 knockdown (Figure 3(F,G)).

**Figure 4.**
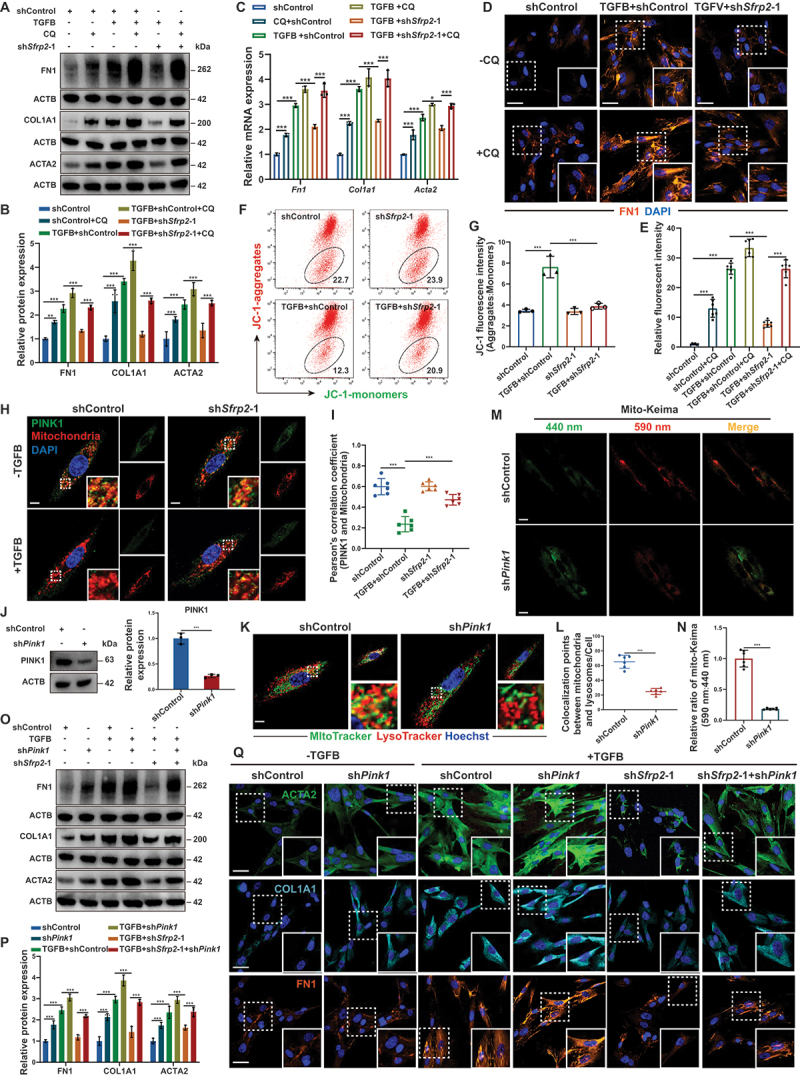
SFRP2 promoted myofibroblast fibrogenic activity by inhibiting PINK1-mediated mitophagy initiation. (**A**) Western blotting analysis of FN1, COL1A1, and ACTA2 protein levels in PMLFs activated by TGFB/TGF-β, following SFRP2 knockdown and treatment with CQ (20 µM, 24 h). (**B**) Quantitative analysis of FN1, COL1A1, and ACTA2 protein levels normalized to ACTB (*n* = 3 biological repeats per group). (**C**) qPCR analysis of *Fn1*, *Col1a1* and *Acta2* mRNA levels in PMLFs activated by TGFB/TGF-β, following SFRP2 knockdown and treatment with CQ (20 µM, 24 h) (*n* = 3 biological repeats per group). (**D**) IF staining of FN1 in PMLFs activated by TGFB/TGF-β, following SFRP2 knockdown and treatment with CQ (20 µM, 24 h). (**E**) Quantitative analysis of relative fluorescent intensity from (**D**) (*n* = 6 biological repeats per group). (**F**) Flow cytometry analysis of mitochondrial membrane potential (ΔΨm) probed with JC-1 in PMLFs treated with TGFB/TGF-β and subjected to SFRP2 knockdown. (**G**) Quantitative analysis of the ratio of JC-1 aggregates (indicative of high ΔΨm, PE channel) to JC-1 monomers (indicative of low ΔΨm, FITC channel) from (**F**) (*n* = 3 biological repeats per group). (**H**) IF staining depicting the colocalization of PINK1 (green) and mitochondria (MitoTracker, red) in PMLFs activated by TGFB/TGF-β and subsequently subjected to SFRP2 knockdown. Scale bars: 10 µm. (**I**) Pearson correlation coefficient analysis to measure the colocalization between PINK1 and mitochondria from (**H**) (*n* = 6 biological repeats per group). (**J**) Western blotting analysis of PINK1 expression in PMLFs following PINK1 knockdown. Quantitative analysis of PINK1 protein levels normalized to ACTB from (**J**) (*n* = 3 biological repeats per group). (**K**) IF staining depicting the colocalization of mitochondria (MitoTracker, green) and lysosome (LysoTracker, red) in PMLFs following PINK1 knockdown. Sites of colocalization are indicated by white points. Scale bars: 10 µm. (**L**) Quantitative analysis of colocalization between lysosome and mitochondria from (**K**). (*n* = 6 biological repeats per group). (**M**) Representative IF images of PMLFs transfected with mito-Keima lentiviral vectors following PINK1 knockdown. Fluorescence was detected at 440 nm (neutral pH) or 590 nm (acidic pH) to monitor mitophagic activity. Scale bars: 20 µm. (**N**) Quantitative analysis of the mitophagic flux index, presented as the ratio of 590 nm:440 nm fluorescence signals from (**M**) (*n* = 6 biological repeats per group). (**O**) Western blotting analysis of FN1, COL1A1, and ACTA2 expression in PMLFs activated by TGFB/TGF-β and subsequently subjected to SFRP2 and PINK1 knockdown. (**P**) Quantitative analysis of FN1, COL1A1, and ACTA2 protein levels normalized to ACTB (*n* = 3 biological repeats per group). (**Q**) IF staining of FN1, COL1A1, and ACTA2 in PMLFs activated by TGFB/TGF-β and subsequently subjected to SFRP2 and PINK1 knockdown. Scale bars: 50 µm. Data are presented as mean±SD; *: *p* < 0.05; **: *p* < 0.01; ***: *p* < 0.001.

MAP1LC3/LC3 (microtubule associated protein 1 light chain 3) is critical for autophagy activation and autophagosome membrane formation [26]. SFRP2 knockdown enhanced LC3B expression and promoted colocalization of mitochondria with both LC3B and lysosomal markers, even under lysosomal inhibitor, chloroquine (CQ) treatment (Figure 3(H-K), S4F, G). This was accompanied by reduced SQSTM1/p62 (sequestosome 1) accumulation (Figure 3(L,M)), indicating enhanced autophagic flux. PINK1-mediated mitophagy is a critical pathway for the selective degradation of mitochondria [27,28]. Depolarized mitochondria stabilize PINK1 on the outer mitochondrial membrane, orchestrating the sequential recruitment of PRKN (parkin RBR E3 ubiquitin protein ligase), LC3, and SQSTM1/p62 to initiate mitophagy [27,29]. SFRP2 knockdown upregulated PINK1 and PRKN expression, increased their mitochondrial localization, and facilitated PRKN translocation to mitochondria (Figures 3(L,M), and 4(C–E)). Conversely, recombinant SFRP2 suppressed mitophagy and promoted mtROS production (Figure S4H-O). These results established SFRP2 as a critical suppressor of PINK1-mediated mitophagy in myofibroblasts.

### SFRP2 promoted myofibroblast fibrogenic activity by inhibiting PINK1-mediated mitophagy initiation

Impaired PINK1-mediated mitophagy drives pathological myofibroblast activation and pulmonary fibrosis progression [1,13]. To test whether SFRP2 exerts its pro-fibrotic effects through this pathway, we treated SFRP2-knockdown myofibroblasts with CQ, which restored expression of FN1, COL1A1 and ACTA2 (Figure 4(A-E)), indicating that SFRP2 promoted myofibroblast fibrogenic activity primarily through suppressing mitophagic flux. A decline in mitochondrial membrane potential (∆Ψm) promotes the accumulation of PINK1 to the mitochondrial outer membrane [27], then triggering the initiation of PINK1-mediated mitophagy. Previous studies have shown that SFRP2 inhibited the decline of ∆Ψm [15,16]. SFRP2 knockdown significantly reduced ΔΨm and enhanced mitochondrial PINK1 accumulation (Figure 4(F-I) And Figure S5A, B). Crucially, the inhibition of myofibroblast activation achieved by SFRP2 knockdown was reversed by concurrent PINK1 depletion (Figure 4(J-Q) and Figure S4C). These results established that SFRP2 promoted myofibroblast activation by inhibiting PINK1-mediated mitophagy initiation.

### SFRP2 promoted the translocation of PINK1 into mitochondria via the FZD5-mediated WNT-Ca^2+^ signaling pathway

**Figure 5.**
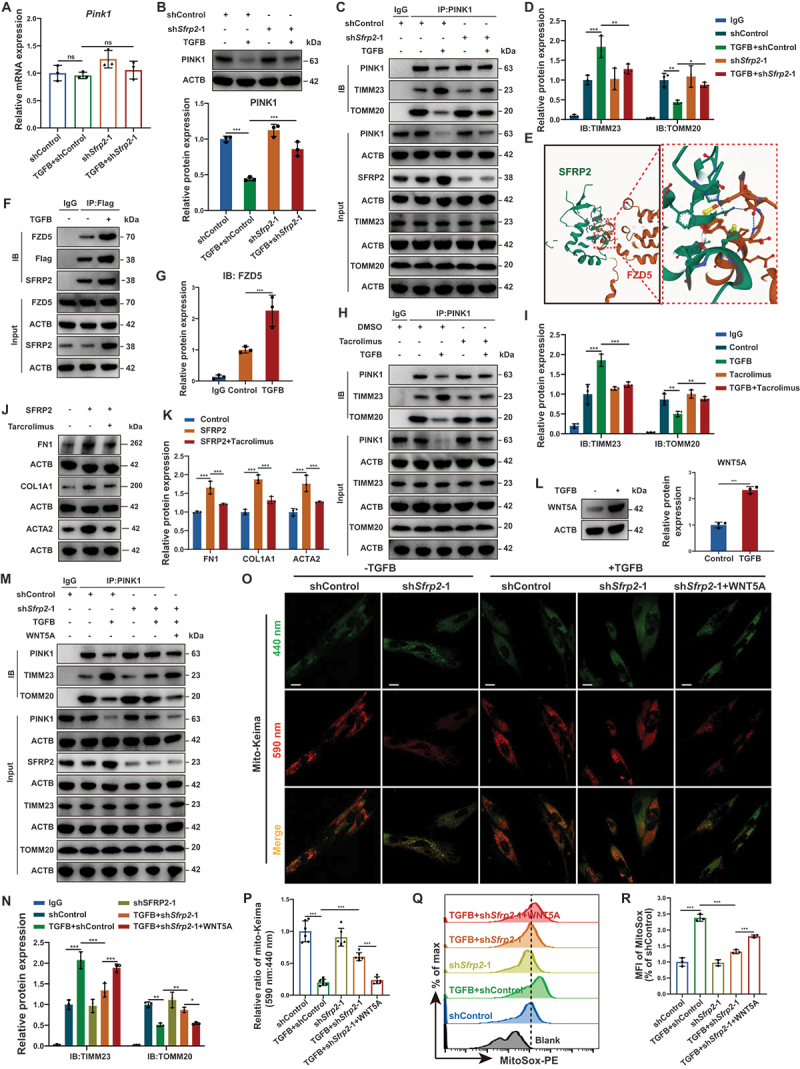
SFRP2 promoted the translocation of PINK1 into mitochondria via the FZD5-mediated WNT-Ca^2+^ signaling pathway. (**A**) qPCR analysis of *Pink1* mRNA levels in PMLFs activated by TGFB/TGF-β and subsequent SFRP2 knockdown (*n* = 3 biological repeats per group). (**B**) Western blotting analysis of PINK1 protein levels in PMLFs activated by TGFB/TGF-β and subsequent SFRP2 knockdown. Quantitative analysis of PINK1 protein levels normalized to ACTB (*n* = 3 biological repeats per group). (**C**) Immunoprecipitation (IP) using an anti-PINK1 antibody in PMLFs activated by TGFB/TGF-β and subsequent to SFRP2 knockdown (*n* = 3 biological repeats per group). (**D**) Quantitative analysis of immunoprecipitated TIMM23 and TOMM20 levels from (**C**) (*n* = 3 biological repeats per group). (**E**) AlphaFold3 analysis depicting the interaction between SFRP2 and FZD5. (**F**) IP using an anti-flag antibody in PMLFs activated by TGFB/TGF-β. (**G**) Quantitative analysis of immunoprecipitated FZD5 levels from (**F**) (*n* = 3 biological repeats per group). (**H**) IP using an anti-PINK1 antibody in PMLFs activated by TGFB/TGF-β and following tacrolimus treatment. (**I**) Quantitative analysis of immunoprecipitated TIMM23 and TOMM20 levels from (**H**) (*n* = 3 biological repeats per group). (**J**) Western blotting analysis of FN1, COL1A1, and ACTA2 expression in PMLFs treated with SFRP2 and tacrolimus. (**K**) Quantitative analysis of FN1, COL1A1, and ACTA2 protein levels normalized to ACTB (*n* = 3 biological repeats per group). (**L**) Western blotting analysis of WNT5A expression in PMLFs activated by TGFB/TGF-β. Quantitative analysis of WNT5A protein levels normalized to ACTB (*n* = 3 biological repeats per group). (**M**) IP using an anti-PINK1 antibody in PMLFs following TGFB/TGF-β activation, SFRP2 knockdown, and WNT5A treatment. (**N**) Quantitative analysis of immunoprecipitated TIMM23 and TOMM20 levels from (**M**) (*n* = 3 biological repeats per group). (**O**) Representative IF images of PMLFs transfected with mito-Keima lentiviral vectors following TGFB/TGF-β activation, SFRP2 knockdown, and WNT5A treatment. Fluorescence was detected at 440 nm (neutral pH) or 590 nm (acidic pH) to monitor mitophagic activity. Scale bars: 20 µm. (**P**) Quantitative analysis of the mitophagic flux index, presented as the ratio of 590 nm:440 nm fluorescence signals from (**O**) (*n* = 6 biological repeats per group). (**Q**) Flow cytometry analysis of mtROS levels in PMLFs following TGFB/TGF-β activation, SFRP2 knockdown, and WNT5A treatment. (**R**) Quantitative analysis of MFI of MitoSOX to assess mtROS levels from (**Q**) (*n* = 3 biological repeats per group). Data are presented as mean ± SD; ns, not significant; *: *p* < 0.05; **p < 0.01; ***p < 0.001.

To elucidate the mechanism whereby SFRP2 suppresses PINK1-mediated mitophagy, we first determined that SFRP2 knockdown elevated PINK1 protein, but not mRNA, levels in TGFB/TGF-β-induced myofibroblasts (Figure 5(A,B)), indicating post-translational regulation. As the cleavage and degradation of PINK1 depend on its translocation into the IMM [27,30], a process mediated by both the mitochondrial outer-membrane complex (TOMM) and the inner-membrane complex (TIMM) [31]. We found that SFRP2 knockdown diminished PINK1 interaction with TIMM23 while enhancing its binding to TOMM20, inhibiting the translocation of PINK1 into IMM in TGFB/TGF-β-induced myofibroblasts (Figure 5(C,D)).

We next explored the upstream signaling pathway. Given that SFRP2 activates the non-canonical WNT-Ca^2+^ signaling pathway via FZD5 in fibrotic microenvironments [17], we revealed SFRP2 levels positively correlated with non-canonical WNT-Ca^2+^ pathway (Figure S5D, E), and SFRP2 knockdown reduced cytosolic Ca^2+^ (Figure S5F, G). Intersection of the GO pathway “Regulation of Mitophagy” and “Noncanonical WNT Signaling Pathway” gene sets identified FZD5 as the sole overlapping component (Figure S5H). We confirmed that SFRP2 bound FZD5 and this interaction was increased in TGFB/TGF-β-induced myofibroblasts (Figure 5(E-G) and Figure S5I). Crucially, both FZD5 knockdown and pharmacological inhibition of calcineurin pathway with tacrolimus recapitulated the SFRP2 knockdown phenotype, similarly suppressed the translocation of PINK1 into IMM (Figure 5(H,I) and Figure S5J-K). Tacrolimus treatment effectively inhibited SFRP2-driven myofibroblast activation and prevented PINK1 translocation to the IMM, thereby rescuing mitophagy (Figure 5(J,K) and Figure S5L, M).

Furthermore, WNT5A, a non-canonical WNT ligand upregulated by TGFB/TGF-β (Figure 5(L)), promoted myofibroblast activation and suppressed mitophagy (Figure S5N-Q). Exogenous WNT5A reversed the effects of SFRP2 knockdown, restoring PINK1 translocation to the IMM, suppressing mitophagy, and promoting mtROS production (Figure 5(M-R)). Collectively, these results established a pathway in which SFRP2 binding to FZD5 activated a WNT-Ca^2+^-PPP3/calcineurin axis that directed PINK1 translocation into the IMM for degradation, thereby repressing mitophagy initiation.

### Suppression of SFRP2 alleviated pulmonary fibrosis in experimental mouse models

**Figure 6.**
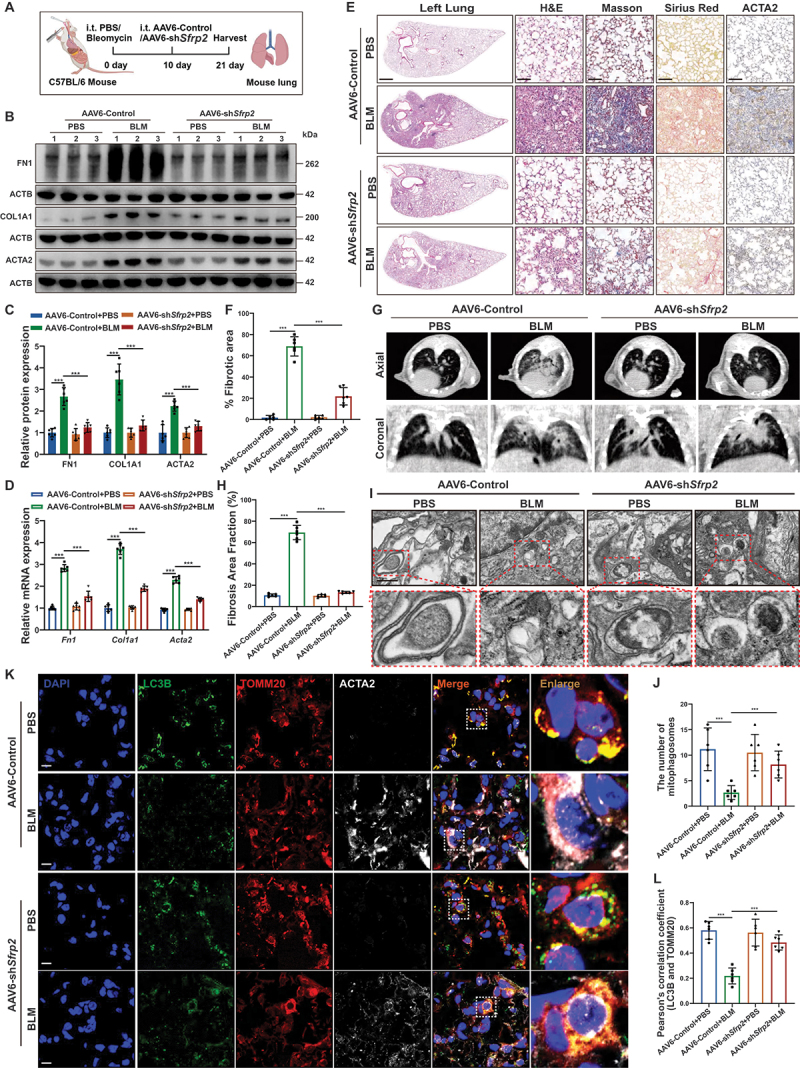
Suppression of SFRP2 alleviated pulmonary fibrosis in experimental mouse models. (**A**) Schematic diagram illustrating the treatment process for experimental mouse models induced by BLM or PBS with either AAV6-sh*Sfrp2* or AAV6-control treatment. (**B**) Western blotting analysis of FN1, COL1A1 and ACTA2 protein levels in the lungs of experimental mouse models treated with AAV6-sh*Sfrp2* or AAV6-control. (**C**) Quantitative analysis of FN1, COL1A1, and ACTA2 protein levels normalized to ACTB (*n* = 6 biological repeats per group). (**D**) qPCR analysis of *Fn1*, *Col1a1* and *Acta2* mRNA levels in the lungs of experimental mouse models treated with AAV6-sh*Sfrp2* or AAV6-control (*n* = 6 biological repeats per group). (**E**) Representative images of H&E staining, Masson’s trichrome staining, Sirius red staining, and IHC staining for ACTA2 in lung sections from experimental mouse models treated with AAV6-sh*Sfrp2* or AAV6-control. Scale bars: 600 µm and 100 µm. (**F**) Quantitative analysis of the fibrotic stroma area in H&E-stained lung sections, as shown in (**E**) (*n* = 6 biological repeats per group). (**G**) Representative axial and coronal micro-CT images of lungs from BLM-injured mice treated with either AAV6-sh*Sfrp2* or AAV6-control. (**H**) Quantitative analysis of fibrosis area fraction from micro-CT analysis in (**G**) (*n* = 6 biological repeats per group). (**I**) Representative TEM images of lung tissues from BLM-treated mice administered AAV6-sh*Sfrp2* or AAV6-control. Scale bars: 1 μm. (**J**) Quantitative analysis of mitophagosome counts from (**I**) (*n* = 6 biological replicates per group). (**K**) Representative IF images depicting the colocalization of LC3B (green) and TOMM20 (red) in *ACTA2*^+^ myofibroblasts (white) from the lungs of experimental mice treated with AAV6-sh*Sfrp2* or AAV6-control. scale bars: 5 µm. (**L**) Pearson correlation coefficient quantifying the colocalization of LC3B and TOMM20 (*n* = 6 biological repeats per group). Data are presented as mean±SD; ***: *p* < 0.001.

Building on the clinical potential of adeno-associated virus (AAV) vectors [32]. we investigated SFRP2 as a therapeutic target in experimental mouse models (Figure 6(A)). By day 21, AAV6-sh*Sfrp2* effectively reduced SFRP2 expression in lungs (Figure S6A-D) and significantly attenuated lung fibrosis in BLM-injured lungs (Figure 6(B-F) and Figure S6E). Micro-computed tomography (Micro-CT) imaging confirmed that SFRP2 knockdown mitigated BLM-induced lung damage (Figure 6(G,H)), while improving survival and weight recovery (Figure S6F, G). IF revealed reduced FN1, COL1A1 and ACTA2 staining in AAV6-sh*Sfrp2*-treated lungs (Figure S6H).

Based on our mechanistic findings linking SFRP2 to mitophagy suppression, we examined whether therapeutic efficacy involved pathway restoration. TEM analysis revealed increased mitophagosomes and decreased damaged mitochondria in AAV6-sh*Sfrp2*-treated lungs (Figure 6(I,J) and Figure S6L, M). Western bloting analysis showed elevated LC3B levels with or without CQ treatment in AAV6-sh*Sfrp2*-treated lungs, indicating enhanced autophagic flux (Figure S6I, J). SFRP2 knockdown increased PINK1 and PRKN while decreasing SQSTM1/p62, without altering mitochondrial mass (Figure S6K). Furthermore, AAV6-sh*Sfrp2* enhanced both expression and mitochondrial colocalization of LC3B and PINK1 in *ACTA2*^*+*^ myofibroblasts of BLM-injured lungs (Figure 6(K,L) And Figure S6N, O). These results demonstrated that AAV6-mediated SFRP2 knockdown therapeutically mitigated pulmonary fibrosis by specifically restoring mitophagic flux in activated myofibroblasts.

### SFRP2 neutralizing antibody alleviated pulmonary fibrosis in experimental mouse models

**Figure 7.**
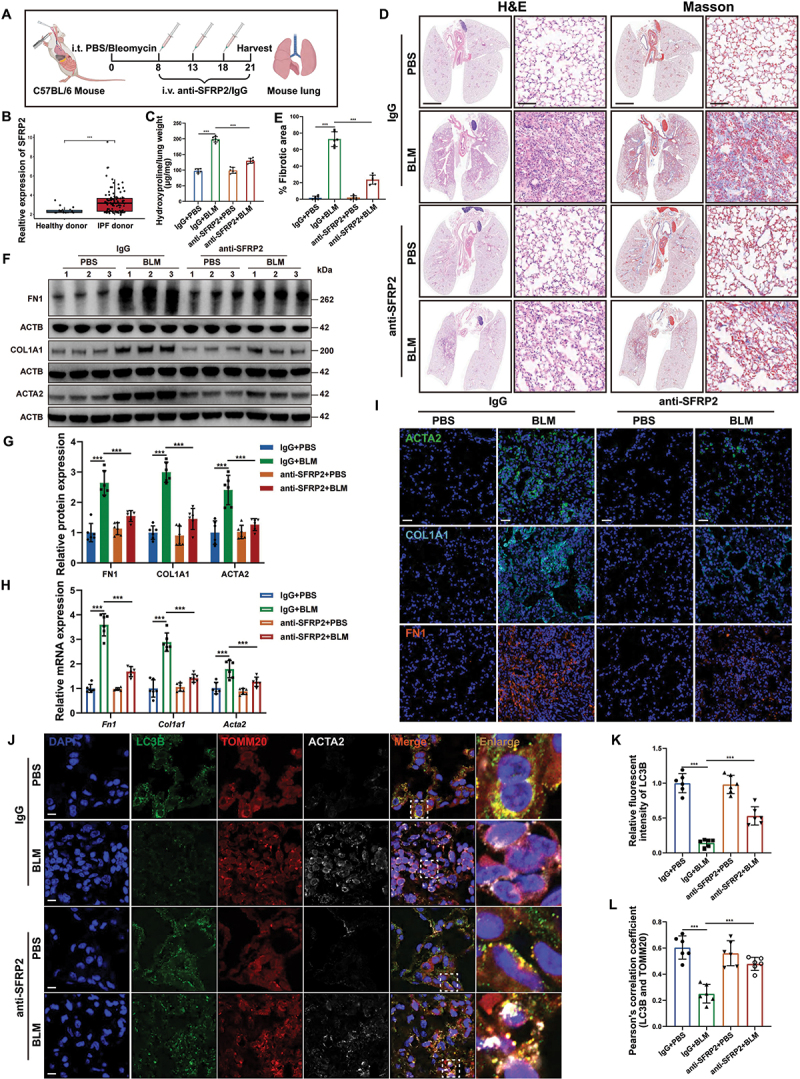
SFRP2 neutralizing antibodies alleviated pulmonary fibrosis in experimental mouse models. (**A**) Schematic illustration of the treatment process for experimental mice induced by BLM or PBS, with administration of anti-SFRP2 antibodies or IgG. (**B**) Analysis of BALF RNA-seq data from GSE70866 revealed significantly elevated SFRP2 expression in patients with IPF compared to healthy donors. (**C**) Hydroxyproline levels in lungs from experimental mouse models treated with anti-SFRP2 antibodies or IgG (*n* = 6 biological repeats per group). (**D**) H&E and Masson’s trichrome staining of lung sections from experimental mouse models treated with anti-SFRP2 antibodies or IgG. Scale bars: 2.5 mm and 100 µm. (**E**) Quantitative analysis of the fibrotic stroma area in H&E-stained lung sections, as shown in (**D**) (*n* = 6 biological repeats per group). (**F**) Western blotting analysis of FN1, COL1A1 and ACTA2 protein levels in the lungs of experimental mouse models treated with anti-SFRP2 antibodies or IgG. (**G**) Quantitative analysis of FN1, COL1A1 and ACTA2 protein levels normalized to ACTB from (**F**) (*n* = 6 biological repeats per group). (**H**) qPCR analysis of *Fn1*, *Col1a1* and *Acta2* mRNA levels in the lungs of experimental mouse models treated with anti-SFRP2 antibodies or IgG (*n* = 6 biological repeats per group). (**I**) Representative IF images of ACTA2, COL1A1, and FN1 in lung sections from experimental mouse models treated with anti-SFRP2 antibodies or IgG. Scale bars: 50 µm. (**J**) Representative IF images depicting the colocalization of LC3B (green) and TOMM20 (red) in *ACTA2*^+^ myofibroblasts (white) from the lungs of experimental mice treated with anti-SFRP2 antibodies or IgG. Scale bars: 5 µm. (**K**) Quantitative analysis of relative fluorescent intensity of LC3B from (**J**) (*n* = 6 biological repeats per group). (**L**) Pearson correlation coefficient quantifying the colocalization of LC3B and TOMM20 from (**J**) (*n* = 6 biological repeats per group). Data are presented as mean±SD; ***: *p* < 0.001.

Based on our findings that SFRP2 neutralizing antibody inhibited fibrogenic activity in TGFB/TGF-β-induced myofibroblasts, we evaluated its therapeutic potential in experimental pulmonary fibrosis (Figure 7(A)). Supporting the clinical relevance of this approach, bronchoalveolar lavage fluid (BALF) RNA-seq analysis showed significantly elevated SFRP2 expression in IPF patients compared to healthy controls (Figure 7(B)). Therapeutic administration of SFRP2 neutralizing antibody substantially attenuated lung fibrosis, as evidenced by improved lung architecture in histological staining (H&E) staining, reduced collagen deposition in Masson’s trichrome staining, and decreased hydroxyproline content (Figure 7(C-E)). The antibody treatment effectively suppressed expression of key fibrotic markers, including FN1, COL1A1, and ACTA2 in experimental pulmonary fibrosis models (Figure 7(F-I)). Mechanistically, the antibody restored mitophagic activity, demonstrated by enhanced LC3B expression and increased mitochondrial colocalization in *ACTA2*^+^ myofibroblasts (Figure 7(J-L)). Collectively, these results demonstrated that SFRP2 neutralizing antibody represented a promising therapeutic strategy for pulmonary fibrosis, acting through coordinated suppression of fibrotic gene expression and restoration of impaired mitophagy.

### Enhancing mitophagy through rapamycin administration alleviated pulmonary fibrosis in experimental mouse models

Accumulating evidence indicated that impaired mitophagy drives myofibroblast activation and pulmonary fibrosis progression [1,13,14]. Thus, restoring mitophagy in fibrotic lungs might offer a viable therapeutic strategy for this condition. To test this premise, we administered the established autophagy inducer rapamycin to BLM-treated mice (Figure S7A) [31]. Rapamycin treatment significantly reduced expression of the fibrotic markers, including FN1, COL1A1, and ACTA2 (Figure S7B-D), and attenuated lung fibrosis as evidenced by H&E, Masson’s trichrome, Sirius red and reduced hydroxyproline content (Figure S7E-G). Micro-CT imaging indicated that rapamycin effectively mitigated BLM-induced lung structural damage (Figure S7H, I). Moreover, rapamycin enhanced mitophagic activity, demonstrated by increased LC3B and PINK1 expression and their enhanced mitochondrial colocalization in *ACTA2*^+^ myofibroblasts (Figure S7K-M And Figure S8A-C). Collectively, these findings underscored the critical role of mitophagy in regulating pulmonary fibrosis and suggested that rapamycin might serve as a promising therapeutic strategy in this context.

## Discussion

IPF is a progressive, age-associated interstitial lung disease characterized by pathological ECM deposition that disrupts lung architecture and impairs respiratory function [2,33]. Myofibroblasts represent the primary effector cells responsible for excessive ECM production in IPF [3,34], while their targeted inhibition attenuates pulmonary fibrosis progression [1,4]. Our scRNA-seq analysis revealed that myofibroblasts derived from IPF patients exhibited significantly enhanced ECM synthesis and remodeling capabilities compared to those from healthy lung tissue, with SFRP2 specifically upregulated in this pathological population. SFRP2 has emerged as a pro-fibrotic mediator in various fibrotic diseases [7,21]. Kobayashi et al. first demonstrated SFRP2’s involvement in promoting cardiac fibrosis, with SFRP2 knockout mice exhibiting significantly reduced myocardial fibrosis [10]. SFRP2 promoted the proliferation of cardiac fibroblasts, contributing to excessive ECM production and the progression of cardiac fibrosis [7]. In systemic sclerosis, a progenitor fibroblast population identified by SFRP2 expression gives rise to dermal myofibroblasts [11]. However, its role in pulmonary fibrosis remains undefined. SFRP2 enhances cellular functions through autocrine/paracrine mechanism [23], facilitating breast cancer cell persistence in tumor microenvironments [8], and increasing melanoma cell invasiveness via aged fibroblast-derived signals [9]. However, its direct regulation of lung myofibroblast fibrogenic activity remains unexplored. Here, we demonstrated that SFRP2 knockdown significantly inhibited the fibrogenic activity of TGFB/TGF-β-induced myofibroblasts. Furthermore, hyperactive myofibroblasts secreted SFRP2 through an autocrine loop that reinforced their fibrogenic phenotype. These findings established SFRP2 as a critical mediator of lung myofibroblast activation and highlighted its therapeutic potential in IPF.

Mitochondrial dysfunction has emerged as a critical contributor to IPF pathogenesis, promoting myofibroblast activation and sustained fibrotic signaling through impaired energy metabolism, elevated oxidative stress, and disrupted cellular homeostasis [1,12,35]. These alterations stimulate profibrotic mediator secretion that exacerbates disease progression [2,14]. Notably, myofibroblasts from fibrotic lungs exhibit impaired mitophagy, particularly impaired PINK1-mediated mitophagy [1,4,13], which accelerates ECM production and pulmonary fibrosis [13,14]. While SFRP2 has been implicated in regulating intracellular oxidative stress by modulating mitochondrial dynamics and quality control in other contexts [15,16], its role in mitophagic regulation in pulmonary fibrosis remained unexplored. Here, we demonstrated that hyperactive myofibroblasts exhibited mitophagy impairment, and that SFRP2 knockdown restored PINK1-mediated mitophagy. Our findings established SFRP2 as a key suppressor of mitophagy in myofibroblasts and demonstrated a mechanism through which it drove myofibroblast fibrogenic activity.

PINK1-mediated mitophagy is a major pathway involved in the selective degradation of mitochondria [25]. Depolarized mitochondria stabilized PINK1 on the outer mitochondrial membrane, facilitating the recruitment of PRKN, LC3, and SQSTM1/p62 to trigger mitophagy initiation [27,31]. The cleavage and degradation of PINK1 depend on its translocation into the IMM [31]. In our study, we found that SFRP2 promoted the translocation of PINK1 into mitochondria to inhibit the initiation of mitophagy. As SFRP2 is known to activate noncanonical WNT-Ca^2+^ signaling via FZD5 in fibrotic microenvironments [17]. Our results demonstrated that inhibition of WNT-Ca^2+^ signaling pathway in TGFB/TGF-β-induced myofibroblasts restored PINK1-mediated mitophagy. Further investigation revealed that SFRP2 promoted the translocation of PINK1 into IMM through the FZD5-mediated WNT-Ca^2+^ signaling pathway to inhibit mitophagy initiation.

Current IPF therapeutics – pirfenidone and nintedanib – provide limited clinical benefit and are hampered by tolerability issues [36]. Lung transplantation stands as the only definitive cure for IPF. However, this option is unattainable for many patients due to age-related factors and comorbidities [37]. AAV vectors have emerged as promising therapeutic platforms, demonstrating both safety and clinical efficacy in pulmonary diseases [32]. Previous reports on AAV vectors carrying the cystic fibrosis transmembrane conductance regulator have demonstrated notable improvements in lung function for cystic fibrosis patients [37]. In this study, intratracheal AAV6-sh*Sfrp2* administration significantly attenuated fibrosis in mouse models, indicating the therapeutic effect of AAV-sh*Sfrp2* on pulmonary fibrosis. Moreover, we identified that SFRP2-neutralizing antibody significantly inhibited fibrogenic activity in vitro and in vivo, and rapamycin-mediated mitophagy enhancement similarly attenuated lung fibrosis. These findings collectively highlighted the autocrine SFRP2-mitophagy axis as a novel therapeutic target for pulmonary fibrosis. Further clinical trials are warranted to explore their clinical efficacy.

While our study establishes mitophagy impairment as a key feature of pulmonary fibrosis, we acknowledge limitations in pharmacological approaches. CQ (a lysosomal inhibitor) and rapamycin (a general autophagy inducer) affect broader autophagic processes beyond mitophagy. Thus, while our data using these compounds support altered mitophagic activity, the observed effects may partially derive from modulation of general autophagy. Future studies employing genetic or more specific mitophagy modulators will help refine these mechanistic insights.

## Conclusion

**Figure 8.**
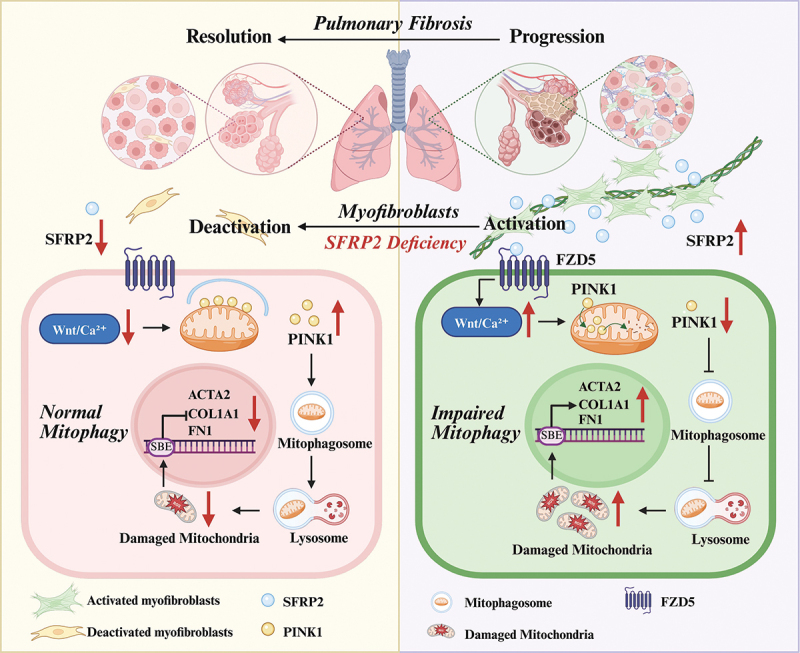
Schematic illustration of the mechanism by which autocrine SFRP2 enhances myofibroblast fibrogenic activity through suppressing PINK1-mediated mitophagy initiation. In fibrotic lung microenvironments, hyperactivated myofibroblasts secreted SFRP2 via autocrine signaling, driving pathological fibrosis by suppressing PINK1-mediated mitophagy. Mechanistically, SFRP2 promoted the translocation of PINK1 into mitochondrial through FZD5-mediated WNT-Ca^2+^ signaling, thereby inhibiting mitophagy initiation. Furthermore, SFRP2 deficiency reduced fibrotic activity in myofibroblasts and attenuated pulmonary fibrosis in vivo, demonstrating the therapeutic potential of targeting autocrine SFRP2-mitophagy axis.

In this study, we revealed that myofibroblasts derived from patients with IPF exhibited markedly enhanced capabilities for ECM synthesis and remodeling compared to those in healthy lung tissue. SFRP2 was specifically highly expressed in myofibroblasts derived from fibrotic lung tissue. Through in vitro experiments, we demonstrated that hyperactive myofibroblasts secreted SFRP2 via an autocrine mechanism, which promoted myofibroblast fibrotic activity by inhibiting PINK1-mediated mitophagy initiation. Our investigation also revealed the essential role of SFRP2 in promoting the translocation of PINK1 into IMM to inhibit mitophagy initiation via the FZD5-mediated WNT-Ca^2+^ signaling pathway (Figure 8). Utilizing AAV6-sh*Sfrp2*, SFRP2 neutralizing antibody and rapamycin administration in experimental pulmonary fibrosis mouse models, we identified autocrine SFRP2-mitophagy axis as a novel therapeutic target for pulmonary fibrosis.

## Materials and methods

### Acquisition and analysis of scRNA-seq and bulk data

All single-cell and bulk data were retrieved from the Gene Expression Omnibus (GEO). The scRNA-seq data, including gene expression and cell clusters in both IPF and healthy donor lungs, was obtained by integrating the GSE132771 and GSE128033 datasets [19,20]. The data were processed using the “Seurat” package (version 4.0.5) and “SCP,” following their published guidelines (https://github.com/satijalab/seurat; https://github.com/zhanghao-njmu/SCP). The bulk data for IPF and healthy donor lungs were extracted from the GSE32537 dataset. The BALF RNA-seq data were extracted from GSE70866.

### Animal experiments

All animal procedures conducted in this study were approved by the Ethics Committee of Sun Yat-sen University. Male C57BL/6 mice (aged 8–12 weeks; Guangdong Yaokang Biotechnology Co., LTD) were housed in a specific pathogen-free (SPF) animal laboratory, with a 12:12 h light/dark cycle at 22–24°C. Pulmonary fibrosis was induced by intratracheal administration of either BLM (Teva Pharmaceutical, 00703–3155-01; 3 U/kg) or AdTGFB/TGF-β1 (1.5 × 10^1 2^ viral genomes) with tissues harvested at day 21 post-induction. For therapeutic interventions, mice received intratracheal AAV6-sh*Sfrp2* or AAV6-Control (1.5 × 10^1 2^ viral genomes) at day 10 post-induction, intravenous anti-SFRP2 antibody (EMD Millipore, MABC539; 10 mg/kg every 5 days from day 8), or daily intraperitoneal rapamycin (8 mg/kg from day 8), with all endpoints assessed at day 21.

### Patient samples

Human lung tissues with fibrosis were obtained from patients meeting the diagnostic criteria for IPF via diagnostic surgical lung biopsies at the First Affiliated Hospital of Guangzhou Medical University (Guangzhou, China). Control lung tissue samples were collected from patients undergoing surgical lung resection for lung cancer at the same hospital. All participants provided written informed consent for research use of their samples, and the study protocol was approved by the Committees for Ethical Review of Research (2018–85).

### Micro-CT analysis

Mice were initially anesthetized with isoflurane and meticulously positioned to ensure optimal conditions for thorough lung scanning. Subsequently, we utilized the Inveon micro-CT Imaging system (Siemens, Germany) to acquire high-resolution CT scans of the lungs. Pulmonary fibrosis was quantified from obtained Micro-CT images using ImageJ, expressed as fibrosis area fraction (percentage of fibrotic area relative to total lung area).

### Histopathological, immunohistochemical (IHC) and IF analysis

The lung tissues were initially fixed in a 4% paraformaldehyde solution, dehydrated, and then subjected to paraffin embedding. Subsequently, paraffin-embedded lungs were sectioned into 10-μm-thick tissue sections for further experiments. To assess the extent of lung fibrosis, we employed H&E, Masson trichrome, and Sirius Red staining [3]. For immunohistochemical (IHC) analysis, lung tissue sections were incubated with primary antibody overnight at 4°C. Following incubation with secondary antibody, signal modulation and amplification were achieved using DAB (MXB, MAX-001). Semi-quantitative analyses of SFRP2 staining were conducted using five non-overlapping lung tissue views through ImageJ software. IHC images were captured using a fully automated inverted fluorescence microscope (DMI8). For IF analysis, lung tissue sections were incubated overnight at 4°C with primary antibodies against the indicated proteins, and then with Alexa-Fluor-conjugated secondary antibodies for 1 h at room temperature. The Zeiss 800 Laser Scanning Confocal Microscope, along with a Zeiss 880 Laser Scanning Confocal Microscope with Airyscan, was employed for IF images acquisition. Colocalization analyses of IF images were conducted using Image J software. The primary antibodies were as follows: ACTA2 (Abcam, ab7817), COL1A1 (Servicebio, GB11022-3–100), FN1 (Abcam, ab2413), LC3B (Cell Signaling Technology, 43,566), PINK1 (Proteintech, 23,274–1-AP), PRKN (Proteintech, 14,060–1-AP), SFRP2 (Proteintech, 66,328–1-Ig), TOMM20 (Proteintech, 11,802–1-AP).

### Western blotting analysis

As outlined above, lung tissues or cells were homogenized in RIPA lysis buffer (Sigma, R0278) containing protease and phosphatase inhibitors (Roche, 04906845001). Following BCA analysis, equal protein amounts were loaded into SDS-PAGE gel and subsequently transferred to PVDF membranes. The membranes were then incubated with the respective primary and secondary antibody. After detecting the target protein, the membrane was thoroughly stripped and then re-probed for the loading control, ensuring accurate and specific normalization for every protein target. Protein bands were detected with a ChemiDoc imaging system and quantified using ImageJ software. The primary antibodies were as follows: ACTA2 (Abcam, ab7817), ACTB (Cell Signaling Technology, 4970), COL1A1 (Servicebio, GB114197-100), Flag (Proteintech, 20,543–1-AP), FN1 (Abcam, ab2413), FZD5 (Proteintech, 21,519–1-AP), LC3B (Cell Signaling Technology, 43,566), PINK1 (Proteintech, 23,274–1-AP), PRKN (Proteintech, 14,060–1-AP), SQSTM1/p62 (Servicebio, GB11531-100), SFRP2 (Proteintech, 66,328–1-Ig), TIMM23 (Proteintech, 11,123–1-AP), TOMM20 (Proteintech, 11,802–1-AP).

### Quantitative real-time PCR (qPCR) analysis

Total RNA was extracted from lung tissues or cells using the TRIzol reagent (Molecular Research Center, TR118) following the manufacturer’s protocol. Subsequently, reverse transcription into cDNA was carried out with the NovoScript®Plus All-in-one 1st Strand cDNA Synthesis SuperMix Kit (NovoProtein, E047-01A). For Quantitative real-time PCR (qPCR) analysis, the LightCycler480 SYBR Green I Master Mix (Roche, 4,887,352,001-1) was utilized. The primer sequences employed are provided in Table S1.

### Cells isolation and culture

PMLFs were isolated from lung tissues following the protocol described in previous studies [1,3]. Briefly, lungs were sectioned into small fragments and digested in DMEM supplemented with dispase (10 mg/ml; Sigma-Aldrich, D4693) and collagenase type I (20 mg/ml; Gibco, Thermo Fisher Scientific, 9001–12-1) at 37°C for 1 h. After digestion, the mixture was filtered through 100-μm- and 40-μm-pore filters. The filtered cells were suspended in DMEM containing 10% fetal bovine serum (FBS), 100 KU/L penicillin and 100 mg/L streptomycin (Thermo Fisher Scientific, 15,140,122), and then cultured in a 5% CO_2_ incubator at 37°C. The culture medium was refreshed every two days, and cells at passages 3–6 were utilized for subsequent experiments. We identified lung myofibroblasts based on the expression of FN1, COL1A1, and ACTA2. The MRC5 cells were procured from the American Type Culture Collection (ATCC, CCL-171) and maintained following the supplier’s specifications. The induction of myofibroblast differentiation and formation were achieved by treating cells with TGFB/TGF-β (10 ng/ml; R&D, 240-B-002 and 7666-MB-005) for 48 h. To evaluate the function of SFRP2, the cells were exposed to SFRP2 recombinant protein (20 nM; GLPBIO, GP26334 and GP24557) for 48 h, as previously described [10].

### Transwell migration and invasion assay

Transwell assays were performed as described previously [38]. Cell migration and invasion were assessed using 24-well Transwell inserts (Falcon, 353,097) with 8 μm pores. For migration assays, myofibroblasts were seeded into the upper chamber at a density of 5 × 10^5^ cells/ml in serum-free medium, while the lower chamber contained 500 μl of complete medium with 10% FBS. For invasion assays, the upper chamber was pre-coated with Matrigel (Corning, 354,234) diluted in serum-free medium for 2 h prior to cell seeding at the same density, while the lower chamber contained 500 μl of complete medium with 10% FBS. After 24 h of incubation in a CO_2_ incubator, the cells in the upper chamber were removed, and those in the lower chamber were stained with crystal violet. Image acquisition was performed using a DMI8 microscope.

### Plasmids and transfection

Mouse and human shRNAs targeting SFRP2, PINK1 and FZD5 were procured from Guangzhou Compound Energy Gene Co., Ltd. Transient transfections of plasmids followed the manufacturer’s protocol. The efficacy of SFRP2, PINK1 and FZD5 knockdown was assessed through western blotting and qPCR.

### Monitoring mitophagy using mito-Keima

Mito-Keima is a pH-sensitive fluorescent protein that changes its excitation wavelength from 440 nm (neutral pH) to 590 nm (acidic pH) when mitochondria are engulfed by lysosomes during mitophagy. This shift allows for the quantitative analysis of mitophagy in cells. To assess mitophagy, cells were transduced with mito-Keima lentivirus (RiboBio, China) at an MOI of 5–10 in the presence of 8 μg/ml polybrene. Briefly, 5 × 10^4^ cells/well were seeded in 24-well plates 24 h prior to transduction. Viral supernatant was diluted in complete medium and applied to cells for 6 h at 37°C, then cultured in fresh complete medium with puromycin for 48 h before analysis. Confocal microscopy was used to image cells, exciting mito-Keima at 440 nm and 590 nm and collecting the corresponding emission signals. The fluorescence ratio of 590:440 nm was calculated to quantify mitophagy. For each biological replicate, a minimum of 100 cells per condition were analyzed.

### TEM analysis

Cells and lung tissues were fixed using a solution containing 3% glutaraldehyde and 2% paraformaldehyde in sodium cacodylate buffer at pH 7.3, as previously described. Following post fixation and dehydration, samples were infiltrated with Spurr’s resin and embedded in BEEM capsules. Ultrathin sections (60–80 nm) were prepared using an ultramicrotome and mounted on copper grids. The sections were stained with uranyl acetate and lead citrate to enhance contrast. The grids were examined under a transmission electron microscope operating at 80 kV, and images were captured with a digital camera system. Morphometric analysis of mitochondrial size was performed using ImageJ software. Mitochondrial damage in vitro and in vivo was quantified by TEM. From each sample, five randomly selected grid squares were quantified, yielding at least ten micrographs per square. Mitochondria displaying abnormal ultrastructure – swollen profiles, fractured or absent cristae, or vacuolated matrices – were identified. The percentage of damaged mitochondria was calculated as (number of damaged mitochondria ÷ total mitochondria counted) × 100%.

### Measurement of ∆ψm

∆Ψm was assessed using JC-1 detection kit (Beyotime, C2006). Under conditions of low ∆Ψm, JC-1 remains as a monomer rather than aggregating within the mitochondrial matrix, emitting green fluorescence. Fluorescence was measured using flow cytometry (CytoFLEX, Beckman Coulter, USA), and data were analyzed with FlowJo software.

### Measurement of mtROS

The mitochondrial superoxide detection kit (Beyotime, S0061M) was utilized to measure mtROS. MitoSOX Red is specifically oxidized by superoxide, with the resulting oxidation product binding to nucleic acids and generating a strong red fluorescence signal. In this procedure, cells were initially digested and collected, then incubated with the MitoSOX Red staining solution at 37°C for 30 min. Following incubation, the cells were washed to remove excess dye and subsequently resuspended in an appropriate volume of PBS. Finally, the samples were analyzed using a flow cytometer to quantify mtROS levels.

### Co-immunoprecipitation analysis

Cells were harvested and lysed in a suitable lysis buffer. The resulting lysate was incubated with agarose beads (without antibody; Thermo Fisher Scientific, 78,610) to eliminate nonspecific binding proteins. Following this, specific antibody was added to the pre-cleared lysate and allowed to incubate, facilitating the binding of antibody to the target protein and its interacting partners. To capture the antibody-protein complexes, protein A or G agarose beads were introduced into the mixture. The purified complexes were then analyzed using western blotting to confirm interactions.

### Statistical analysis

The data are expressed as the mean ± SD. For comparisons between two groups, unpaired t-tests were conducted. For statistical analyses involving multiple groups, one-way ANOVA with Tukey’s multiple comparison test was employed. GraphPad Prism software was used for all data analyses, and statistical significance was defined as *p* < 0.05.

## Supplementary Material

Supplementary materials.docx

Supplementary_Data R4.docx

## Data Availability

We authors declare all data and materials are available on request
