# Supplementary material for: Autocrine SFRP2 (secreted frizzled related protein 2) enhances lung myofibroblast fibrogenic activity by suppressing PINK1-mediated mitophagy initiation
Source: Autophagy. 2026 Mar 15;22(6):1333–50. doi: 10.1080/15548627.2026.2642341 (PMC13185440; doi:10.1080/15548627.2026.2642341)
Supplement: Supplementary materials.docx [file KAUP_A_2642341_SM1898.docx]

**Table S1.** Primer sequences for mRNA expression analysis.

| **Gene** | **Primer sequences** |
| --- | --- |
| Mouse *Acta2* | Forward:5’-TGAGACCTTCAATGTCCCCGC-3’  Reverse:5’-TCACACCATCTCCAGAGTCCAGC-3’ |
| Mouse *Col1a1* | Forward: 5’-CCCAGAGTGGAACAGCGATT-3’  Reverse:5’-ATGAGTTCTTCGCTGGGGTG -3’ |
| Mouse *Fn1* | Forward:5’-ATGTGGACCCCTCCTGATAGT -3’  Reverse:5’-GCCCAGTGATTTCAGCAAAGG-3’ |
| Mouse *Pink1* | Forward:5’-GGCTTCCGTCTGGAGGATTAT-3’  Reverse:5’-AACCTGCCGAGATATTCCACA-3’ |
| Mouse *Sfrp2* | Forward:5’-GAAGCTCCCAAGGTGTGTGA-3’  Reverse:5’TCACTTTGATTTTCAGTGCGAAGT-3’ |
| Mouse *Rn18s* | Forward:5’-GTGACGTTGACATCCGTAAAGA-3’  Reverse:5’-GCCGGACTCATCGTACTCC-3’ |
| Human *ACTA2* | Forward:5’-AAAAGACAGCTACGTGGGTGA-3’  Reverse:5’-GCCATGTTCTATCGGGTACTTC-3’ |
| Human *COL1A1* | Forward:5’-GAGGCTTCCCTGGTCTTCCT-3’  Reverse:5’-CTCACGTCCAGATTCACCAG-3’ |
| Human *FN1* | Forward:5’-CGGTGGCTGTCAGTCAAAG-3’  Reverse:5’-AAACCTCGGCTTCCTCCATAA-3’ |
| Human *GAPDH* | Forward:5’-GTCGGAGTCAACGGATTT-3’  Reverse:5’-GGAATCATATTGGAACATGTAAACC-3’ |
| Human *SFRP2* | Forward:5’-AACCTACATCAACCGAGATACCA-3’  Reverse:5’-CTTCAGGTCCCTTTCGGACAC-3’ |

**Supplementary Figures**

**
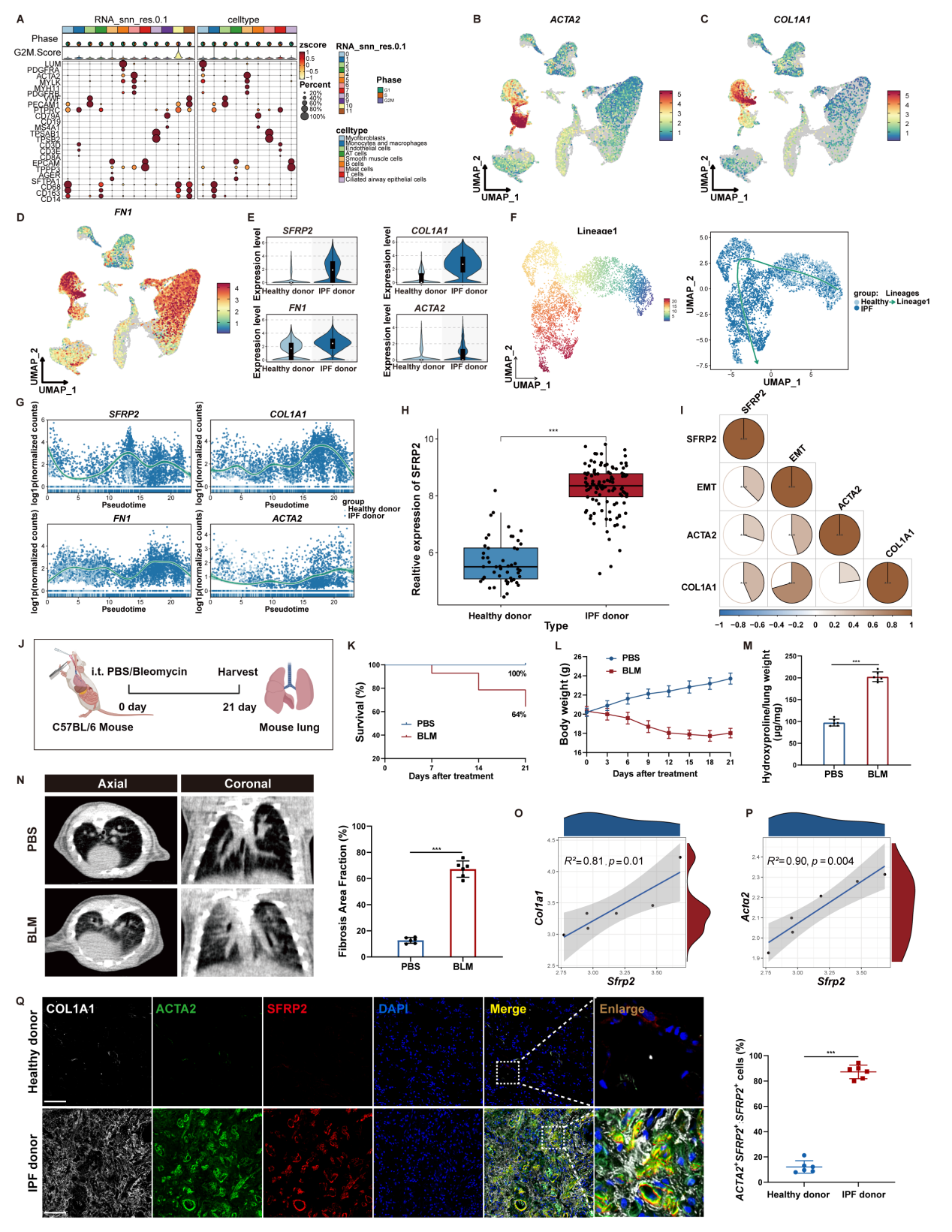
**

**Figure S1.** SFRP2 was specifically highly expressed in myofibroblasts derived from fibrotic lung tissue. (**A**) Heatmap of marker genes for all nine identified cell clusters in the single-cell RNA-sequencing (scRNA-seq) data, combining datasets from GSE132771 and GSE128033. (**B-D**) UMAP visualizations of *ACTA2*, *COL1A1*, and *FN1* expression in the lungs of idiopathic pulmonary fibrosis (IPF) donors and healthy donors using the scRNA-seq data (integrating GSE132771 and GSE128033). (**E**) Comparison of gene levels of *SFRP2*, *COL1A1*, *FN1*, and *ACTA2* between myofibroblasts derived from the lungs of IPF donors and healthy donors using the scRNA-seq data (integrating GSE132771 and GSE128033). (**F**) Pseudotime trajectory analysis of scRNA-seq data (integrating GSE132771 and GSE128033) revealing a continuum of myofibroblast phenotypes, ranging from those predominantly found in healthy donors to archetypal myofibroblasts enriched in IPF donors, mapped along diffusion pseudotime (DPT) distances. (**G**) Assessment of gene expression levels of *SFRP2*, *COL1A1*, *FN1*, and *ACTA2* in myofibroblasts along the DPT using the scRNA-seq data (integrating GSE132771 and GSE128033). (**H**) Differential expression analysis of *SFRP2* between IPF donor and healthy donor lungs in bulk data from GSE32537. (**I**) Correlation analysis among *SFRP2*, *ACTA2*, and *COL1A1* in bulk data (GSE32537). (**J**) Schematic illustration of the induction of pulmonary fibrosis mouse models following bleomycin (BLM) administration. (**K**) Survival rates of experimental mice treated with BLM or PBS monitored over a 21-day period (n = 14 mice per group). (**L**) Changes in body weight of experimental mice treated with BLM or PBS monitored over a 21-day period (n = 14 mice per group). (**M**) Hydroxyproline levels in lungs of experimental mice treated with BLM or PBS (n=6 biological repeats per group). (**N**) Representative axial and coronal micro-CT images of lungs from mice treated with BLM or PBS. Quantitative analysis of fibrosis area fraction derived from micro-CT analysis (n = 6 biological repeats per group). (**O**) Linear regression analysis between *Sfrp2* and *Col1a1* expression in the lungs of experimental mice treated with BLM (n = 6 biological repeats per group). (**P**) Linear regression analysis between *Sfrp2* and *Acta2* expression in the lungs of experimental mice treated with BLM (n = 6 biological repeats per group). (**Q**) Representative immunofluorescence (IF) images of COL1A1 (white), SFRP2 (red) and ACTA2 (green) in lung sections from IPF donors and healthy donors. Scale bars: 50 µm. Semiquantitative scoring of the percentage of *ACTA2*^+^*SFRP2*^+^ cells relative to *SFRP2*^+^cells, as obtained from IF images (n = 6 biological repeats per group). Data are presented as mean±SD; *: *p* < 0.05; ***: *p* < 0.001.


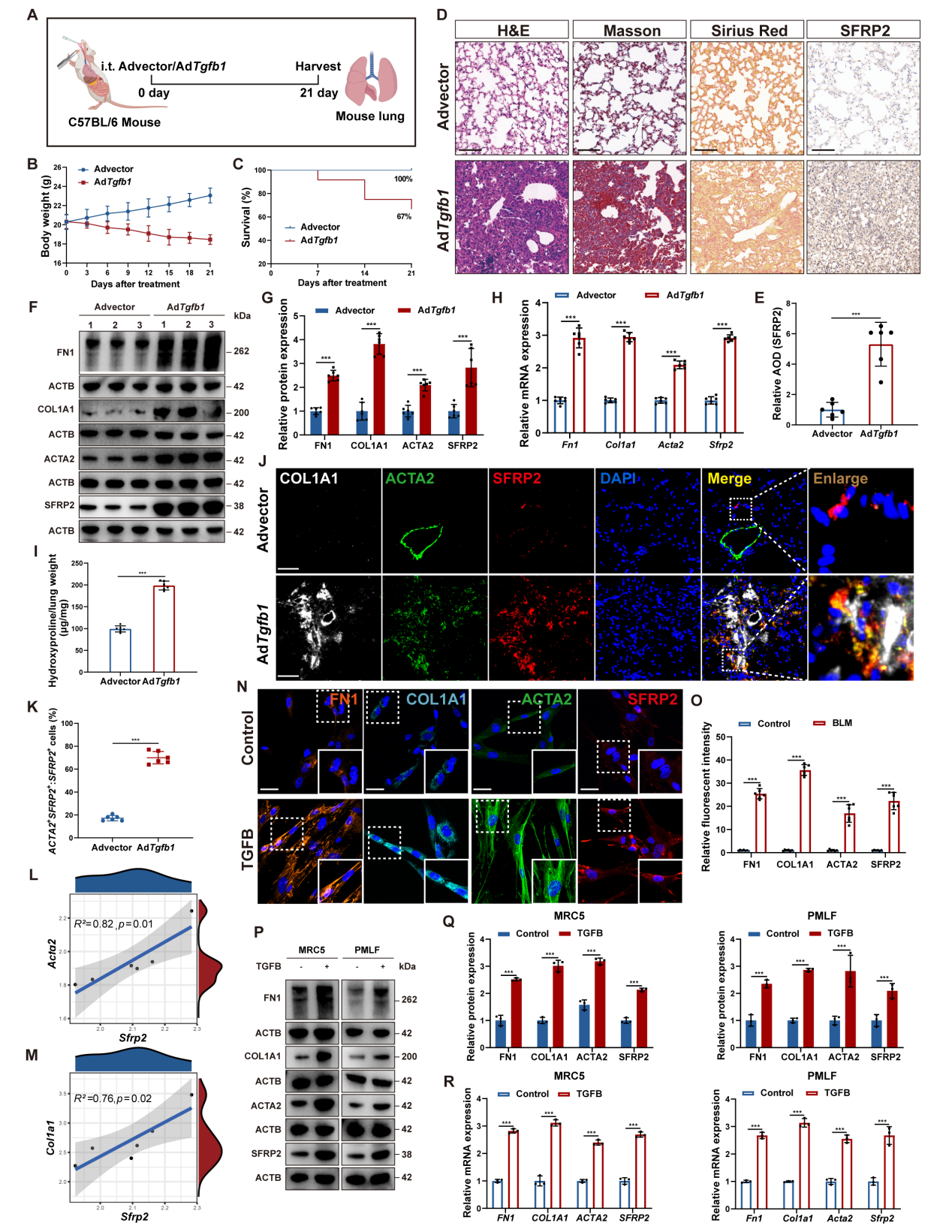


**Figure S2.** SFRP2 was specifically highly expressed in myofibroblasts derived from fibrotic lung tissue. (**A**) Schematic illustration of the induction of pulmonary fibrosis mouse models following administration of adenovirus TGFB1/TGF-β1 (Ad*Tgfb1*) or control adenovector (Advector). (**B**) Changes in body weight of experimental mice treated with Ad*Tgfb1* or Advector, monitored over a 21-day period (n = 12 mice per group). (**C**) Survival rates of experimental mice treated with Ad*Tgfb1* or Advector, monitored over a 21-day period (n = 12 mice per group). (**D**) Representative images of hematoxylin and eosin (H&E) staining, Masson’s trichrome staining, Sirius red staining, and immunohistochemical (IHC) staining for SFRP2 in lung sections from experimental mice treated with Advector or Ad*Tgfb1*. Scale bars: 100 µm. (**E**) Quantitative analysis of the average optical density (AOD) of SFRP2 in IHC images from the lungs of experimental mice treated with Advector or Ad*Tgfb1*1 (n = 6 biological repeats per group). (**F**) Western blotting analysis of FN1, COL1A1, ACTA2, and SFRP2 protein levels in the lungs of experimental mice treated with Advector or Ad*Tgfb1*. (**G**) Quantitative analysis of FN1, COL1A1, ACTA2, and SFRP2 protein levels normalized to ACTB from (**F**) (n = 6 biological repeats per group). (**H**) qPCR analysis of *Fn1*, *Col1a1*, *Acta2*, and *Sfrp2* mRNA levels in the lungs of experimental mice treated with Advector or Ad*Tgfb1* (n = 6 biological repeats per group). (**I**) Hydroxyproline levels in lungs of experimental mice treated with Advector or Ad*Tgfb1* (n=6 biological repeats per group). (**J**) Representative IF images of COL1A1 (white), SFRP2 (red) and ACTA2 (green) in lung sections from experimental mice treated with Advector or Ad*Tgfb1*. Scale bars: 50 µm. (**K**) Semiquantitative scoring of the percentage of *ACTA2*^+^*SFRP2*^+^ cells relative to *SFRP*2^+^cells, as obtained from IF images (n = 6 biological repeats per group). (**L**) Linear regression analysis between *Sfrp2* and *Acta2* expression in the lungs of experimental mice treated with Ad*Tgfb1* (n = 6 biological repeats per group). (**M**) Linear regression analysis between *Sfrp2* and *Col1a1* expression in the lungs of experimental mice treated with Ad*Tgfb1* (n = 6 biological repeats per group). (**N**) Representative IF images of primary mouse lung fibroblasts (PMLFs) stained for FN1, COL1A1, ACTA2, and SFRP2 following TGFB/TGF-β activation. Scale bars: 50 µm. (**O**) Quantitative analysis of relative fluorescent intensity of FN1, COL1A1, ACTA2, and SFRP2 from (**N**) (n = 6 biological repeats per group). (**P**) Western blotting analysis of FN1, COL1A1, ACTA2, and SFRP2 protein levels in MRC5 cells and PMLFs activated by TGFB/TGF-β. (**Q**) Quantitative analysis of FN1, COL1A1, ACTA2, and SFRP2 protein levels normalized to ACTB from (**P**) (n = 3 biological repeats per group). (**R**) qPCR analysis of *Fn1*, *Col1a1*, *Acta2*, and *Sfrp2* mRNA levels in PMLFs and MRC5 cells activated by TGFB/TGF-β (n = 3 biological repeats per group). Data are presented as mean±SD; ***: *p* < 0.001.

**
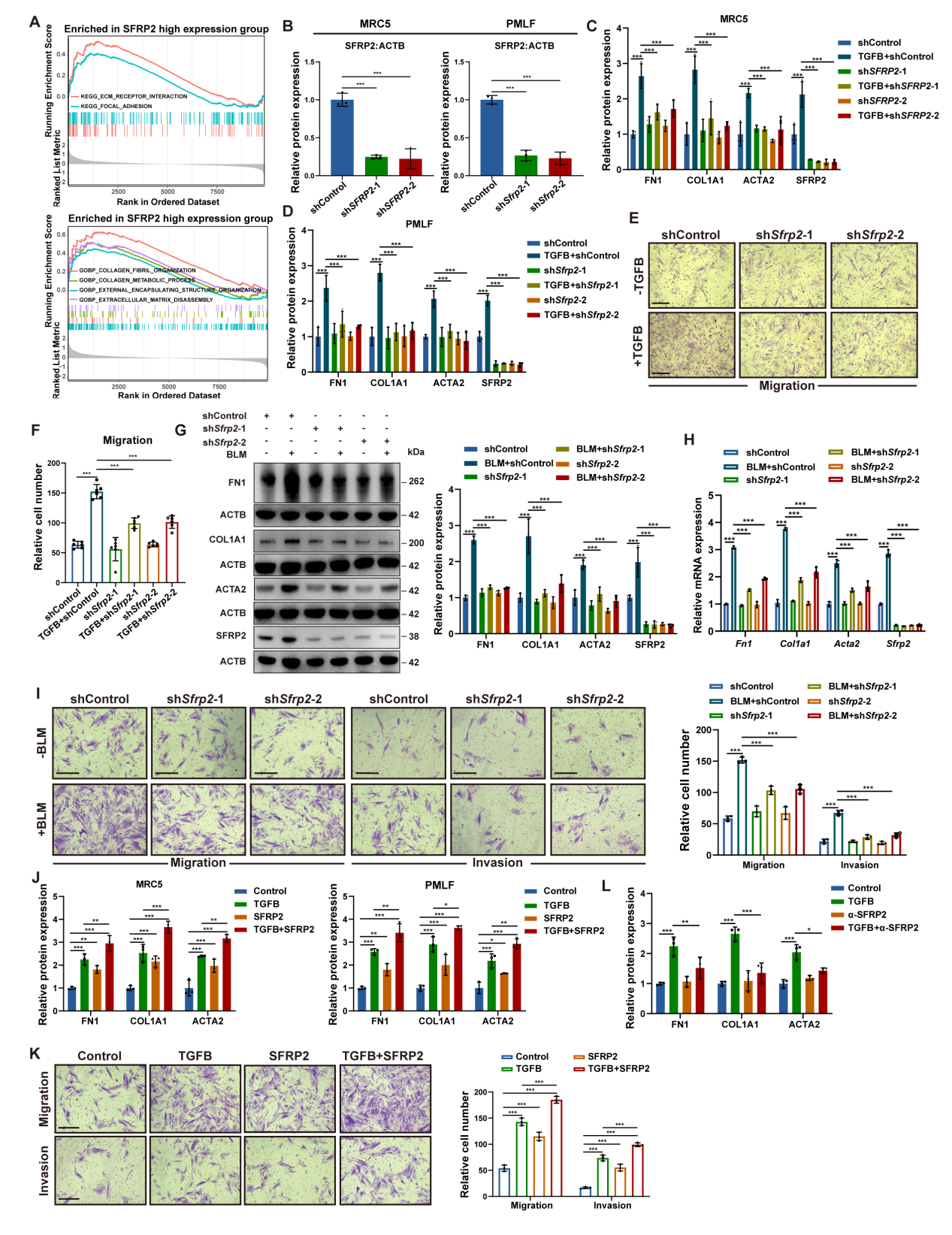
**

**Figure S3.** SFRP2 promoted myofibroblast fibrogenic activity through an autocrine mechanism. (**A**) Gene Set Enrichment Analysis (GSEA) of KEGG and GO gene sets revealed a positive correlation between high SFRP2 expression and the extracellular matrix (ECM) synthesis and remodeling pathways. (**B**) Quantitative analysis of SFRP2 protein level normalized to ACTB in MRC5 cells and PMLFs following SFRP2 knockdown (n = 3 biological repeats per group). (**C-D**) Quantitative analysis of FN1, COL1A1, ACTA2 and SFRP2 protein levels normalized to ACTB in MRC5 cells and PMLFs activated by TGFB/TGF-β and subsequently subjected to SFRP2 knockdown (n = 3 biological repeats per group). (**E**) Representative transwell images showing the migratory capabilities of PMLFs activated by TGFB/TGF-β and subsequently subjected to SFRP2 knockdown. Scale bars: 100 µm. (**F**) Quantitative analysis of migratory capabilities of PMLFs activated by TGFB/TGF-β and subsequently subjected to SFRP2 knockdown (n = 6 biological repeats per group). (**G**) Western blotting analysis and quantitative analysis of FN1, COL1A1, ACTA2 and SFRP2 protein levels in PMLFs from experimental mice treated with BLM or PBS, and following SFRP2 knockdown (n = 3 biological repeats per group). (**H**) qPCR analysis of *Fn1*, *Col1a1*, *Acta2* and *Sfrp2* mRNA levels in PMLFs from experimental mice treated with BLM or PBS, and following SFRP2 knockdown (n = 3 biological repeats per group). (**I**) Representative images and quantitative analysis of transwell assays showing the migratory and invasive capabilities of PMLFs derived from experimental mice treated with BLM or PBS, and following SFRP2 knockdown (n = 3 biological repeats per group). Scale bars: 100 µm. (**J**) Quantitative analysis of FN1, COL1A1, and ACTA2 protein levels normalized to ACTB in MRC5 cells or PMLFs activated by TGFB/TGF-β and treated with recombinant SFRP2 (n = 3 biological repeats per group). (**K**) Representative images and quantitative analysis of transwell assays showing the migratory and invasive capabilities of PMLFs activated by TGFB/TGF-β and treated with recombinant SFRP2 (n = 3 biological repeats per group). Scale bars: 100 µm. (**L**) Quantitative analysis of migratory and invasive capabilities of PMLFs activated by TGFB/TGF-β and treated with α-SFRP2 (n = 3 biological repeats per group). Data are presented as mean±SDs; *: *p* < 0.05; **: *p* < 0.01; ***: *p* < 0.001.

**
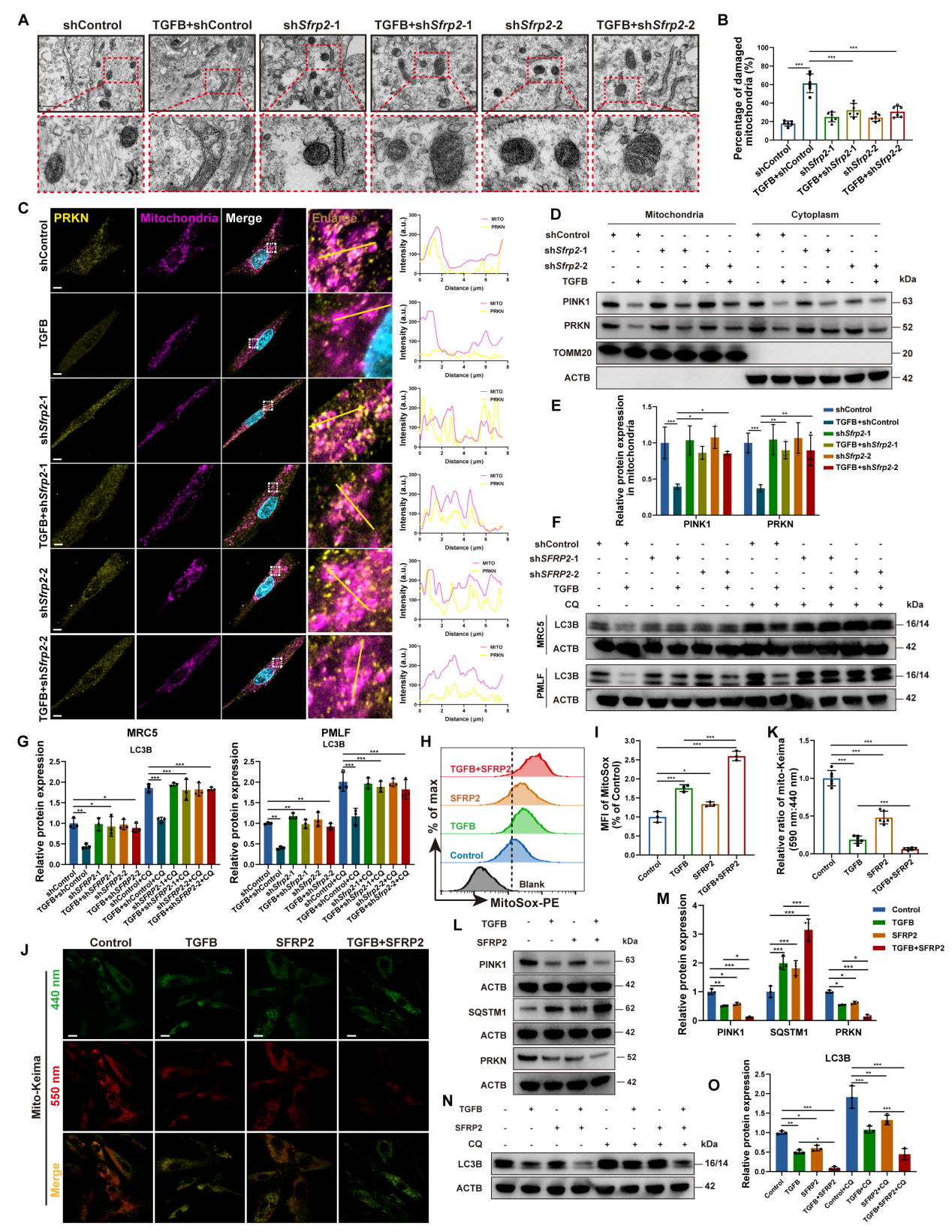
**

**Figure S4.** SFRP2 inhibited PINK1-mediated mitophagy in myofibroblasts. (**A**) Transmission electron microscopy (TEM) images of PMLFs following activation by TGFB/TGF-β and subsequent SFRP2 knockdown. Scale bars: 1 μm. (**B**) Quantitative analysis of the proportion of damaged mitochondria from (**A**) (n = 6 biological repeats per group). (**C**) Representative IF images depicting the colocalization of PRKN and mitochondria (MitoTracker) in PMLFs activated by TGFB/TGF-β and subsequent to SFRP2 knockdown. Scale bars: 10 µm. (**D**) Western blotting analysis of PINK1 and PRKN expression in the mitochondria of PMLFs activated by TGFB/TGF-β and subsequent to SFRP2 knockdown. (**E**) Quantitative analysis of PINK1 and PRKN expression in the mitochondria of PMLFs activated by TGFB/TGF-β and subsequent to SFRP2 knockdown (n = 3 biological repeats per group). (**F**) Western blotting analysis of LC3B expression in MRC5 cells and PMLFs activated by TGFB/TGF-β and subsequent to SFRP2 knockdown, with or without chloroquine (CQ) treatment. (**G**) Quantitative analysis of LC3B expression from (**F**) (n = 3 biological repeats per group). (**H**) Flow cytometry analysis of mitochondrial reactive oxygen species (mtROS) levels in PMLFs treated with TGFB/TGF-β and SFRP2. MtROS were detected using MitoSOX, with fluorescence measured in the PE channel. (**I**) Quantitative analysis of mean fluorescence intensity (MFI) of MitoSOX to assess mtROS levels in PMLFs treated with TGFB/TGF-β and SFRP2 (n = 3 biological repeats per group). (**J**) Representative IF images of PMLFs transfected with mito-Keima lentiviral vectors, treated with TGFB/TGF-β and SFRP2. Fluorescence was detected at 440 nm (neutral pH) or 590 nm (acidic pH) to monitor mitochondrial dynamics. Scale bars: 20 µm. (**K**) Quantitative analysis of the mitophagic flux index, presented as the ratio of 590 nm:440 nm fluorescence signals from (**J**) (n = 6 biological repeats per group). (**L**) Western blotting analysis of PINK1, SQSTM1/p62 and PRKN expression in PMLFs treated with TGFB/TGF-β and SFRP2. (**M**) Quantitative analysis of PINK1, SQSTM1/p62 and PRKN protein levels normalized to ACTB from (**L**) (n = 3 biological repeats per group). (**N**) Western blotting analysis of LC3B expression in PMLFs treated with TGFB/TGF-β and SFRP2, with or without CQ treatment. (**O**) Quantitative analysis of LC3B protein levels normalized to ACTB from (**N**) (n = 3 biological repeats per group). Data are presented as mean±SDs; *: *p* < 0.05; **: *p* < 0.01; ***: *p* < 0.001.

**
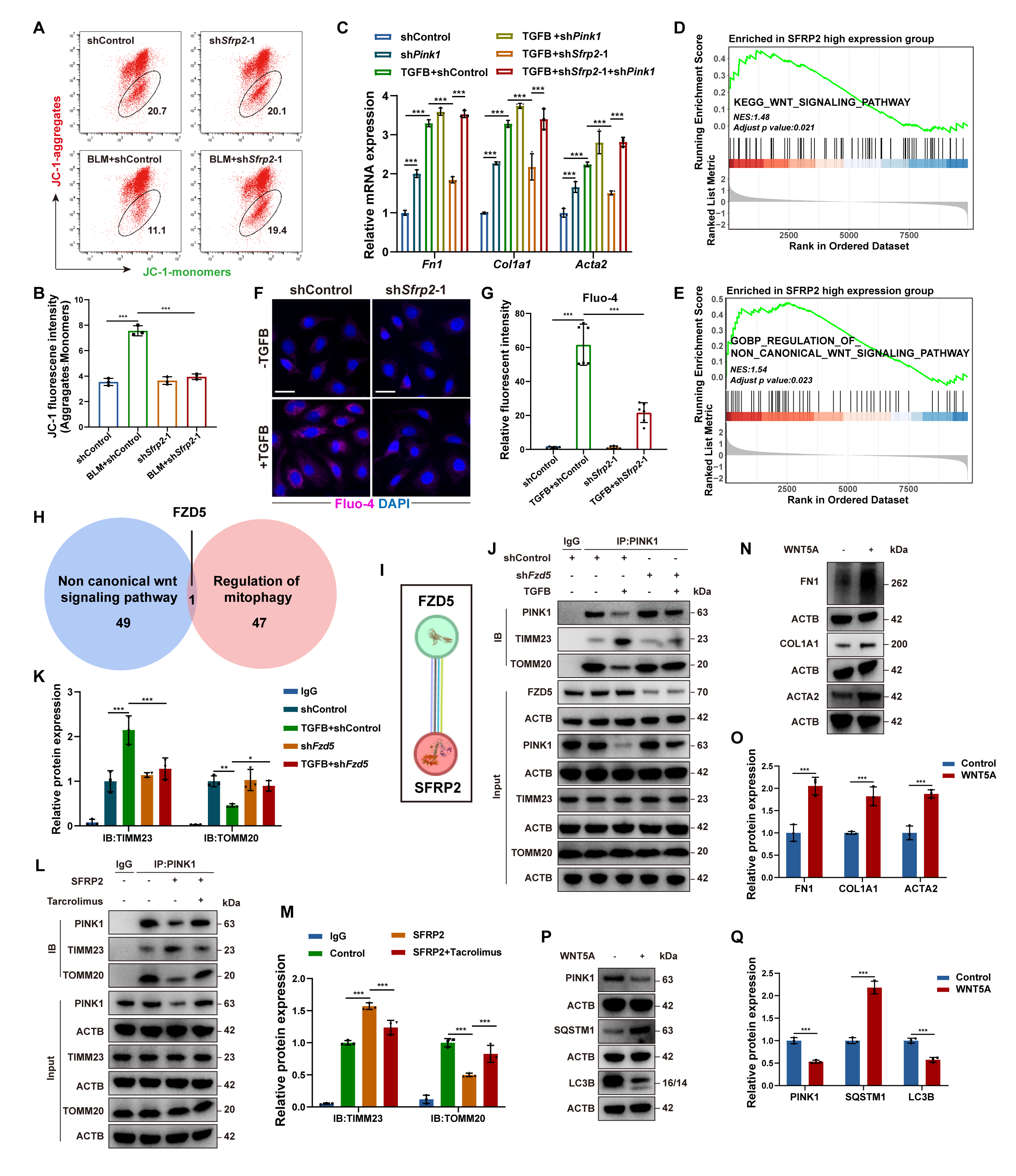
**

**Figure S5.** SFRP2 promoted the translocation of PINK1 into mitochondria via the FZD5-mediated WNT-Ca²⁺ signaling pathway. (**A**) Flow cytometry analysis of mitochondrial membrane potential (∆Ψm) using JC-1 in PMLFs derived from experimental mice treated with BLM or PBS, following SFRP2 knockdown. (**B**) Quantitative analysis of the ratio of JC-1 aggregates (indicative of high ∆Ψm, PE channel) to JC-1 monomers (indicative of low ∆Ψm, FITC channel) from **(A)** (n = 3 biological repeats per group). (**C**) qPCR analysis of *Fn1*, *Col1a1* and *Acta2* expression in PMLFs activated by TGFB/TGF-β and subsequently subjected to SFRP2 and PINK1 knockdown (n = 3 biological repeats per group). (**D**) GSEA of KEGG gene sets revealed a positive correlation between high SFRP2 expression and the WNT signaling pathway. (**E**) GSEA of GO gene sets revealed a positive correlation between high SFRP2 expression and the noncanonical WNT signaling pathway. (**F**) IF staining of Fluo-4 in PMLFs activated by TGFB/TGF-β and following SFRP2 knockdown. Scale bars: 25 µm. (**G**) Quantitative analysis of relative fluorescent intensity from (**F**) (n = 6 biological repeats per group). (**H**) Intersection of gene sets from the GO pathway "Regulation of Mitophagy" (GO:1901524) and the "Noncanonical WNT Signaling Pathway" (GO:0035567) identified FZD5 as the sole common gene. (**I**) STRING analysis depicting the interaction between SFRP2 and FZD5. (**J**) Immunoprecipitation (IP) using an anti-PINK1 antibody in PMLFs activated by TGFB/TGF-β and subsequent to FZD5 knockdown. (**K**) Quantitative analysis of immunoprecipitated TIMM23 and TOMM20 levels from (**J**) (n = 3 biological repeats per group). (**L**) IP using an anti-PINK1 antibody in PMLFs treated with SFRP2 and tacrolimus. (**M**) Quantitative analysis of immunoprecipitated TIMM23 and TOMM20 levels from (**L**) (n = 3 biological repeats per group). (**N**) Western blotting analysis of FN1, COL1A1 and ACTA2 in PMLFs treated with WNT5A. (**O**) Quantitative analysis of FN1, COL1A1 and ACTA2 protein levels normalized to ACTB from (**N**) (n = 3 biological repeats per group). (**P**) Western blotting analysis of PINK1, SQSTM1/p62 and LC3B in PMLFs treated with WNT5A. (**Q**) Quantitative analysis of PINK1, SQSTM1/p62 and LC3B protein levels normalized to ACTB from (**P**) (n = 3 biological repeats per group). Data are presented as mean±SD; *: *p* < 0.05; **: *p* < 0.01; ***: *p* < 0.001.


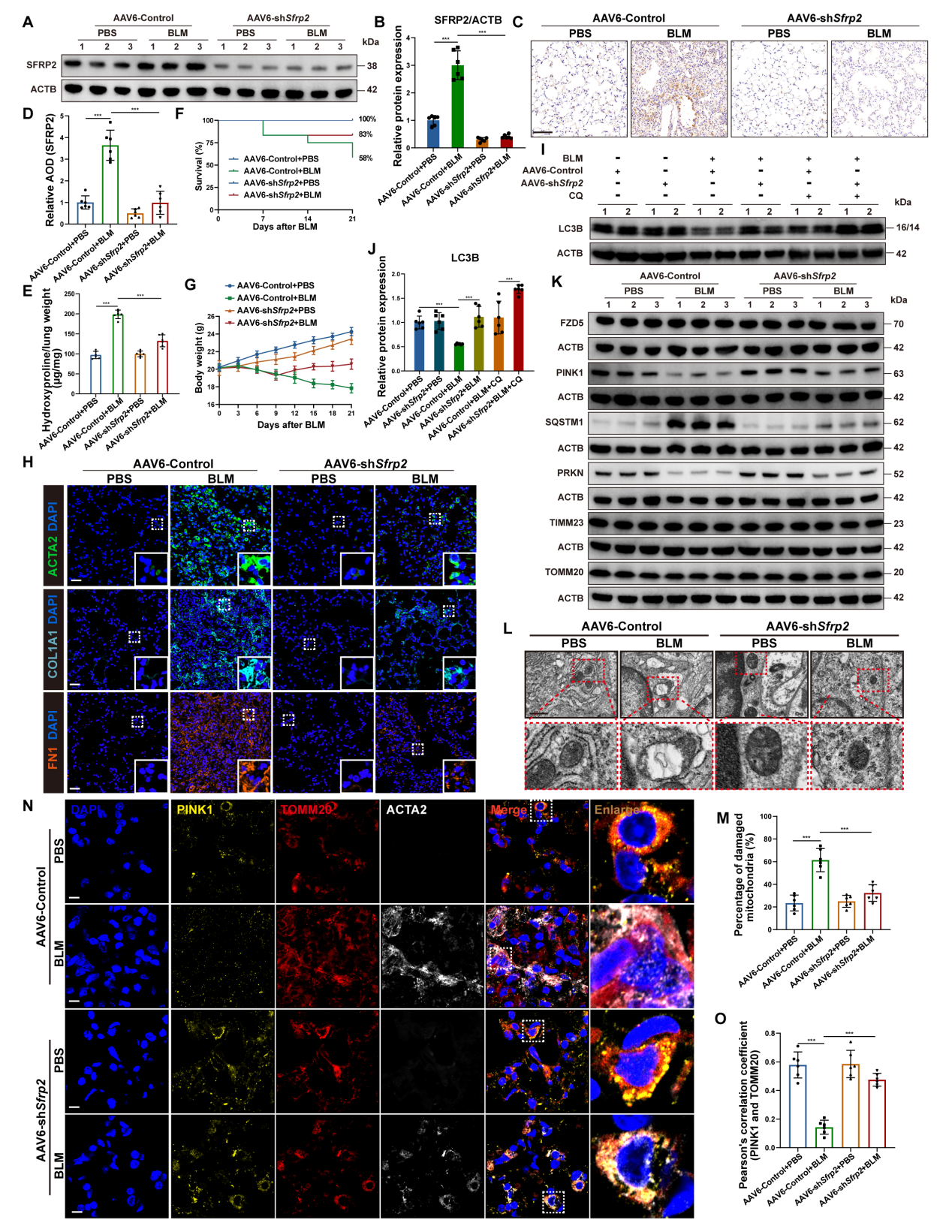


**Figure S6.** Suppression of SFRP2 alleviated pulmonary fibrosis in experimental mouse models. (**A**) Western blotting analysis of SFRP2 protein levels in the lungs of experimental mice treated with AAV6-sh*Sfrp2* or AAV6-Control. (**B**) Quantitative analysis of SFRP2 protein levels normalized to ACTB from (**A**) (n = 6 biological repeats per group). (**C-D**) IHC staining and quantitative analysis of SFRP2 in lung sections from experimental mouse models treated with AAV6-sh*Sfrp2* or AAV6-Control. Scale bars: 100 µm. (n = 6 biological repeats per group). (**E**) Hydroxyproline levels in lungs from experimental mouse models treated with AAV6-sh*Sfrp2* or AAV6-Control (n=6 biological repeats per group). (**F**) Survival rates of experimental mice over a 21-day period (n = 12 mice per group). (**G**) Changes in body weight of experimental mice monitored over a 21-day period (n = 12 mice per group). (**H**) Representative IF images of ACTA2, COL1A1, and FN1 in lung sections from experimental mouse models treated with AAV6-sh*Sfrp2* or AAV6-Control. Scale bars: 50 µm. (**I**) Western blotting analysis of LC3B protein levels in the lungs of experimental mice treated with AAV6-sh*Sfrp2* or AAV6-Control, with or without CQ treatment. (**J**) Quantitative analysis of LC3B protein levels normalized to ACTB from (**I**) (n = 6 biological repeats per group). (**K**) Western blotting analysis of FZD5, PINK1, SQSTM1/p62, PRKN, TIMM23 and TOMM20 protein levels in the lungs of experimental mice treated with AAV6-sh*Sfrp2* or AAV6-Control. (**L**) TEM images of lung sections from experimental mouse models treated with AAV6-sh*Sfrp2* or AAV6-Control. Scale bars: 1 μm. (**M**) Quantitative analysis of the proportion of damaged mitochondria from (**L**) (n = 6 biological repeats per group). (**N**) Representative IF images depicting the colocalization of PINK1 (yellow) and TOMM20 (red) in *ACTA2*^+^ myofibroblasts (white) from the lungs of experimental mice treated with AAV6-sh*Sfrp2* or AAV6-Control. scale bars: 5 µm. (**O**) Pearson correlation coefficient quantifying the colocalization of PINK1 and TOMM20 (n = 6 biological repeats per group). Data are presented as mean±SD; ***: *p* < 0.001.


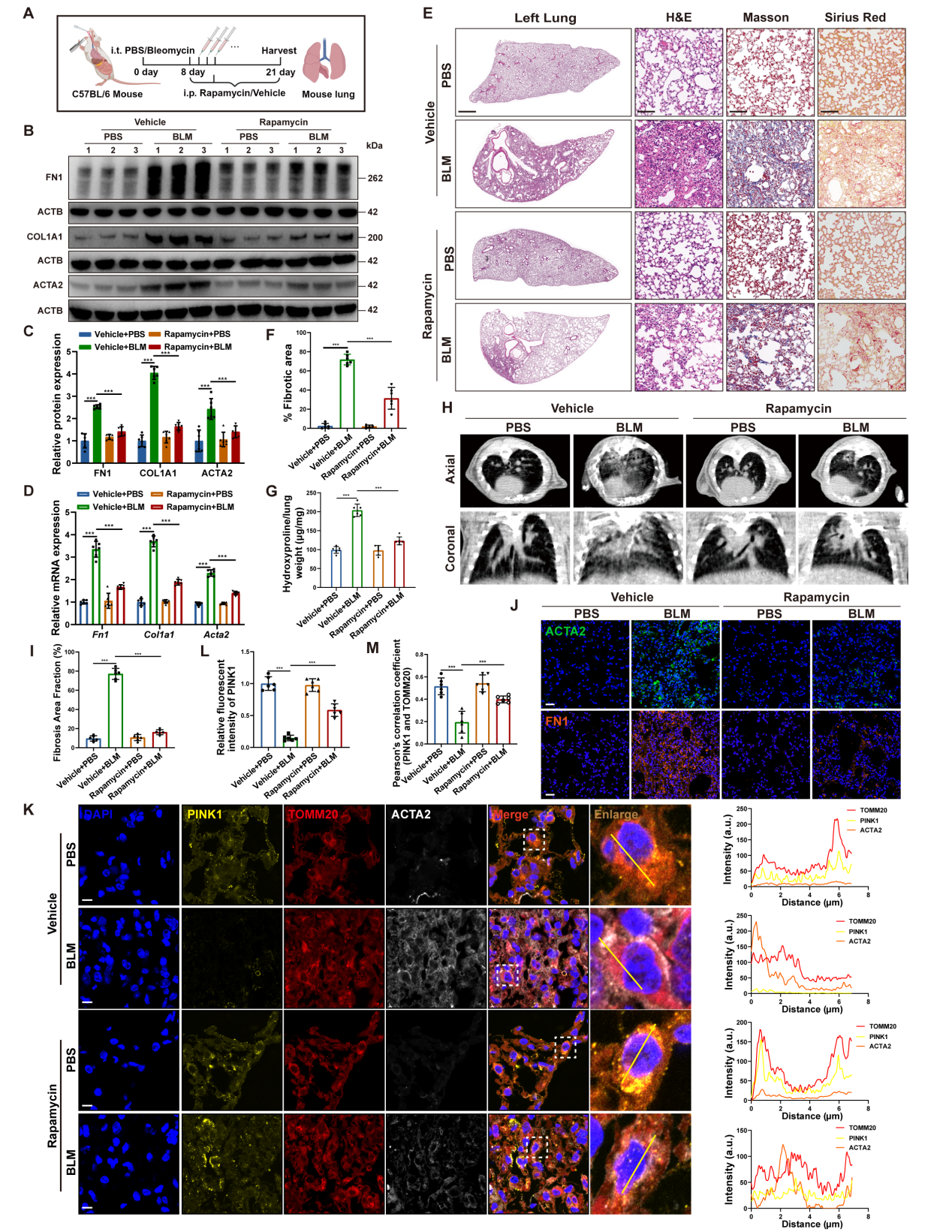


**Figure S7.** Enhancing mitophagy through rapamycin administration alleviated pulmonary fibrosis in experimental mouse models. (**A**) Schematic diagram illustrating the administration of rapamycin in experimental mouse models induced by BLM or PBS. (**B**) Western blotting analysis of FN1, COL1A1 and ACTA2 protein levels in the lungs of experimental mice treated with rapamycin or vehicle. (**C**) Quantitative analysis of protein levels normalized to ACTB from (**B**) (n = 6 biological repeats per group). (**D**) qPCR analysis of *Fn1*, *Col1a1* and *Acta2* mRNA levels in the lungs of experimental mice treated with rapamycin or vehicle (n = 6 biological repeats per group). (**E**) Representative images of H&E staining, Masson’s trichrome staining, and Sirius red staining in lung sections from experimental mice treated with rapamycin or vehicle. Scale bars: 600 µm and 100 µm. (**F**) Quantitative analysis of the fibrotic stroma area in H&E-stained lung sections, as shown in (**E**) (n = 6 biological repeats per group). (**G**) Hydroxyproline levels in lung sections from experimental mouse models treated with rapamycin or vehicle (n=6 biological repeats per group). (**H**) Micro-CT images in axial and coronal views depicting the lungs of experimental mice treated with rapamycin or vehicle. (**I**) Quantitative analysis of fibrosis area fraction from (**H**) (n = 6 biological repeats per group). (**J**) Representative IF images of ACTA2 and FN1 in lung sections from experimental mice treated with rapamycin or vehicle. Scale bars: 50 µm. (**K**) Representative IF images depicting the colocalization of PINK1 (yellow) and TOMM20 (red) in *ACTA2*^+^ myofibroblasts (white) from the lungs of experimental mice treated with rapamycin or vehicle. scale bars: 5 µm. (**L**) Quantitative analysis of relative fluorescent intensity from (**K**) (n = 6 biological repeats per group). (**M**) Pearson correlation coefficient quantifying the colocalization of PINK1 and TOMM20 from (**K**) (n = 6 biological repeats per group). Data are presented as mean ± SD; ***: *p* < 0.001.


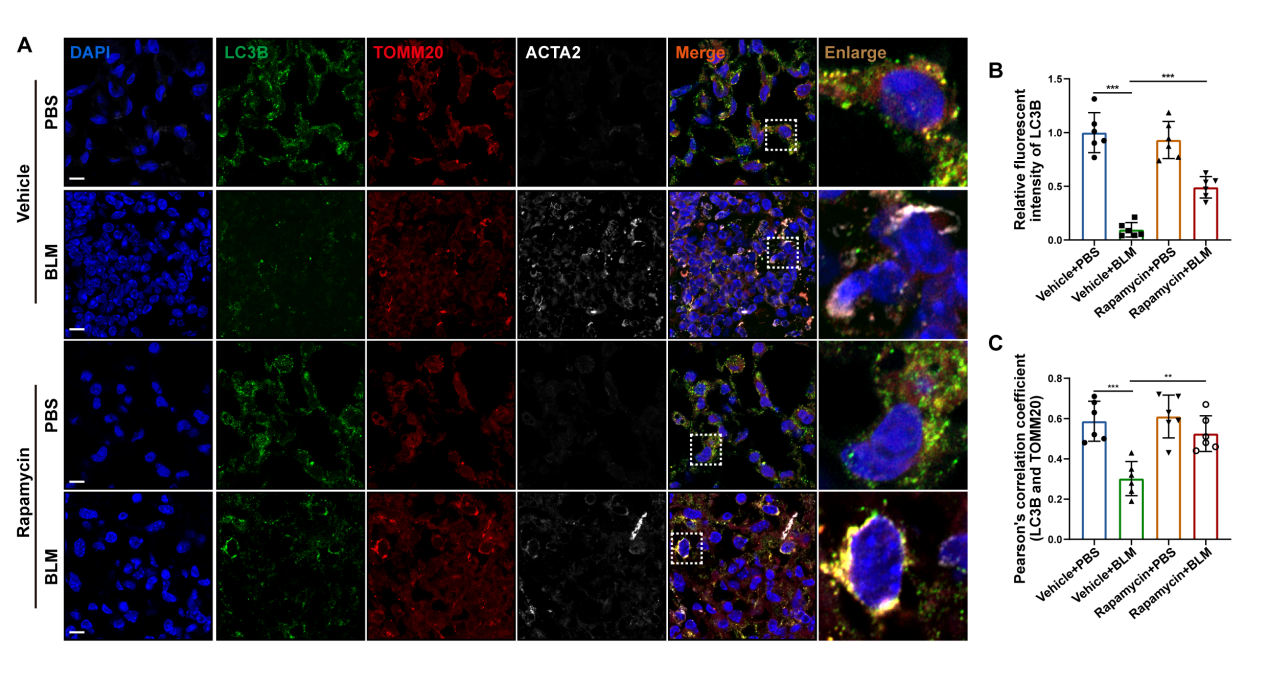


**Figure S8.** Enhancing mitophagy through rapamycin administration alleviated pulmonary fibrosis in experimental mouse models. (**A**) Representative IF images depicting the colocalization of LC3B (green) and TOMM20 (red) in *ACTA2*^+^ myofibroblasts (white) from the lungs of experimental mice treated with rapamycin or vehicle. scale bars: 5 µm. (**B**) Quantitative analysis of relative fluorescent intensity from (**A**) (n = 6 biological repeats per group). (**C**) Pearson correlation coefficient quantifying the colocalization of LC3B and TOMM20 from (**A**) (n = 6 biological repeats per group). Data are presented as mean ± SD; **: *p* < 0.01.***: *p* < 0.001.

|  |
| --- |
