# Supplementary material for: Autocrine SFRP2 (secreted frizzled related protein 2) enhances lung myofibroblast fibrogenic activity by suppressing PINK1-mediated mitophagy initiation
Source: Autophagy. 2026 Mar 15;22(6):1333–50. doi: 10.1080/15548627.2026.2642341 (PMC13185440; doi:10.1080/15548627.2026.2642341)
Supplement: Supplementary_Data R4.docx [file KAUP_A_2642341_SM1897.docx]

**Data S1**. DEGs between IPF and healthy donor-derived lung myofibroblasts.

| gene | | p_val | avg_log2FC | p_val_adj | cluster |
| --- | --- | --- | --- | --- | --- |
| *COL1A1* | | 0 | 3.53081285296069 | 0 | IPF |
| *SFTPC* | | 0 | 3.42587932094686 | 0 | Donor |
| *COL3A1* | | 0 | 3.28995292666647 | 0 | IPF |
| *POSTN* | | 5.98847003219004e-217 | 3.05174868973036 | 1.26961553152461e-212 | IPF |
| *SFRP2* | | 7.78720858703201e-253 | 3.00962325920123 | 1.65096609253666e-248 | IPF |
| *GPX3* | | 0 | 2.62570844365685 | 0 | Donor |
| *IGFBP4* | | 0 | 2.55946456076271 | 0 | IPF |
| *IGKC* | | 1.09790948897842e-159 | 2.5571074280855 | 2.32767790758314e-155 | IPF |
| *COL1A2* | | 0 | 2.50927893326013 | 0 | IPF |
| *CYP4B1* | | 0 | 2.3767142724363 | 0 | Donor |
| *SPARC* | | 0 | 2.31064375717892 | 0 | IPF |
| *CTHRC1* | | 1.56652090118065e-173 | 2.29983276924789 | 3.3211809625931e-169 | IPF |
| *THY1* | | 0 | 2.15352370073998 | 0 | IPF |
| *SFRP4* | | 1.61889618512913e-92 | 1.97507461860132 | 3.43222180209227e-88 | IPF |
| *ASPN* | | 1.26086486336185e-123 | 1.91347307017894 | 2.67315959681347e-119 | IPF |
| *BGN* | | 3.74450037210558e-271 | 1.88972011391585 | 7.93871523890104e-267 | IPF |
| *WISP2* | | 1.37294024147378e-166 | 1.88653440519935 | 2.91077060594856e-162 | Donor |
| *FMO2* | | 4.14668889337749e-222 | 1.85454798863936 | 8.79139512284962e-218 | Donor |
| *COL6A3* | | 7.38532881261563e-299 | 1.85178608024787 | 1.56576356156264e-294 | IPF |
| *CXCL14* | | 5.20474837726619e-73 | 1.79184402614547 | 1.1034587034642e-68 | IPF |
| *PTGDS* | | 1.47966994032589e-168 | 1.77209155069638 | 3.13704824048491e-164 | IPF |
| *CA3* | | 2.33364863890348e-85 | 1.73893078499097 | 4.94756847933927e-81 | Donor |
| *PRELP* | | 9.88910446359451e-140 | 1.71274882332149 | 2.09658903732667e-135 | Donor |
| *IL6* | | 1.18357614126266e-32 | 1.69357264796865 | 2.50929977709097e-28 | Donor |
| *IGF1* | | 6.78079155975351e-229 | 1.68143547496125 | 1.43759561858334e-224 | IPF |
| *APOD* | | 7.01950199963086e-59 | 1.66671971143876 | 1.48820461894174e-54 | Donor |
| *COMP* | | 1.26561563166731e-101 | 1.66461020751451 | 2.68323170069786e-97 | IPF |
| *COL14A1* | | 2.85910394982153e-253 | 1.65839043204939 | 6.06158628401662e-249 | IPF |
| *TPPP3* | | 5.15502333975646e-209 | 1.63351078860303 | 1.09291649826177e-204 | IPF |
| *SCN7A* | | 6.90331745266362e-70 | 1.63308455158747 | 1.46357233313921e-65 | Donor |
| *PPP1R14A* | | 1.62208853030246e-101 | 1.59063202565428 | 3.43898989309424e-97 | Donor |
| *CCDC80* | | 8.22048042068155e-230 | 1.55691592076682 | 1.7428240539887e-225 | IPF |
| *FIGF* | | 4.99489434096184e-144 | 1.54957991116489 | 1.05896754922732e-139 | Donor |
| *IGHA1* | | 5.46867159506197e-76 | 1.52452795476591 | 1.15941306486909e-71 | IPF |
| *CXCL2* | | 1.47987634326631e-10 | 1.50244297392288 | 3.1374858353589e-06 | Donor |
| *FIBIN* | | 1.2431314073873e-125 | 1.47261260214523 | 2.63556289680182e-121 | Donor |
| *MDK* | | 3.8964968262992e-210 | 1.46104004471407 | 8.26096292143692e-206 | IPF |
| *TIMP1* | | 9.21442591374299e-212 | 1.43767149147066 | 1.95355043797265e-207 | IPF |
| *RGCC* | | 1.39314464843023e-90 | 1.43521047053072 | 2.95360596913692e-86 | Donor |
| *VCAN* | | 5.24914368803153e-228 | 1.43103882732984 | 1.11287095329957e-223 | IPF |
| *LMCD1* | | 1.07469171778552e-79 | 1.42501192301041 | 2.27845391087708e-75 | Donor |
| *GPM6B* | | 1.2666714598825e-87 | 1.38253525635035 | 2.6854701620969e-83 | Donor |
| *FGFR4* | | 1.99353761912361e-104 | 1.38156254854619 | 4.22649910630396e-100 | Donor |
| *GADD45B* | | 1.09643900137506e-64 | 1.3778038269995 | 2.32456032681526e-60 | Donor |
| *CTSK* | | 3.36960346749273e-218 | 1.36436329043707 | 7.14389631143133e-214 | IPF |
| *CD14* | | 1.83019086438868e-105 | 1.34745587327176 | 3.88018765159043e-101 | Donor |
| *CRABP2* | | 4.10493325141859e-143 | 1.32749659919269 | 8.70286898633255e-139 | IPF |
| *LTBP1* | | 2.68261344676465e-221 | 1.31745791313585 | 5.68740876848572e-217 | IPF |
| *ADH1B* | | 2.14275600757699e-198 | 1.31214729490495 | 4.54285701166397e-194 | Donor |
| *MMP2* | | 3.29812880725053e-221 | 1.31057525961469 | 6.99236288425184e-217 | IPF |
| *AOC3* | | 1.50867952910499e-49 | 1.30636053667741 | 3.19855146965549e-45 | Donor |
| *ALDH1A1* | | 1.28619120734315e-92 | 1.30576409019798 | 2.72685397868822e-88 | Donor |
| *SUN2* | | 1.13984405046686e-100 | 1.28139292393277 | 2.41658337139479e-96 | Donor |
| *NDRG2* | | 5.00217245194836e-66 | 1.2590003198231 | 1.06051058153757e-61 | Donor |
| *PPIC* | | 1.57782200386293e-224 | 1.25777472672648 | 3.3451404303898e-220 | IPF |
| *USP53* | | 2.37280079588475e-73 | 1.24039736822275 | 5.03057496735527e-69 | Donor |
| *FBLN5* | | 3.53830249283636e-86 | 1.24012906009456 | 7.50155511506237e-82 | Donor |
| *MACF1* | | 6.8886760681365e-32 | 1.22273247999327 | 1.46046821320562e-27 | Donor |
| *ADM* | | 2.90753398779293e-40 | 1.2173148603214 | 6.16426280751979e-36 | Donor |
| *LBH* | | 1.21006948776906e-40 | 1.21591003137412 | 2.56546832101918e-36 | Donor |
| *COL8A1* | | 2.53372933745499e-123 | 1.19982171141552 | 5.37175956833833e-119 | IPF |
| *CLEC11A* | | 3.23632264815815e-215 | 1.19913581691665 | 6.86132764636009e-211 | IPF |
| *COL5A2* | | 6.44107869212919e-173 | 1.19871783517509 | 1.36557309351831e-168 | IPF |
| *SPINT2* | | 2.03439421282654e-52 | 1.19672049545523 | 4.31311917061355e-48 | Donor |
| *SCGB3A1* | | 4.17563692230664e-146 | 1.16502240174726 | 8.85276783898232e-142 | IPF |
| *SERPINF1* | | 2.13020863649602e-239 | 1.15880373115796 | 4.5162553302352e-235 | IPF |
| *PLAC9* | | 3.28885233929831e-173 | 1.15482463506132 | 6.97269584454635e-169 | IPF |
| *ICAM1* | | 1.32069588690629e-10 | 1.15159865061894 | 2.80000734983002e-06 | Donor |
| *OGN* | | 3.41676226741626e-137 | 1.14829996164337 | 7.2438776831492e-133 | IPF |
| *COL5A1* | | 7.53823687191984e-164 | 1.14010680177867 | 1.59818159921573e-159 | IPF |
| *SERPINE1* | | 5.50077426063625e-102 | 1.13926335868936 | 1.16621915099749e-97 | IPF |
| *MAOA* | | 4.07387059293218e-97 | 1.12814281698674 | 8.63701304407551e-93 | Donor |
| *CTGF* | | 6.01916505737656e-54 | 1.12715991861277 | 1.2761231838144e-49 | Donor |
| *LMNA* | | 2.88135647046617e-128 | 1.1040109888968 | 6.10876385303532e-124 | IPF |
| *SLIT2* | | 3.56661082824199e-36 | 1.09755005930007 | 7.56157161695584e-32 | Donor |
| *DPT* | | 1.99252748740217e-61 | 1.09530350346111 | 4.22435752604135e-57 | IPF |
| *CITED2* | | 1.45347615491504e-52 | 1.09352101432392 | 3.08151479603538e-48 | Donor |
| *CXCL12* | | 1.05924722530266e-134 | 1.09048474336674 | 2.24571004236416e-130 | IPF |
| *MEG3* | | 1.87794965202058e-151 | 1.0599473307259 | 3.98144105724882e-147 | IPF |
| *FN1* | | 7.81027791717552e-100 | 1.05255049409952 | 1.65585702122038e-95 | IPF |
| *TNFSF13B* | | 6.97634280614444e-114 | 1.04923919864982 | 1.47905443833068e-109 | IPF |
| *MOXD1* | | 2.29602087876174e-137 | 1.02980844558186 | 4.86779386506276e-133 | IPF |
| *MFAP2* | | 7.64721340017667e-148 | 1.02692627951093 | 1.62128571297146e-143 | IPF |
| *LTBP2* | | 1.14660340829602e-125 | 1.02322145690464 | 2.43091388592839e-121 | IPF |
| *CRYAB* | | 1.45173539777678e-35 | 1.01918359527529 | 3.07782421682656e-31 | Donor |
| *ADIRF* | | 4.27826216835413e-70 | 1.0084619660309 | 9.07034362312758e-66 | Donor |
| *STEAP1* | | 5.56066088841076e-149 | 0.992930013875841 | 1.17891571495196e-144 | IPF |
| *CYR61* | | 1.75713199811044e-45 | 0.992561697023803 | 3.72529554919395e-41 | Donor |
| *MXRA5* | | 3.3765507464338e-140 | 0.984378129856833 | 7.15862523751429e-136 | IPF |
| *IGFBP3* | | 2.10842859484746e-66 | 0.982946805530551 | 4.47007946393611e-62 | IPF |
| *TDO2* | | 4.48693678798216e-117 | 0.977930243774428 | 9.51275468420098e-113 | IPF |
| *S100A10* | | 4.72710060542522e-147 | 0.97708906120845 | 1.0021925993562e-142 | IPF |
| *SOCS3* | | 2.56011940595242e-46 | 0.973717862761882 | 5.42770915255973e-42 | Donor |
| *LUM* | | 4.2763070143183e-155 | 0.971377717351367 | 9.06619850105624e-151 | IPF |
| *A2M* | | 2.45658495039477e-71 | 0.960273559286215 | 5.20820575333195e-67 | IPF |
| *TUBA1A* | | 9.96832165277046e-117 | 0.959590047516993 | 2.11338387360386e-112 | IPF |
| *PHLDA1* | | 3.80437608929046e-123 | 0.941770048277885 | 8.0656577469047e-119 | IPF |
| *CSRP2* | | 5.5455997916364e-31 | 0.9394199555185 | 1.17572261182483e-26 | Donor |
| *INHBA* | | 2.15397574426593e-81 | 0.935860515376469 | 4.56664397541821e-77 | IPF |
| *FBP1* | | 2.09811323194946e-58 | 0.931354462364491 | 4.44820986305606e-54 | Donor |
| *TCEAL4* | | 7.17798938058082e-39 | 0.921040117093513 | 1.52180552857694e-34 | Donor |
| *MGP* | | 8.06983404756421e-85 | 0.920275441584656 | 1.71088551642409e-80 | IPF |
| *CKB* | | 6.76152127565878e-52 | 0.915336445477082 | 1.43351012565242e-47 | Donor |
| *PLEKHH2* | | 1.48182952923332e-14 | 0.913392508668594 | 3.14162678492756e-10 | Donor |
| *MAMDC2* | | 4.14120991777439e-32 | 0.911046467590202 | 8.7797791466735e-28 | Donor |
| *CRIP2* | | 7.20938464965227e-146 | 0.910701155683221 | 1.52846163957278e-141 | IPF |
| *SLPI* | | 2.50783125841579e-07 | 0.910530259893942 | 0.00531685305096731 | Donor |
| *IER2* | | 2.33372585962765e-36 | 0.90505686841442 | 4.94773219499658e-32 | Donor |
| *ITIH5* | | 1.68350528057726e-128 | 0.903975753874846 | 3.56919954535185e-124 | IPF |
| *TSHZ2* | | 3.33720091896372e-150 | 0.903344090188643 | 7.07519966829497e-146 | IPF |
| *FHL2* | | 5.46799744863217e-121 | 0.900927655218624 | 1.15927013908451e-116 | IPF |
| *DCXR* | | 2.69980615090373e-43 | 0.89978494949547 | 5.72385902053099e-39 | Donor |
| *RCN3* | | 8.64096436410748e-139 | 0.877608360299815 | 1.83197085483443e-134 | IPF |
| *ANTXR1* | | 3.66506904575402e-149 | 0.877446745879412 | 7.77031288390309e-145 | IPF |
| *GPC3* | | 1.52935527805206e-58 | 0.87551417823875 | 3.24238612499817e-54 | Donor |
| *IGLC2* | | 8.93414709820291e-86 | 0.870035065445373 | 1.89412852629e-81 | IPF |
| *FBLN2* | | 1.93861998720197e-120 | 0.867962697253965 | 4.11006823486691e-116 | IPF |
| *NBL1* | | 1.53150291891515e-133 | 0.867887137616264 | 3.24693933839201e-129 | IPF |
| *SVEP1* | | 9.62943043675388e-36 | 0.855020945772537 | 2.04153554689619e-31 | Donor |
| *ISLR* | | 2.7046380986477e-83 | 0.853763954893215 | 5.73410323294298e-79 | IPF |
| *APOE* | | 3.06908789451225e-62 | 0.852775091291941 | 6.50677324515541e-58 | IPF |
| *IRF1* | | 3.65384473466083e-20 | 0.84776601545917 | 7.74651622195443e-16 | Donor |
| *EMP1* | | 8.10397349751114e-71 | 0.847132183078738 | 1.71812342120734e-66 | IPF |
| *SERPING1* | | 4.00699872714202e-99 | 0.846082904980165 | 8.49523800141381e-95 | Donor |
| *LXN* | | 5.2188143245809e-92 | 0.837292867552582 | 1.1064408249544e-87 | IPF |
| *THBS1* | | 4.64185486344218e-22 | 0.830951714027225 | 9.84119649598376e-18 | Donor |
| *CCBE1* | | 8.92599068371594e-42 | 0.829971638388788 | 1.89239928485462e-37 | Donor |
| *LOXL1* | | 4.17971256866894e-111 | 0.825538125422734 | 8.86140861683502e-107 | IPF |
| *LRRC17* | | 6.53023320416068e-107 | 0.823337000064719 | 1.38447474161411e-102 | IPF |
| *CERCAM* | | 2.30775436544405e-126 | 0.823109728132164 | 4.89267003017792e-122 | IPF |
| *FOSB* | | 1.17295526896493e-93 | 0.81848160697062 | 2.48678246573255e-89 | IPF |
| *VIM* | | 6.77959435396759e-208 | 0.803718980427785 | 1.43734179898467e-203 | IPF |
| *GLUL* | | 1.43206906212595e-61 | 0.800163709473409 | 3.03612961861322e-57 | Donor |
| *NFKBIZ* | | 1.66076800028237e-27 | 0.800133184020222 | 3.52099423739866e-23 | Donor |
| *NFKBIA* | | 1.30476666008435e-17 | 0.797599789000214 | 2.76623579604483e-13 | Donor |
| *SOD3* | | 2.83245771087642e-49 | 0.796297614699824 | 6.00509359282909e-45 | Donor |
| *HTRA1* | | 1.99756175698569e-96 | 0.79570938129386 | 4.23503068098536e-92 | IPF |
| *MFGE8* | | 1.74371548622254e-27 | 0.793275851344287 | 3.69685120234041e-23 | Donor |
| *NEDD9* | | 8.00436920639348e-22 | 0.790001513746341 | 1.69700631544748e-17 | Donor |
| *PCOLCE* | | 3.76998782219596e-114 | 0.788691627722465 | 7.99275118183765e-110 | IPF |
| *CFD* | | 5.27157373275976e-90 | 0.788427620298735 | 1.1176263470824e-85 | Donor |
| *SERPINE2* | | 1.53588330676991e-96 | 0.78410660236369 | 3.25622619868289e-92 | IPF |
| *CD99* | | 6.24985172825962e-106 | 0.772507105930378 | 1.32503106490832e-101 | IPF |
| *ECM1* | | 1.50001775088867e-117 | 0.769640075108738 | 3.18018763365908e-113 | IPF |
| *EID1* | | 1.80089502017971e-128 | 0.76808181086611 | 3.818077532283e-124 | IPF |
| *CFH* | | 5.95738140021829e-75 | 0.766890845765687 | 1.26302443066028e-70 | IPF |
| *PTN* | | 2.21574127038725e-88 | 0.762199931805497 | 4.69759306734801e-84 | IPF |
| *PDK4* | | 1.86630320213514e-33 | 0.761296167093624 | 3.95674941884672e-29 | Donor |
| *PDLIM3* | | 7.60390180521944e-107 | 0.761143722490549 | 1.61210322172457e-102 | IPF |
| *ITM2C* | | 1.65471198966578e-115 | 0.760523098588938 | 3.50815488929042e-111 | IPF |
| *IRS2* | | 2.29824809480611e-24 | 0.760071573624475 | 4.87251578579843e-20 | Donor |
| *COL15A1* | | 1.83911837261683e-113 | 0.759991820987115 | 3.89911486178495e-109 | IPF |
| *CHPT1* | | 1.02502088346573e-16 | 0.7562829114352 | 2.1731467750357e-12 | Donor |
| *CD9* | | 7.64926764051446e-82 | 0.746892300599432 | 1.62172123246547e-77 | IPF |
| *CHN1* | | 4.92619855407339e-108 | 0.744182436595153 | 1.0444033554491e-103 | IPF |
| *COL6A1* | | 6.07979274755932e-86 | 0.740911438387954 | 1.28897686041005e-81 | IPF |
| *HIF3A* | | 8.42936705147224e-47 | 0.740304254534017 | 1.78711010858263e-42 | Donor |
| *THBS2* | | 3.14804980581748e-92 | 0.737583693104392 | 6.67418039331363e-88 | IPF |
| *DPYSL3* | | 6.21539409928733e-116 | 0.732892553775778 | 1.31772570298991e-111 | IPF |
| *PPM1K* | | 3.83462824899539e-22 | 0.732369449495486 | 8.12979535069512e-18 | Donor |
| *MAP3K8* | | 2.79308325139335e-23 | 0.728598196513897 | 5.92161580127905e-19 | Donor |
| *PDLIM4* | | 4.64609788744464e-97 | 0.719179921991217 | 9.85019213117137e-93 | IPF |
| *C10orf54* | | 2.82196694125895e-18 | 0.718844070670959 | 5.98285211216309e-14 | Donor |
| *TNC* | | 8.03800558505955e-57 | 0.711165010601336 | 1.70413756408848e-52 | IPF |
| *PDLIM7* | | 1.82350442167617e-112 | 0.707944203920566 | 3.86601172439564e-108 | IPF |
| *PIK3R1* | | 2.52693661802171e-11 | 0.706373532884793 | 5.35735832386783e-07 | Donor |
| *COL6A2* | | 3.54804362485373e-99 | 0.706251149689589 | 7.5222072890524e-95 | IPF |
| *NABP1* | | 2.29879300767914e-18 | 0.704110404313618 | 4.87367105558055e-14 | Donor |
| *SERPINH1* | | 1.203443087903e-85 | 0.70406861475394 | 2.55141969066315e-81 | IPF |
| *SDPR* | | 3.2411478709406e-25 | 0.703103391493966 | 6.87155760118117e-21 | Donor |
| *PTGIR* | | 1.7719514119452e-17 | 0.702340720271257 | 3.75671418846502e-13 | Donor |
| *CYB5A* | | 8.10951631827643e-21 | 0.695664463617622 | 1.71929855463779e-16 | Donor |
| *SLC1A5* | | 1.87076454942711e-24 | 0.694933267967878 | 3.96620792124043e-20 | Donor |
| *MAP1B* | | 1.28476653199477e-92 | 0.694074948907502 | 2.72383352448212e-88 | IPF |
| *COL18A1* | | 8.70707244147983e-109 | 0.692261783160726 | 1.84598642831814e-104 | IPF |
| *CSRP1* | | 2.56402607357234e-09 | 0.692103135183541 | 5.43599167858071e-05 | Donor |
| *GPNMB* | | 5.12521884593001e-78 | 0.691542400852537 | 1.08659764752562e-73 | IPF |
| *ARPC1B* | | 1.74511620077828e-107 | 0.684533151220066 | 3.69982085727002e-103 | IPF |
| *MPST* | | 7.06132596743025e-17 | 0.681838275423809 | 1.49707171835489e-12 | Donor |
| *QSOX1* | | 8.24373158775888e-22 | 0.679619893984324 | 1.74775353392076e-17 | Donor |
| *ALDH1A3* | | 6.1550129950703e-80 | 0.679473904470016 | 1.30492430508485e-75 | IPF |
| *NEAT1* | | 1.84318168923368e-78 | 0.679353400644654 | 3.90772949934432e-74 | IPF |
| *IGFBP7* | | 3.102232996757e-96 | 0.671954879243596 | 6.57704417642451e-92 | IPF |
| *CD63* | | 1.5546007324166e-167 | 0.66981539279671 | 3.29590901279644e-163 | IPF |
| *NGFRAP1* | | 1.11313360105698e-104 | 0.669320201003966 | 2.3599545476009e-100 | IPF |
| *EFEMP2* | | 3.13129427018272e-111 | 0.66855654998813 | 6.63865698221439e-107 | IPF |
| *RHOB* | | 3.19973372782229e-25 | 0.667541524033185 | 6.78375547635604e-21 | Donor |
| *HLA-A* | | 1.34328787722572e-112 | 0.665996217778729 | 2.84790462850624e-108 | IPF |
| *CD81* | | 1.1594935713398e-100 | 0.664592689505766 | 2.4582423205975e-96 | IPF |
| *PAPPA* | | 2.69596939701693e-68 | 0.663240759304237 | 5.7157247186156e-64 | IPF |
| *MAFF* | | 2.0628461897043e-12 | 0.662780117207845 | 4.37344020679209e-08 | Donor |
| *CD302* | | 1.13094676581677e-15 | 0.662375948803012 | 2.39772023820813e-11 | Donor |
| *SSPN* | | 1.0282801749354e-85 | 0.65621579397882 | 2.18005679888054e-81 | IPF |
| *PTPRS* | | 2.98069515674157e-05 | 0.655744127390441 | 0.631937180180781 | Donor |
| *PLTP* | | 1.35794061961152e-79 | 0.654826328703228 | 2.87896990763839e-75 | IPF |
| *HLA-E* | | 7.3602523873459e-25 | 0.649827384716831 | 1.5604471086412e-20 | Donor |
| *FAP* | | 5.01649495514267e-102 | 0.648219922487306 | 1.0635470954398e-97 | IPF |
| *SLC40A1* | | 4.29954163539547e-08 | 0.645770043923753 | 0.000911545822120194 | Donor |
| *HMGN1* | | 1.03936347470264e-107 | 0.642474859136795 | 2.20355450271707e-103 | IPF |
| *TAGLN* | | 5.82215536599083e-50 | 0.640527288152965 | 1.23435515914372e-45 | IPF |
| *SSC5D* | | 2.06128859690534e-104 | 0.639398082141508 | 4.37013795429902e-100 | IPF |
| *ENTPD1* | | 6.13261715983735e-101 | 0.639311502596121 | 1.30017616405712e-96 | IPF |
| *IL32* | | 1.67071332502257e-109 | 0.638332310174303 | 3.54207932038035e-105 | IPF |
| *MFAP4* | | 3.55662999854781e-60 | 0.633150020393605 | 7.54041125992121e-56 | IPF |
| *SEPW1* | | 1.64231033921976e-100 | 0.632945119219983 | 3.48186215017982e-96 | IPF |
| *RARRES3* | | 4.27982487996122e-88 | 0.632648701782997 | 9.07365672800578e-84 | IPF |
| *PPDPF* | | 7.98849886919896e-19 | 0.62877050269925 | 1.69364164525887e-14 | Donor |
| *TTC3* | | 9.78234262997075e-95 | 0.62866572821141 | 2.0739544609801e-90 | IPF |
| *ANK2* | | 3.61570863957874e-90 | 0.626648872809585 | 7.66566388677088e-86 | IPF |
| *HMGB2* | | 8.07597373210217e-16 | 0.626541592981079 | 1.71218719094298e-11 | Donor |
| *ZMAT3* | | 1.38649575472762e-94 | 0.626500065738422 | 2.93950964959802e-90 | IPF |
| *ACTA2* | | 1.68629540408446e-49 | 0.624964024637931 | 3.57511488619945e-45 | IPF |
| *GLT8D2* | | 1.84553580415289e-104 | 0.622647708312798 | 3.91272045838453e-100 | IPF |
| *ABL2* | | 2.61134043718899e-61 | 0.62145299655929 | 5.53630286088438e-57 | IPF |
| *LY6E* | | 2.43650681423027e-85 | 0.619452868589618 | 5.1656380968496e-81 | IPF |
| *TIMP2* | | 8.74880166711625e-101 | 0.619301303742086 | 1.85483344144532e-96 | IPF |
| *PLPP1* | | 2.36180295058413e-70 | 0.617616487428993 | 5.00725843553342e-66 | IPF |
| *METTL7A* | | 1.20187948099902e-18 | 0.616963925858592 | 2.54810468766602e-14 | Donor |
| *RAB31* | | 1.47489794798048e-101 | 0.614213050375187 | 3.12693113951341e-97 | IPF |
| *APP* | | 2.8710222112164e-82 | 0.612676507335869 | 6.0868541899999e-78 | IPF |
| *ANXA2* | | 4.26216714664392e-101 | 0.608492724839435 | 9.03622056759977e-97 | IPF |
| *MRC2* | | 1.64052253908372e-109 | 0.605852523193474 | 3.47807183511139e-105 | IPF |
| *MYL12A* | | 3.03021515943489e-30 | 0.604141878655649 | 6.42435915951792e-26 | Donor |
| *GABPB1-AS1* | | 6.20418264666066e-10 | 0.603569000119371 | 1.31534876291853e-05 | Donor |
| *PMEPA1* | | 1.13252726637773e-72 | 0.602305779665335 | 2.40107105744743e-68 | IPF |
| *CFI* | | 1.08672513600945e-75 | 0.601599756214697 | 2.30396596085364e-71 | IPF |
| *LEPR* | | 6.13424474726739e-28 | 0.600470515531373 | 1.30052122886816e-23 | IPF |
| *ZFP36L1* | | 2.95567861269381e-55 | 0.595253793648549 | 6.26633422677214e-51 | IPF |
| *NAMPT* | | 1.00689365321768e-06 | 0.591680924319483 | 0.021347152341868 | Donor |
| *MMP19* | | 2.96879389930544e-73 | 0.587819105028404 | 6.29413994591747e-69 | IPF |
| *SPON2* | | 5.70971823039305e-44 | 0.585593146193905 | 1.21051736202563e-39 | IPF |
| *UBE2E3* | | 3.3845597603465e-97 | 0.583423618503557 | 7.17560514791061e-93 | IPF |
| *CD248* | | 4.81127471920432e-85 | 0.580769297379605 | 1.02003835321851e-80 | IPF |
| *C4orf3* | | 4.82960815020158e-86 | 0.576855256427913 | 1.02392522392424e-81 | IPF |
| *THUMPD3-AS1* | | 1.97729592576331e-10 | 0.575865217022252 | 4.19206509221079e-06 | Donor |
| *BNIP3* | | 2.04565368971536e-08 | 0.575525403523773 | 0.000433699038756553 | Donor |
| *PRSS23* | | 1.29016150203125e-81 | 0.573135539154737 | 2.73527140045645e-77 | IPF |
| *FBN1* | | 1.15268114030447e-93 | 0.571309340754384 | 2.44379928555951e-89 | IPF |
| *TIMP3* | | 2.86796044063562e-53 | 0.569939827897815 | 6.08036293019158e-49 | Donor |
| *C1orf122* | | 1.74235766319344e-105 | 0.569917458379852 | 3.69397248173642e-101 | IPF |
| *ENAH* | | 1.02702125736814e-85 | 0.568287763732813 | 2.17738776774619e-81 | IPF |
| *IER3* | | 1.82296315814395e-08 | 0.566297803987041 | 0.000386486419158098 | Donor |
| *IFI27L2* | | 2.45294395275645e-91 | 0.56460763890004 | 5.20048647423894e-87 | IPF |
| *PDGFRA* | | 2.43937692360542e-10 | 0.563616011365698 | 5.17172301573585e-06 | Donor |
| *CPQ* | | 5.84327554583598e-89 | 0.562854558391809 | 1.23883284847269e-84 | IPF |
| *BST2* | | 1.35912032519216e-54 | 0.559700955559859 | 2.8814710014399e-50 | IPF |
| *FILIP1L* | | 2.74426979153725e-61 | 0.558905921352842 | 5.81812638503813e-57 | IPF |
| *SCGB1A1* | | 2.28409377098018e-41 | 0.558626598789667 | 4.84250720385507e-37 | IPF |
| *MYADM* | | 2.56223253930102e-71 | 0.557366545058721 | 5.4321892065721e-67 | IPF |
| *TNXB* | | 1.09191406777441e-56 | 0.557144222252005 | 2.31496701508852e-52 | IPF |
| *TPST1* | | 3.89712367270097e-92 | 0.556149718339973 | 8.26229189849333e-88 | IPF |
| *RAB13* | | 3.19199480161565e-64 | 0.555783274705679 | 6.76734817890534e-60 | IPF |
| *TMED3* | | 4.05591768945726e-89 | 0.554654448643413 | 8.59895109341834e-85 | IPF |
| *APCDD1* | | 3.83848144057786e-51 | 0.553714774978848 | 8.13796450216912e-47 | IPF |
| *GYPC* | | 2.37245238275377e-07 | 0.550723252642146 | 0.00502983629667627 | Donor |
| *HIF1A* | | 5.42969551883746e-62 | 0.550476950344366 | 1.15114974694873e-57 | IPF |
| *GAS6* | | 1.30930357782189e-78 | 0.550226433659755 | 2.77585451534019e-74 | IPF |
| *PPP1R15A* | | 3.39432926877125e-16 | 0.547657911201137 | 7.19631748272193e-12 | Donor |
| *NNMT* | | 4.10148276275763e-75 | 0.547109964675466 | 8.69555360532244e-71 | IPF |
| *TMEM98* | | 8.06929712040173e-91 | 0.545041271451888 | 1.71077168249637e-86 | IPF |
| *SPATS2L* | | 4.91856277257881e-78 | 0.543577017487247 | 1.04278449341443e-73 | IPF |
| *LIMCH1* | | 1.64149514931375e-05 | 0.539892930309294 | 0.348013386606008 | Donor |
| *CLMP* | | 4.45455308240688e-85 | 0.538444153105572 | 9.44409799001082e-81 | IPF |
| *GSN* | | 7.43794848434327e-19 | 0.538234596514091 | 1.57691945816562e-14 | IPF |
| *RUNX1* | | 3.41243225574066e-84 | 0.537493420076591 | 7.23469762539578e-80 | IPF |
| *SERF2* | | 1.41594834839858e-92 | 0.53744199821121 | 3.00195209343984e-88 | IPF |
| *PKM* | | 7.7710674374796e-68 | 0.535739153070403 | 1.64754400742005e-63 | IPF |
| *C9orf16* | | 5.80862344660534e-72 | 0.531029319028516 | 1.2314862569148e-67 | IPF |
| *TMSB10* | | 3.80026648894715e-116 | 0.529669998982822 | 8.05694498321685e-112 | IPF |
| *ITGBL1* | | 2.58774295111754e-33 | 0.529004327669247 | 5.4862738306643e-29 | IPF |
| *CAPZB* | | 4.42253869541487e-82 | 0.526722615567959 | 9.37622428814906e-78 | IPF |
| *VKORC1* | | 1.54450569421512e-72 | 0.526066228917789 | 3.27450652230547e-68 | IPF |
| *CDH11* | | 7.94178856865965e-63 | 0.524126595227139 | 1.68373859444153e-58 | IPF |
| *MEDAG* | | 1.44408728107556e-56 | 0.522936891655892 | 3.06160944460829e-52 | IPF |
| *FHL1* | | 1.14209584551057e-11 | 0.521974551493657 | 2.42135740206695e-07 | Donor |
| *COL4A1* | | 7.47874016038013e-57 | 0.520096850560149 | 1.58556770140219e-52 | IPF |
| *JUNB* | | 4.88567279360572e-20 | 0.520047155492897 | 1.03581148897235e-15 | Donor |
| *PAM* | | 2.71788644489755e-86 | 0.519193871728204 | 5.76219105182729e-82 | IPF |
| *ENC1* | | 7.68693362129512e-78 | 0.518882503448418 | 1.62970679705078e-73 | IPF |
| *APLP2* | | 1.33224239823223e-11 | 0.518713391536236 | 2.82448710849215e-07 | Donor |
| *SEPP1* | | 2.38223155574558e-45 | 0.518132314804101 | 5.0505691213362e-41 | Donor |
| *SRSF3* | | 2.381276436581e-16 | 0.517765445336378 | 5.04854417319537e-12 | Donor |
| *PTGFRN* | | 1.09120672683465e-94 | 0.516546455071651 | 2.31346738156214e-90 | IPF |
| *COL4A2* | | 8.00029096736194e-67 | 0.515519570605605 | 1.69614168799041e-62 | IPF |
| *PHPT1* | | 1.47600087505276e-76 | 0.514177753790763 | 3.12926945519935e-72 | IPF |
| *OSR2* | | 4.83934521651625e-55 | 0.513837076556493 | 1.02598957935361e-50 | IPF |
| *RPS2* | | 9.17482352244621e-103 | 0.511582616464258 | 1.94515433499382e-98 | Donor |
| *TPT1* | | 4.98461865318207e-96 | 0.510415578852129 | 1.05678900066113e-91 | IPF |
| *PHLDA3* | | 8.42142752129405e-73 | 0.50939485273211 | 1.78542684878955e-68 | IPF |
| *RPL35* | | 1.02421629967023e-92 | 0.50899780588006 | 2.17144097693086e-88 | Donor |
| *RHOC* | | 1.95843907260614e-73 | 0.506389712825617 | 4.15208667783229e-69 | IPF |
| *BLVRB* | | 6.11892269617677e-05 | 0.50442443676485 | 1 | Donor |
| *THBS3* | | 1.05125084888958e-91 | 0.503995536142152 | 2.2287569247308e-87 | IPF |
| *TCF21* | | 1.58589951206335e-12 | 0.498790070447093 | 3.36226555552551e-08 | Donor |
| *OAF* | | 2.99313381789267e-68 | 0.498381776186142 | 6.34574300731424e-64 | IPF |
| *GPX8* | | 1.58966320190097e-75 | 0.49835663750779 | 3.37024495435025e-71 | IPF |
| *CES1* | | 3.84741204066516e-05 | 0.496670282012089 | 0.815689826741421 | Donor |
| *TPM4* | | 8.32080932028377e-63 | 0.494944051996183 | 1.76409478399336e-58 | IPF |
| *PNRC1* | | 7.88695637077922e-15 | 0.494552831374461 | 1.6721136201689e-10 | Donor |
| *MT1X* | | 4.52023851421097e-10 | 0.493822577923021 | 9.58335767397867e-06 | IPF |
| *C4orf48* | | 9.62599001848373e-73 | 0.491575839561626 | 2.04080614381874e-68 | IPF |
| *ISG15* | | 1.15203668889343e-47 | 0.490942081212955 | 2.44243298412295e-43 | IPF |
| *SNHG8* | | 2.05970610740444e-06 | 0.489707723793722 | 0.0436678291830816 | Donor |
| *SPTSSA* | | 4.02895968118888e-53 | 0.488394767913934 | 8.54179742008855e-49 | IPF |
| *LGALS3* | | 9.3517502862842e-74 | 0.487379849319571 | 1.98266457819511e-69 | IPF |
| *CALCOCO2* | | 6.46065658560343e-06 | 0.487266876794315 | 0.136972380271378 | Donor |
| *ABCA6* | | 4.00842638013129e-11 | 0.484004235500276 | 8.49826476851634e-07 | Donor |
| *SH3PXD2A* | | 2.71701548063176e-62 | 0.483567825981138 | 5.76034452048739e-58 | IPF |
| *MMP23B* | | 4.75033095159578e-64 | 0.482819211823484 | 1.00711766504782e-59 | IPF |
| *MORF4L2* | | 1.25536651243927e-11 | 0.480905154364207 | 2.6615025430225e-07 | Donor |
| *CTSZ* | | 6.77113761444574e-66 | 0.478148908667149 | 1.43554888563864e-61 | IPF |
| *C1orf54* | | 1.37260083834203e-77 | 0.477058547074183 | 2.91005103736894e-73 | IPF |
| *RPLP0* | | 1.42098349298055e-53 | 0.47508239454376 | 3.01262710346806e-49 | Donor |
| *WBP5* | | 6.3627365699134e-63 | 0.474811936344814 | 1.34896378018734e-58 | IPF |
| *FBLN1* | | 1.01210027317569e-32 | 0.474076666054152 | 2.14575378915978e-28 | IPF |
| *RSL24D1* | | 8.53697640642904e-06 | 0.473023314277115 | 0.180992436792702 | Donor |
| *TPM2* | | 1.83992004668036e-45 | 0.470200948858237 | 3.90081449096704e-41 | IPF |
| *TAGLN2* | | 5.42723308359886e-62 | 0.469721930689733 | 1.15062768605379e-57 | IPF |
| *P4HA2* | | 2.55262685516e-67 | 0.469672940532333 | 5.41182419562472e-63 | IPF |
| *H3F3A* | | 1.27913032142634e-85 | 0.468437908896791 | 2.71188419445597e-81 | IPF |
| *PCF11* | | 3.90666024955085e-05 | 0.463500045968697 | 0.828251039507276 | Donor |
| *KDELR3* | | 3.37137443358285e-64 | 0.460825023246143 | 7.147650936639e-60 | IPF |
| *AKR7A2* | | 4.50324652304387e-76 | 0.460403095971613 | 9.5473329535053e-72 | IPF |
| *MYL9* | | 4.92016688963316e-42 | 0.460099490706302 | 1.04312458227113e-37 | IPF |
| *JUN* | | 5.25759472568629e-08 | 0.459400812424863 | 0.00111466265779275 | Donor |
| *IL1R1* | | 9.44295694005562e-69 | 0.459185835531335 | 2.00200130086119e-64 | IPF |
| *MT1E* | | 7.00964608140066e-41 | 0.458383457316094 | 1.48611506571775e-36 | IPF |
| *ECM2* | | 2.33037154907165e-67 | 0.458039682435854 | 4.94062072118681e-63 | IPF |
| *MYL6B* | | 7.97852360675762e-79 | 0.457571776418865 | 1.69152678986868e-74 | IPF |
| *TCEAL7* | | 5.14397068684454e-67 | 0.454993675795717 | 1.09057322531791e-62 | IPF |
| *PGM1* | | 3.31368898849105e-78 | 0.452146191234151 | 7.02535202449987e-74 | IPF |
| *HSP90AB1* | | 3.75900068621481e-28 | 0.450639109085922 | 7.96945735484402e-24 | Donor |
| *RPS21* | | 1.27405316035539e-27 | 0.450254078645769 | 2.70112010526947e-23 | Donor |
| *SBDS* | | 9.9892813306937e-07 | 0.449772922937681 | 0.0211782753492037 | Donor |
| *TSPAN13* | | 3.23179837900984e-67 | 0.448167933106057 | 6.85173574333875e-63 | IPF |
| *PRDX6* | | 8.01292761859012e-17 | 0.446978564224267 | 1.69882078441729e-12 | Donor |
| *MTRNR2L12* | | 2.57094408997348e-63 | 0.446577736028836 | 5.45065856515277e-59 | IPF |
| *BASP1* | | 4.79600413293735e-64 | 0.445877580643545 | 1.01680083622405e-59 | IPF |
| *UBB* | | 2.6953454147148e-76 | 0.444800039215322 | 5.71440181373685e-72 | IPF |
| *KLF4* | | 4.24594751861213e-31 | 0.444547392693368 | 9.00183333420959e-27 | IPF |
| *TMEM176B* | | 9.33145409860083e-46 | 0.440899065881583 | 1.97836158344436e-41 | IPF |
| *RBP1* | | 3.41003370278218e-69 | 0.439275091094919 | 7.2296124532685e-65 | IPF |
| *SCPEP1* | | 6.11755053461928e-64 | 0.438692315995944 | 1.29698188884463e-59 | IPF |
| *SRSF5* | | 1.61384569961192e-08 | 0.436475648621847 | 0.000342151426774724 | Donor |
| *CALM1* | | 5.64769565058013e-57 | 0.436071942919253 | 1.19736795487949e-52 | IPF |
| *HCFC1R1* | | 2.50751268662389e-59 | 0.434670432050389 | 5.3161776469113e-55 | IPF |
| *RPL18A* | | 1.37465018251689e-81 | 0.433925281554022 | 2.91439585195406e-77 | Donor |
| *CASC4* | | 2.10132373906355e-72 | 0.433899759505872 | 4.45501645918864e-68 | IPF |
| *MARCKSL1* | | 7.99594532596736e-74 | 0.433694165180369 | 1.69522036855834e-69 | IPF |
| *CYBA* | | 1.57804856332076e-82 | 0.433634054265143 | 3.34562075909635e-78 | IPF |
| *GUCY1A3* | | 1.21714113623284e-66 | 0.431658896877468 | 2.58046092292725e-62 | IPF |
| *RPL36* | | 1.15890250557284e-57 | 0.430946551377091 | 2.45698920206498e-53 | Donor |
| *FKBP10* | | 5.45028094126642e-70 | 0.430405278649115 | 1.15551406235789e-65 | IPF |
| *PRAF2* | | 8.1561390285624e-76 | 0.429287198777511 | 1.72918303544551e-71 | IPF |
| *PRRX1* | | 2.3873624705598e-72 | 0.428792364762929 | 5.06144717383383e-68 | IPF |
| *LTBP3* | | 1.70260093621619e-69 | 0.428581070784036 | 3.60968424487195e-65 | IPF |
| *MAP4K4* | | 1.93077447893727e-67 | 0.428202944926215 | 4.09343497279491e-63 | IPF |
| *B2M* | | 1.55703572482274e-108 | 0.426704766691638 | 3.30107144019669e-104 | IPF |
| *CCNL1* | | 1.11277154092533e-05 | 0.426518266834237 | 0.23591869439158 | Donor |
| *ANGPTL2* | | 2.25225373698695e-61 | 0.42611611075567 | 4.77500314778603e-57 | IPF |
| *SH3BGRL* | | 6.40077093486596e-63 | 0.425698689410779 | 1.35702744590093e-58 | IPF |
| *TSPAN4* | | 8.60703382073765e-05 | 0.425278714040549 | 1 | Donor |
| *COPZ2* | | 6.79128160707524e-67 | 0.425169927994157 | 1.43981961351602e-62 | IPF |
| *NDUFS5* | | 2.71358343803332e-56 | 0.42357230951892 | 5.75306824697444e-52 | IPF |
| *C1QTNF2* | | 2.79420223819983e-68 | 0.423547846251932 | 5.92398816520746e-64 | IPF |
| *CBX3* | | 9.34365453598652e-60 | 0.423050964248502 | 1.9809481981745e-55 | IPF |
| *EMP3* | | 7.42885806091313e-62 | 0.421540296888287 | 1.57499219749419e-57 | IPF |
| *RPL21* | | 2.21985520721011e-90 | 0.418559807778556 | 4.70631502480616e-86 | Donor |
| *VMP1* | | 3.9817465714912e-51 | 0.417840175978478 | 8.4417009062185e-47 | IPF |
| *LTBP4* | | 2.01305523171121e-07 | 0.416764871672838 | 0.00426787839675094 | Donor |
| *PRDX4* | | 3.50162884731894e-68 | 0.416492490371614 | 7.42380331920088e-64 | IPF |
| *SOX4* | | 4.58455699866179e-59 | 0.415108421993903 | 9.71971929286285e-55 | IPF |
| *JUND* | | 3.26637405745465e-40 | 0.414905407401652 | 6.9250396392096e-36 | IPF |
| *NEU1* | | 1.12835453464186e-55 | 0.414351327353756 | 2.39222444889422e-51 | IPF |
| *MXRA8* | | 3.40365930138065e-54 | 0.41379026131806 | 7.21609808485712e-50 | IPF |
| *RPS18* | | 1.61621326566848e-89 | 0.410176359749046 | 3.42653374454374e-85 | Donor |
| *CHID1* | | 2.37076416858971e-61 | 0.409947187027182 | 5.02625711382705e-57 | IPF |
| *GNG11* | | 1.77368887545981e-62 | 0.407172026388575 | 3.76039778486233e-58 | IPF |
| *RPS5* | | 2.30724563270992e-58 | 0.406526874134465 | 4.89159146590831e-54 | Donor |
| *S100A13* | | 5.69490460149522e-53 | 0.405267411672777 | 1.207376724563e-48 | IPF |
| *TP53I3* | | 1.52549762702335e-67 | 0.405205580087369 | 3.23420751905221e-63 | IPF |
| *HMCN1* | | 3.67060496646909e-73 | 0.404137697179667 | 7.78204958941111e-69 | IPF |
| *CETN2* | | 2.50357908496138e-58 | 0.403006140166091 | 5.30783801802662e-54 | IPF |
| *CHPF* | | 3.24443206461334e-55 | 0.401728173542186 | 6.87852042018674e-51 | IPF |
| *KLF6* | | 2.45816448631876e-22 | 0.400764499967119 | 5.21155452744441e-18 | IPF |
| *P3H3* | | 2.55295613772389e-79 | 0.400420748051753 | 5.41252230758842e-75 | IPF |
| *BICC1* | | 7.50686260937103e-53 | 0.398881522452364 | 1.59152994181275e-48 | IPF |
| *CYB5R3* | | 1.73879997974386e-59 | 0.398130376966296 | 3.68642983705496e-55 | IPF |
| *PFN2* | | 7.32286291325987e-65 | 0.397686947252443 | 1.55252016624023e-60 | IPF |
| *ARPC2* | | 1.1099936583994e-56 | 0.39662341698799 | 2.35329755517258e-52 | IPF |
| *PRKCDBP* | | 1.58831009218337e-56 | 0.396169797731258 | 3.36737622643797e-52 | IPF |
| *GLIPR1* | | 9.46423192353511e-61 | 0.395295773200178 | 2.00651181010868e-56 | IPF |
| *PIGT* | | 2.36250616567513e-62 | 0.394877350904226 | 5.00874932184784e-58 | IPF |
| *PRDX2* | | 2.00475795305526e-58 | 0.392342035211268 | 4.25028733627245e-54 | IPF |
| *TXNIP* | | 4.55074989414281e-10 | 0.391997220162144 | 9.64804485057217e-06 | Donor |
| *RPS14* | | 2.55655091066772e-73 | 0.390985259378019 | 5.42014358570664e-69 | Donor |
| *CMTM3* | | 2.86907778009927e-60 | 0.389930454419194 | 6.08273180158846e-56 | IPF |
| *CTSO* | | 1.46035436833636e-60 | 0.389769084902658 | 3.09609729630992e-56 | IPF |
| *ZBTB16* | | 4.23110750516736e-08 | 0.389586376573543 | 0.000897037102170533 | Donor |
| *PTEN* | | 2.37768501678145e-59 | 0.387995094384249 | 5.04093000407836e-55 | IPF |
| *DUSP1* | | 5.11119048448549e-17 | 0.38754643683945 | 1.08362349461577e-12 | IPF |
| *MAGED1* | | 7.30953072288522e-72 | 0.38743588116577 | 1.54969360855889e-67 | IPF |
| *C16orf89* | | 4.26172635969399e-43 | 0.383231095615676 | 9.03528605518723e-39 | IPF |
| *ZNF428* | | 4.47837238027291e-68 | 0.381927594206127 | 9.49459728341659e-64 | IPF |
| *SELM* | | 5.06750704013556e-48 | 0.381663252448735 | 1.07436216757914e-43 | IPF |
| *ACTB* | | 5.64222221151145e-33 | 0.381596767260649 | 1.19620753106254e-28 | Donor |
| *CALU* | | 7.13866668335212e-61 | 0.377698400487517 | 1.51346872353748e-56 | IPF |
| *GABARAPL2* | | 3.78311504706039e-48 | 0.37610767026518 | 8.02058221127273e-44 | IPF |
| *FNIP1* | | 8.30173283249775e-57 | 0.374873240668106 | 1.76005037781785e-52 | IPF |
| *ST3GAL4* | | 1.69730205554976e-53 | 0.37481008522728 | 3.59845008797106e-49 | IPF |
| *WLS* | | 9.9053672159459e-63 | 0.374504431474705 | 2.10003690345269e-58 | IPF |
| *TOMM7* | | 1.36323273029531e-24 | 0.37385165397278 | 2.89018971149908e-20 | Donor |
| *GTF2I* | | 7.62188760433884e-60 | 0.373570720168669 | 1.61591639099588e-55 | IPF |
| *TUSC3* | | 8.25603066581697e-72 | 0.371925441397406 | 1.75036106145986e-67 | IPF |
| *ZFAS1* | | 5.25460456477932e-06 | 0.370862514221493 | 0.111402871377886 | Donor |
| *UBE2D3* | | 5.75087399987593e-07 | 0.369897040479921 | 0.012192427967137 | Donor |
| *TSC22D1* | | 1.41360112723949e-20 | 0.369738804378001 | 2.99697574986044e-16 | IPF |
| *NUPR1* | | 2.64676208150626e-12 | 0.368650187409543 | 5.61140028900143e-08 | Donor |
| *QPRT* | | 2.85924163436512e-54 | 0.366592150866407 | 6.06187818901749e-50 | IPF |
| *COX6C* | | 1.06471297875702e-41 | 0.366340091974527 | 2.25729798626275e-37 | IPF |
| *CHD3* | | 7.12252306741084e-43 | 0.365847572110196 | 1.51004611552177e-38 | IPF |
| *RPS28* | | 1.59632131801449e-55 | 0.364056120823401 | 3.38436082632251e-51 | Donor |
| *EMILIN1* | | 2.60232436844108e-35 | 0.363900545059274 | 5.51718789353193e-31 | IPF |
| *UAP1* | | 1.28532366103062e-07 | 0.362716954559035 | 0.00272501469375103 | Donor |
| *BAX* | | 8.0947971984736e-61 | 0.362496729912191 | 1.71617795404839e-56 | IPF |
| *ATOX1* | | 1.25586423139346e-55 | 0.362291835661215 | 2.66255775697728e-51 | IPF |
| *RCN1* | | 3.56907604154688e-47 | 0.36208042965232 | 7.56679811568353e-43 | IPF |
| *RPS25* | | 1.00878015309441e-55 | 0.360403024163465 | 2.13871480257545e-51 | Donor |
| *ACTG1* | | 1.51118416274922e-15 | 0.36024562939699 | 3.20386154344461e-11 | Donor |
| *RPL10* | | 1.28127021164698e-72 | 0.360183082777173 | 2.71642097571277e-68 | Donor |
| *AEBP1* | | 2.18523846914953e-37 | 0.360105577661112 | 4.63292407844393e-33 | IPF |
| *MINOS1* | | 2.19322834281525e-49 | 0.359783702265118 | 4.64986340960261e-45 | IPF |
| *FAM127A* | | 1.0354525370871e-63 | 0.359632786579199 | 2.19526292387837e-59 | IPF |
| *SSR4* | | 3.31273102542696e-44 | 0.357705006778011 | 7.02332104700769e-40 | IPF |
| *TCF4* | | 9.01849053372492e-43 | 0.357571482191569 | 1.91201017805502e-38 | IPF |
| *MTRNR2L8* | | 7.42377347335565e-48 | 0.356475934684047 | 1.57391421408613e-43 | IPF |
| *SH3PXD2B* | | 5.7543660479126e-56 | 0.35562993060546 | 1.21998314581795e-51 | IPF |
| *HSBP1* | | 3.36530252380037e-53 | 0.354871795616777 | 7.13477788070917e-49 | IPF |
| *EIF3E* | | 5.53109956116559e-12 | 0.353221387749907 | 1.17264841796272e-07 | Donor |
| *FAM114A1* | | 1.35872929854287e-55 | 0.3528109077774 | 2.88064198584074e-51 | IPF |
| *RPL11* | | 5.68379984113132e-55 | 0.352369178539036 | 1.20502240431825e-50 | Donor |
| *TCF12* | | 3.46416018158249e-59 | 0.351978288945351 | 7.34436600097304e-55 | IPF |
| *KCTD12* | | 8.90435223499162e-52 | 0.35159880337632 | 1.88781171734057e-47 | IPF |
| *LINC01420* | | 3.55708755857557e-58 | 0.349277778504541 | 7.54138133293607e-54 | IPF |
| *TUBB2A* | | 1.26122024067149e-34 | 0.349156275701253 | 2.67391303224762e-30 | IPF |
| *S100A11* | | 3.61042074076969e-57 | 0.348678941606524 | 7.65445301250582e-53 | IPF |
| *PTOV1* | | 3.8975911788221e-63 | 0.348291779999423 | 8.26328305822073e-59 | IPF |
| *RPSA* | | 6.43629797112119e-25 | 0.34744345081918 | 1.3645595328574e-20 | Donor |
| *LGALS1* | | 1.06218694867853e-21 | 0.347344691535795 | 2.25194254989335e-17 | IPF |
| *PIEZO2* | | 2.10225454774003e-47 | 0.346867186971635 | 4.45698986666364e-43 | IPF |
| *SH3BGRL3* | | 6.13684903393722e-42 | 0.346705075982011 | 1.30107336368503e-37 | IPF |
| *RORA* | | 8.63190602110942e-42 | 0.345587508396705 | 1.83005039553541e-37 | IPF |
| *CCS* | | 4.16364235138058e-58 | 0.345572555959446 | 8.82733814916196e-54 | IPF |
| *RPS7* | | 6.57732422749315e-38 | 0.344840702854789 | 1.39445850947082e-33 | Donor |
| *RPS8* | | 2.82446317816512e-56 | 0.344232025867177 | 5.98814438402788e-52 | Donor |
| *FGF7* | | 7.45361646515027e-29 | 0.344081917885555 | 1.58024122677651e-24 | IPF |
| *MAGED2* | | 1.32267585531758e-51 | 0.342759594075398 | 2.80420508085881e-47 | IPF |
| *RTN4* | | 1.6598991736093e-08 | 0.342274483011161 | 0.000351915223796907 | Donor |
| *NDN* | | 4.01189012978861e-59 | 0.341273653090207 | 8.50560826416483e-55 | IPF |
| *SPSB1* | | 1.52217818199444e-47 | 0.340298014542341 | 3.22716996364642e-43 | IPF |
| *CTD-3252C9.4* | | 2.07595800102201e-39 | 0.340211384876145 | 4.40123855796676e-35 | IPF |
| *COL16A1* | | 1.35105322597674e-47 | 0.33876233129555 | 2.86436794439328e-43 | IPF |
| *DYNLL1* | | 6.84180454678196e-37 | 0.337390289002984 | 1.45053098196324e-32 | IPF |
| *RPL31* | | 3.93003604430305e-37 | 0.336733343510986 | 8.33206941752689e-33 | Donor |
| *ITGAV* | | 2.49631242618023e-62 | 0.336146573542675 | 5.2924319747447e-58 | IPF |
| *STEAP2* | | 2.03237899826997e-46 | 0.336056054752238 | 4.30884671423216e-42 | IPF |
| *FKBP5* | | 1.23329089640741e-22 | 0.335720179839024 | 2.61470002947334e-18 | IPF |
| *FKBP7* | | 3.5584288558165e-55 | 0.334974988994421 | 7.54422501721657e-51 | IPF |
| *STMN3* | | 5.82211111203187e-64 | 0.334781427890455 | 1.23434577686188e-59 | IPF |
| *RPLP2* | | 2.24376233378351e-57 | 0.334735425705105 | 4.75700052385442e-53 | Donor |
| *OSTC* | | 1.05371354636752e-47 | 0.333350689508831 | 2.23397808965378e-43 | IPF |
| *OLFML3* | | 3.17723066625338e-35 | 0.331757976437089 | 6.73604673552378e-31 | IPF |
| *SPATS2* | | 5.38786650914807e-57 | 0.331698520018972 | 1.14228157860448e-52 | IPF |
| *SNX9* | | 1.82706471100812e-47 | 0.330789537502891 | 3.87355989380831e-43 | IPF |
| *RBMS1* | | 1.81975177130587e-45 | 0.330764787443387 | 3.85805573034558e-41 | IPF |
| *EVA1B* | | 1.83974619140986e-60 | 0.330557301846841 | 3.90044590040804e-56 | IPF |
| *TPBG* | | 2.1343734289081e-52 | 0.330284763819943 | 4.52508510662807e-48 | IPF |
| *TWSG1* | | 6.82775409387495e-57 | 0.330211542523216 | 1.44755214544243e-52 | IPF |
| *NFIX* | | 5.67597217380056e-49 | 0.329650569208488 | 1.20336286056746e-44 | IPF |
| *FCGRT* | | 2.59925145670692e-47 | 0.329544472103283 | 5.51067301336435e-43 | IPF |
| *ANXA5* | | 1.14169196549643e-35 | 0.328894324978949 | 2.42050113604899e-31 | IPF |
| *ADD3* | | 7.01188633182362e-37 | 0.328598152246052 | 1.48659002120992e-32 | IPF |
| *FSTL1* | | 6.77318521564964e-39 | 0.327986646311742 | 1.43598299756988e-34 | IPF |
| *C11orf31* | | 3.82697764755234e-49 | 0.326237632949119 | 8.11357531057572e-45 | IPF |
| *RPS27* | | 3.49601734613001e-55 | 0.325508853758156 | 7.41190637553024e-51 | Donor |
| *RHOBTB3* | | 8.30598508299499e-42 | 0.325279601915358 | 1.76095189744577e-37 | IPF |
| *ARF4* | | 1.09602267016018e-43 | 0.325118903582867 | 2.3236776630066e-39 | IPF |
| *HLA-B* | | 1.38188809296509e-46 | 0.324957865958794 | 2.92974094589528e-42 | IPF |
| *HES4* | | 2.35689861099993e-48 | 0.324260140372907 | 4.99686074518096e-44 | IPF |
| *TMOD3* | | 2.35283575995229e-53 | 0.324119978799568 | 4.98824709467485e-49 | IPF |
| *C10orf10* | | 2.90478902789512e-08 | 0.32369705250231 | 0.000615844321804045 | Donor |
| *BNC2* | | 1.73033855870367e-51 | 0.323556962916952 | 3.66849077830765e-47 | IPF |
| *CHMP4B* | | 2.72748561735252e-55 | 0.323486963959222 | 5.78254225734908e-51 | IPF |
| *BSG* | | 2.71863715419364e-44 | 0.322794399443696 | 5.76378263060594e-40 | IPF |
| *SEPT11* | | 2.96360101706471e-41 | 0.322781643283056 | 6.28313051627889e-37 | IPF |
| *BHLHE40* | | 5.32096543175129e-41 | 0.321796428202911 | 1.12809788118559e-36 | IPF |
| *KDELR2* | | 1.05166838931346e-40 | 0.321254870622925 | 2.22964215218346e-36 | IPF |
| *LUZP1* | | 1.64557142010802e-55 | 0.319799276768444 | 3.48877596777101e-51 | IPF |
| *COLEC12* | | 1.29945733389392e-35 | 0.319602262301246 | 2.7549794935885e-31 | IPF |
| *NDUFA13* | | 2.86075145848642e-35 | 0.319007381159148 | 6.06507916713705e-31 | IPF |
| *RPL34* | | 1.07419509240595e-44 | 0.318814243216574 | 2.27740101540985e-40 | Donor |
| *LGALS3BP* | | 7.62083641788566e-40 | 0.318626051259577 | 1.61569352895594e-35 | IPF |
| *PLAT* | | 4.86117372916453e-36 | 0.31854138649773 | 1.03061744232017e-31 | IPF |
| *ITGB5* | | 8.06995042572234e-51 | 0.318088799992435 | 1.71091018975739e-46 | IPF |
| *RBFOX2* | | 8.17174253120588e-57 | 0.317725165462119 | 1.73249113404096e-52 | IPF |
| *SNRPN* | | 6.41767985481566e-47 | 0.314748095154593 | 1.36061230601947e-42 | IPF |
| *OST4* | | 4.27882565166553e-40 | 0.314264014513189 | 9.07153826409609e-36 | IPF |
| *EIF4G2* | | 1.66049851269219e-46 | 0.314022046174101 | 3.52042289675871e-42 | IPF |
| *NPC2* | | 6.36701824748091e-35 | 0.313851460729702 | 1.34987153864843e-30 | IPF |
| *PRDX5* | | 1.31353844903954e-41 | 0.313797167286666 | 2.78483286580872e-37 | IPF |
| *TAX1BP3* | | 6.25742782728945e-53 | 0.313583753731427 | 1.32663727366364e-48 | IPF |
| *ATP6V0E1* | | 3.9412351573508e-36 | 0.313067682103713 | 8.35581265709944e-32 | IPF |
| *IKBIP* | | 1.29686348583167e-59 | 0.312742934211861 | 2.74948027631173e-55 | IPF |
| *NAGK* | | 2.45941274334484e-52 | 0.312215160516395 | 5.21420095716541e-48 | IPF |
| *TMEM230* | | 5.56773894586135e-36 | 0.312149578586358 | 1.18041633391206e-31 | IPF |
| *RPL32* | | 1.25662023349627e-46 | 0.311321012231699 | 2.66416055703544e-42 | Donor |
| *AKAP13* | | 1.85780891804055e-39 | 0.311154816862586 | 3.93874068713777e-35 | IPF |
| *GEM* | | 2.0665604655257e-64 | 0.310783947674975 | 4.38131484296103e-60 | IPF |
| *FGFR1* | | 1.06714556689464e-38 | 0.310508070458055 | 2.26245531637332e-34 | IPF |
| *S100A16* | | 4.69351421238572e-44 | 0.310464917736324 | 9.95071948167896e-40 | IPF |
| *SPRY1* | | 7.39999628663831e-21 | 0.308139381167023 | 1.56887321273019e-16 | IPF |
| *PROS1* | | 1.51121368337481e-43 | 0.30551959501973 | 3.20392413012292e-39 | IPF |
| *NUCKS1* | | 2.44245324629267e-40 | 0.304224929795028 | 5.17824512746509e-36 | IPF |
| *TMEM45A* | | 1.97496240740981e-57 | 0.303616016886375 | 4.18711779994954e-53 | IPF |
| *RPL14* | | 1.23767848772136e-31 | 0.302620591241962 | 2.62400216181806e-27 | Donor |
| *CYGB* | | 4.84065304422825e-41 | 0.302190127219909 | 1.02626685190683e-36 | IPF |
| *MPZL1* | | 2.78131409639009e-55 | 0.302118890205066 | 5.89666401575663e-51 | IPF |
| *SNAI2* | | 1.39851612772889e-42 | 0.301707433543037 | 2.96499404239803e-38 | IPF |
| *AFF4* | | 1.61982398972338e-50 | 0.300524925071293 | 3.43418884061254e-46 | IPF |
| *FAM229B* | | 1.02054130908526e-45 | 0.299768702593747 | 2.16364962939166e-41 | IPF |
| *IFI6* | | 2.82563145970526e-43 | 0.298476297934505 | 5.99062125772112e-39 | IPF |
| *PPP1CC* | | 3.37381244836004e-41 | 0.298305967039198 | 7.15281977176813e-37 | IPF |
| *SCARB2* | | 7.84161276926434e-47 | 0.297979551718508 | 1.66250032321173e-42 | IPF |
| *PLAGL1* | | 8.3984496497373e-38 | 0.297654227927277 | 1.7805553102408e-33 | IPF |
| *RPL18* | | 2.56240702440573e-34 | 0.297369397010157 | 5.43255913244258e-30 | Donor |
| *METTL9* | | 1.22259052313002e-58 | 0.296193943593855 | 2.59201416808796e-54 | IPF |
| *GNAS* | | 2.20153615816826e-48 | 0.295209358677786 | 4.66747680893254e-44 | IPF |
| *CHMP3* | | 2.19064696463252e-48 | 0.293848745101838 | 4.6443906297174e-44 | IPF |
| *JTB* | | 3.66546534180904e-38 | 0.293847727962952 | 7.77115307116935e-34 | IPF |
| *TP53I13* | | 1.00604501439009e-50 | 0.293732757776509 | 2.13291603500843e-46 | IPF |
| *RUNX1T1* | | 1.18353089286568e-36 | 0.293629454459279 | 2.50920384596454e-32 | IPF |
| *SEC31A* | | 4.88246607787385e-49 | 0.291001366157939 | 1.03513163317004e-44 | IPF |
| *ANXA1* | | 1.02197025988914e-16 | 0.289673548422526 | 2.16667914799098e-12 | IPF |
| *SAT1* | | 1.56836539112117e-08 | 0.289540555382175 | 0.0003325091465716 | Donor |
| *TCEB1* | | 1.82067330874487e-05 | 0.288067041086841 | 0.386000948187001 | Donor |
| *SPON1* | | 1.28253467815023e-52 | 0.288029965718201 | 2.7191017711463e-48 | IPF |
| *RPS26* | | 2.6682396224945e-05 | 0.2875826378749 | 0.565693482365059 | Donor |
| *RPS15* | | 2.98023148525167e-41 | 0.287383065155634 | 6.31838877188207e-37 | Donor |
| *SVIL* | | 9.55541010205252e-41 | 0.287030869580333 | 2.02584249573615e-36 | IPF |
| *DNPH1* | | 6.90826652553654e-51 | 0.286099908469888 | 1.464621586079e-46 | IPF |
| *SLC25A6* | | 9.50100675866494e-12 | 0.285888618275047 | 2.01430844290455e-07 | Donor |
| *A4GALT* | | 7.70195486407897e-41 | 0.284661483192986 | 1.63289145073338e-36 | IPF |
| *TMEM176A* | | 8.59703142077845e-32 | 0.284641106585136 | 1.82265663151924e-27 | IPF |
| *SET* | | 4.70580605794682e-43 | 0.284398076409953 | 9.97677942345306e-39 | IPF |
| *FUS* | | 9.16839580780898e-33 | 0.284267214574352 | 1.94379159521358e-28 | IPF |
| *PLD3* | | 3.04623582998355e-38 | 0.283888989583462 | 6.45832458314813e-34 | IPF |
| *PLOD2* | | 1.63339428708897e-42 | 0.283705793321464 | 3.46295922805732e-38 | IPF |
| *YWHAZ* | | 1.10498769036567e-42 | 0.282384282554644 | 2.34268440234426e-38 | IPF |
| *CAV1* | | 6.37790270467052e-21 | 0.282252242620731 | 1.3521791524172e-16 | IPF |
| *PGAM1* | | 1.03042176418402e-49 | 0.281438302740409 | 2.18459718224655e-45 | IPF |
| *UBE2E2* | | 5.14698431207093e-51 | 0.2809526816848 | 1.09121214400216e-46 | IPF |
| *ZNHIT1* | | 1.74642339523284e-47 | 0.280479893221281 | 3.70259224023313e-43 | IPF |
| *UGCG* | | 1.31725512134833e-42 | 0.28047660829552 | 2.79271258277059e-38 | IPF |
| *SUMO2* | | 8.18756059442507e-38 | 0.280304324238619 | 1.73584472162406e-33 | IPF |
| *SDC2* | | 2.68802118513157e-26 | 0.280261365454973 | 5.69887371459743e-22 | IPF |
| *AKR1C1* | | 7.29312031540903e-27 | 0.279231807058769 | 1.54621443806987e-22 | IPF |
| *SLC2A3* | | 8.70649105367373e-40 | 0.279223928181574 | 1.84586316828937e-35 | IPF |
| *RHOA* | | 2.57037857561455e-34 | 0.278838084217319 | 5.44945961816042e-30 | IPF |
| *PLEKHA4* | | 1.72889753728588e-46 | 0.278542216693476 | 3.66543566879979e-42 | IPF |
| *CDK4* | | 3.15550654538298e-49 | 0.278060067331079 | 6.68998942686645e-45 | IPF |
| *SMARCA2* | | 1.18228672871265e-36 | 0.2777184959668 | 2.5065660935437e-32 | IPF |
| *RPS16* | | 5.70794841660596e-37 | 0.277631476519868 | 1.21014214380463e-32 | Donor |
| *FKBP9* | | 7.41820477500126e-47 | 0.277264834084677 | 1.57273359434802e-42 | IPF |
| *SPG21* | | 3.35285733066911e-49 | 0.277245505910125 | 7.10839282675157e-45 | IPF |
| *UQCC2* | | 9.62659760066301e-52 | 0.277108711344141 | 2.04093495731656e-47 | IPF |
| *PTMS* | | 4.08980336585007e-40 | 0.276608591342048 | 8.67079211593873e-36 | IPF |
| *TXNL4A* | | 9.02072825131779e-46 | 0.276560726649811 | 1.91248459656189e-41 | IPF |
| *HTRA3* | | 1.12869120182398e-43 | 0.276251730838574 | 2.39293821698703e-39 | IPF |
| *TGFB3* | | 5.05216398221511e-48 | 0.274948640676901 | 1.07110928586943e-43 | IPF |
| *NBEAL1* | | 4.88970782952876e-09 | 0.274197416814422 | 0.000103666695693839 | Donor |
| *GUK1* | | 2.16381234816671e-38 | 0.273796032992596 | 4.58749855934825e-34 | IPF |
| *MMP14* | | 4.15461773094917e-30 | 0.273584898022909 | 8.80820505138534e-26 | IPF |
| *SEC13* | | 2.5106686350095e-43 | 0.273478954918438 | 5.32286857308365e-39 | IPF |
| *UQCRB* | | 4.73812513429814e-28 | 0.272891790751911 | 1.00452990972255e-23 | IPF |
| *GOLPH3* | | 1.70909091036026e-44 | 0.272401909885528 | 3.62344363905479e-40 | IPF |
| *MT2A* | | 1.78289355431839e-18 | 0.272391194113045 | 3.77991262451042e-14 | IPF |
| *RPL7* | | 6.91274545540355e-34 | 0.272331993917987 | 1.46557116400011e-29 | Donor |
| *DDIT4* | | 2.06691632595224e-12 | 0.271356000920304 | 4.38206930265135e-08 | IPF |
| *SGK1* | | 3.39807860371608e-17 | 0.270872632016065 | 7.20426644773845e-13 | IPF |
| *CAPNS1* | | 3.0764266029421e-43 | 0.27041396711404 | 6.52233204089755e-39 | IPF |
| *GPRC5A* | | 2.02498038047278e-28 | 0.270373992135244 | 4.29316090464034e-24 | IPF |
| *HSPH1* | | 2.34512699364453e-38 | 0.269601037198307 | 4.97190373922577e-34 | IPF |
| *TYMP* | | 2.49907676470757e-37 | 0.269352735343842 | 5.29829264885653e-33 | IPF |
| *SCP2* | | 2.4373785277752e-39 | 0.269220969531975 | 5.16748621673619e-35 | IPF |
| *RPL35A* | | 1.83140906547651e-30 | 0.268487132335827 | 3.88277035971676e-26 | Donor |
| *MCTS1* | | 2.28250952175753e-50 | 0.26741988765385 | 4.83914843707813e-46 | IPF |
| *PPP1R2* | | 2.48300133869047e-34 | 0.266989116377944 | 5.26421113815766e-30 | IPF |
| *ETHE1* | | 7.43398005636536e-50 | 0.266567293362724 | 1.57607811175002e-45 | IPF |
| *ZCRB1* | | 1.00121990463325e-52 | 0.26623238842445 | 2.12268631981295e-48 | IPF |
| *EIF3J* | | 5.85766017870544e-28 | 0.265435289010497 | 1.24188253448734e-23 | IPF |
| *DYNLRB1* | | 1.65386691734696e-41 | 0.264674744418384 | 3.50636325146729e-37 | IPF |
| *CDC42* | | 1.62955935887562e-38 | 0.26433927968388 | 3.45482879675221e-34 | IPF |
| *NUCB2* | | 1.55214282391612e-56 | 0.264264891925857 | 3.29069800098457e-52 | IPF |
| *PFN1* | | 3.85323471409533e-32 | 0.263956575079546 | 8.16924291735351e-28 | IPF |
| *WWTR1* | | 3.78540434684467e-41 | 0.263789823613614 | 8.02543575574539e-37 | IPF |
| *RPS12* | | 1.77816783658411e-24 | 0.263719524844554 | 3.76989363034198e-20 | Donor |
| *BAD* | | 1.67244733976261e-51 | 0.263699771333494 | 3.54575560503072e-47 | IPF |
| *EMC10* | | 1.59240346284317e-43 | 0.26302508928762 | 3.3760545815738e-39 | IPF |
| *SDF4* | | 1.01261570010814e-45 | 0.26270268908473 | 2.14684654579928e-41 | IPF |
| *GGCT* | | 1.76487799184346e-51 | 0.262462227325246 | 3.74171783050732e-47 | IPF |
| *LMO4* | | 1.30143490208375e-29 | 0.261880126337489 | 2.75917213590776e-25 | IPF |
| *CKAP4* | | 4.51808101975244e-49 | 0.261630498724962 | 9.57878356997715e-45 | IPF |
| *HDLBP* | | 3.34732631739258e-47 | 0.260709144577499 | 7.096666525504e-43 | IPF |
| *PLOD1* | | 8.40706276059079e-43 | 0.26027932657423 | 1.78238137587285e-38 | IPF |
| *AHR* | | 1.34527474514514e-38 | 0.259852497719027 | 2.85211698718221e-34 | IPF |
| *FKBP11* | | 1.95980578093449e-48 | 0.259825003465903 | 4.15498423615921e-44 | IPF |
| *GNAI2* | | 3.72566415829497e-41 | 0.259122261191498 | 7.89878058200117e-37 | IPF |
| *SEPT2* | | 7.04209843132436e-41 | 0.25821935449927 | 1.49299528842508e-36 | IPF |
| *FXYD5* | | 1.53001547285225e-37 | 0.257540840184296 | 3.24378580399405e-33 | IPF |
| *RPL37A* | | 4.49945442345813e-30 | 0.257430423761024 | 9.53929332317359e-26 | Donor |
| *TCEAL8* | | 1.63710794809364e-42 | 0.257330907785297 | 3.47083256075332e-38 | IPF |
| *ZBTB20* | | 1.02034884440223e-31 | 0.257097772681429 | 2.16324158501716e-27 | IPF |
| *VAT1* | | 3.79954093602738e-42 | 0.256534482808185 | 8.05540673847165e-38 | IPF |
| *DYNLT1* | | 5.10371939872074e-49 | 0.256204690934862 | 1.08203954972278e-44 | IPF |
| *UBE2L6* | | 8.9110030622129e-42 | 0.255281316784485 | 1.88922175921976e-37 | IPF |
| *NENF* | | 1.1325244012229e-38 | 0.25510361034426 | 2.40106498303268e-34 | IPF |
| *TPI1* | | 1.45081847839257e-30 | 0.255038837919582 | 3.07588025604009e-26 | IPF |
| *TUBB* | | 1.5272105668953e-35 | 0.255019104170115 | 3.23783912287473e-31 | IPF |
| *LAMB2* | | 1.53758559359294e-37 | 0.254868295730131 | 3.25983521697639e-33 | IPF |
| *AC090498.1* | | 7.01174699763868e-05 | 0.254507469775294 | 1 | Donor |
| *ARF3* | | 2.2637416983071e-39 | 0.254247063677916 | 4.79935877458088e-35 | IPF |
| *RPS27L* | | 3.8305070793398e-28 | 0.254160890885466 | 8.12105805890831e-24 | IPF |
| *RAB34* | | 3.41353083599301e-42 | 0.253740295044704 | 7.23702672538879e-38 | IPF |
| *PLSCR4* | | 7.64221461547649e-35 | 0.253586449353211 | 1.62022592062717e-30 | IPF |
| *RNF24* | | 5.19180329592033e-51 | 0.252631423222841 | 1.10071421676807e-46 | IPF |
| *RPL10A* | 3.66469624124739e-27 | | 0.252240602889833 | 7.76952250106858e-23 | Donor |
| *DECR1* | 1.71353857624154e-44 | | 0.252093941199695 | 3.63287313548968e-40 | IPF |
| *ATPIF1* | 2.60523779843861e-39 | | 0.251956000492948 | 5.52336465646969e-35 | IPF |
| *EEF1A1* | 3.45033577955907e-37 | | 0.251667210380628 | 7.31505688624319e-33 | IPF |
| *PPIB* | 2.09960407122388e-26 | | 0.250569229235522 | 4.45137059140175e-22 | IPF |
| *SGCB* | 1.35597853369352e-44 | | 0.250498308091435 | 2.87481008928363e-40 | IPF |
| *PET100* | 1.94530668359923e-28 | | 0.25022184118329 | 4.12424469989873e-24 | IPF |
| *INMT* | 8.5884467951271e-98 | | 1.9719891 | 1.8208366050349e-93 | Donor |
| *SOD2* | 2.26253543042241e-14 | | 1.0528532 | 4.79680136603854e-10 | Donor |


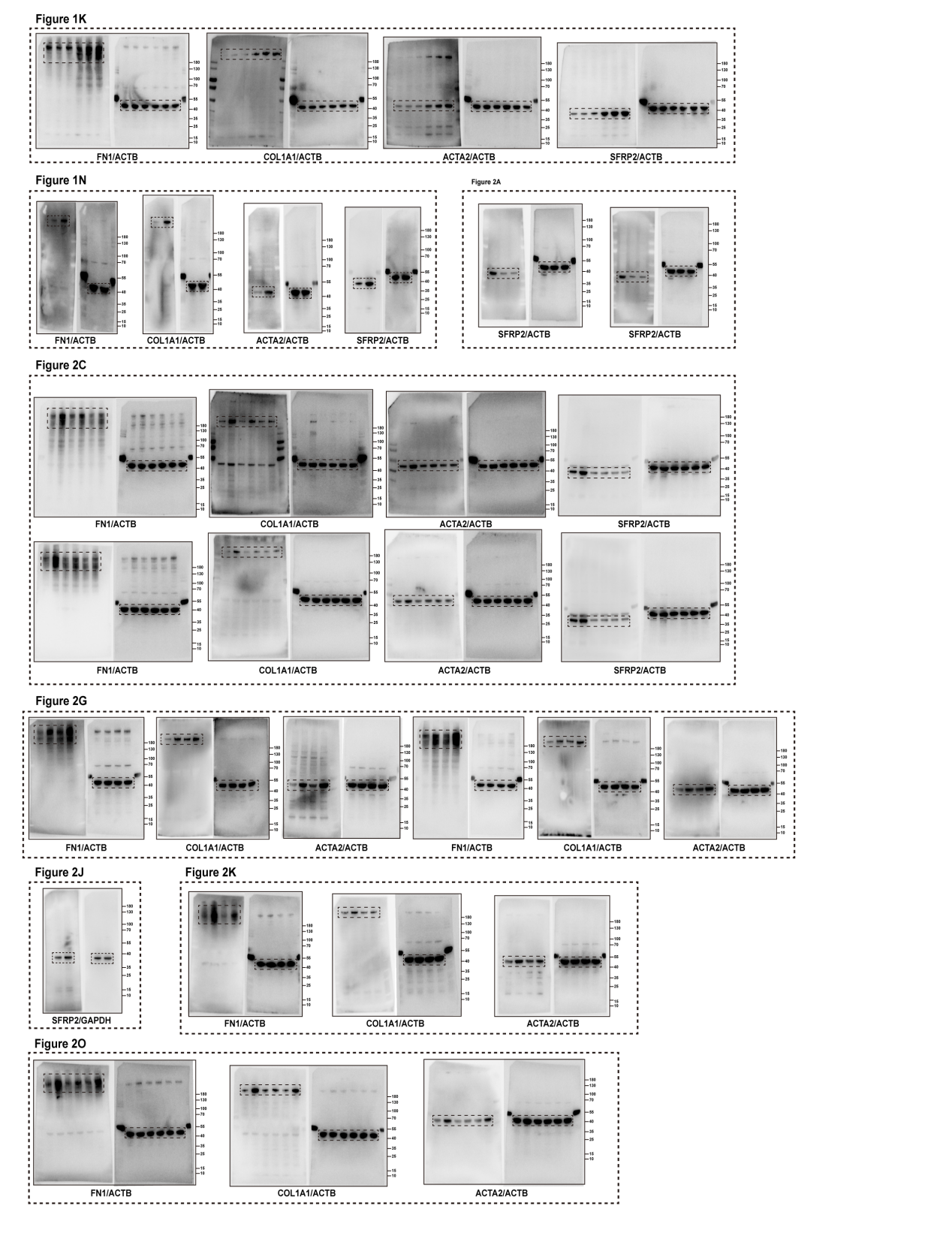


**Data S2**. Full-length and uncropped western blot images corresponding to Figure 1 and 2.


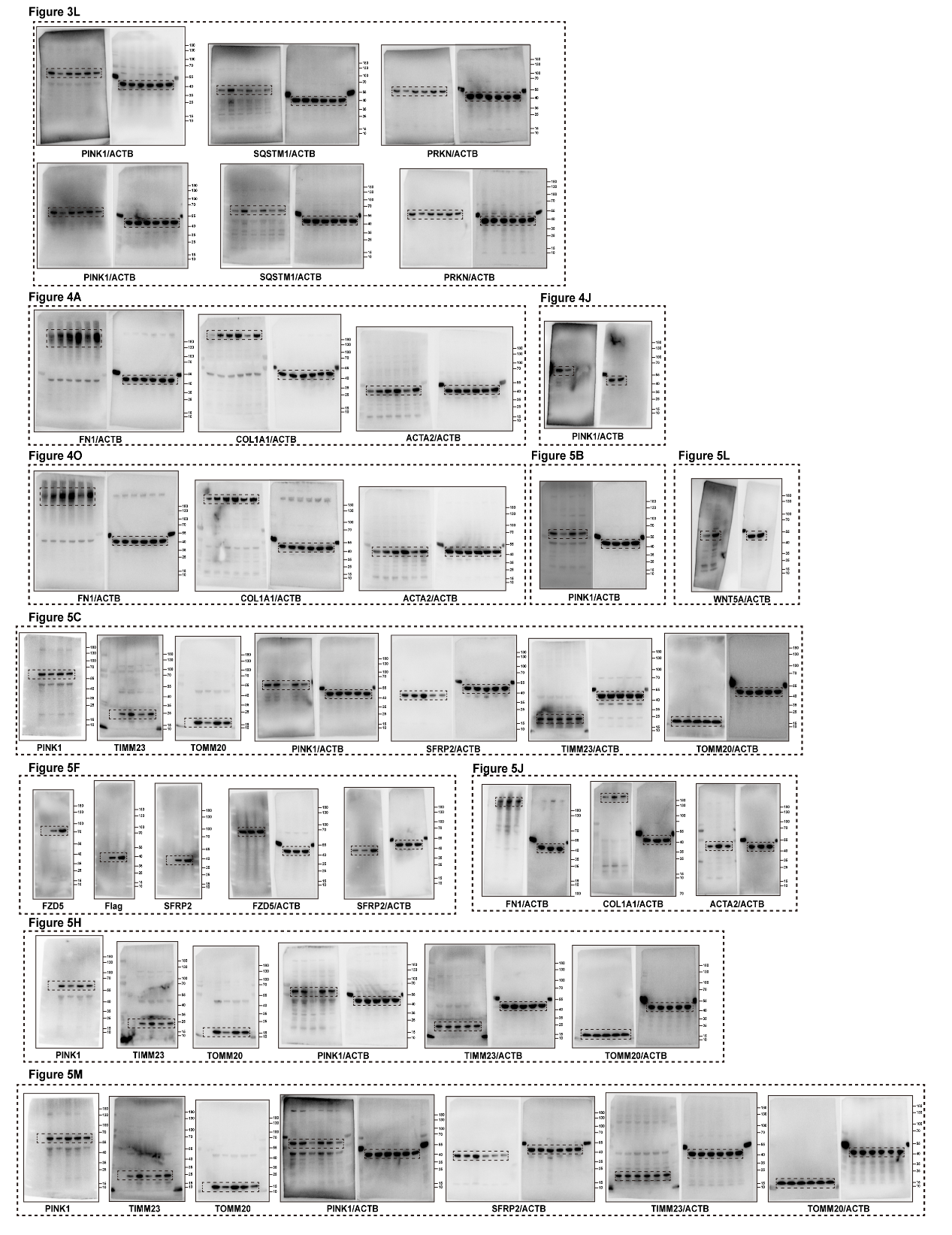


**Data S3**. Full-length and uncropped western blot images corresponding to Figure 3, Figure 4 and Figure 5.


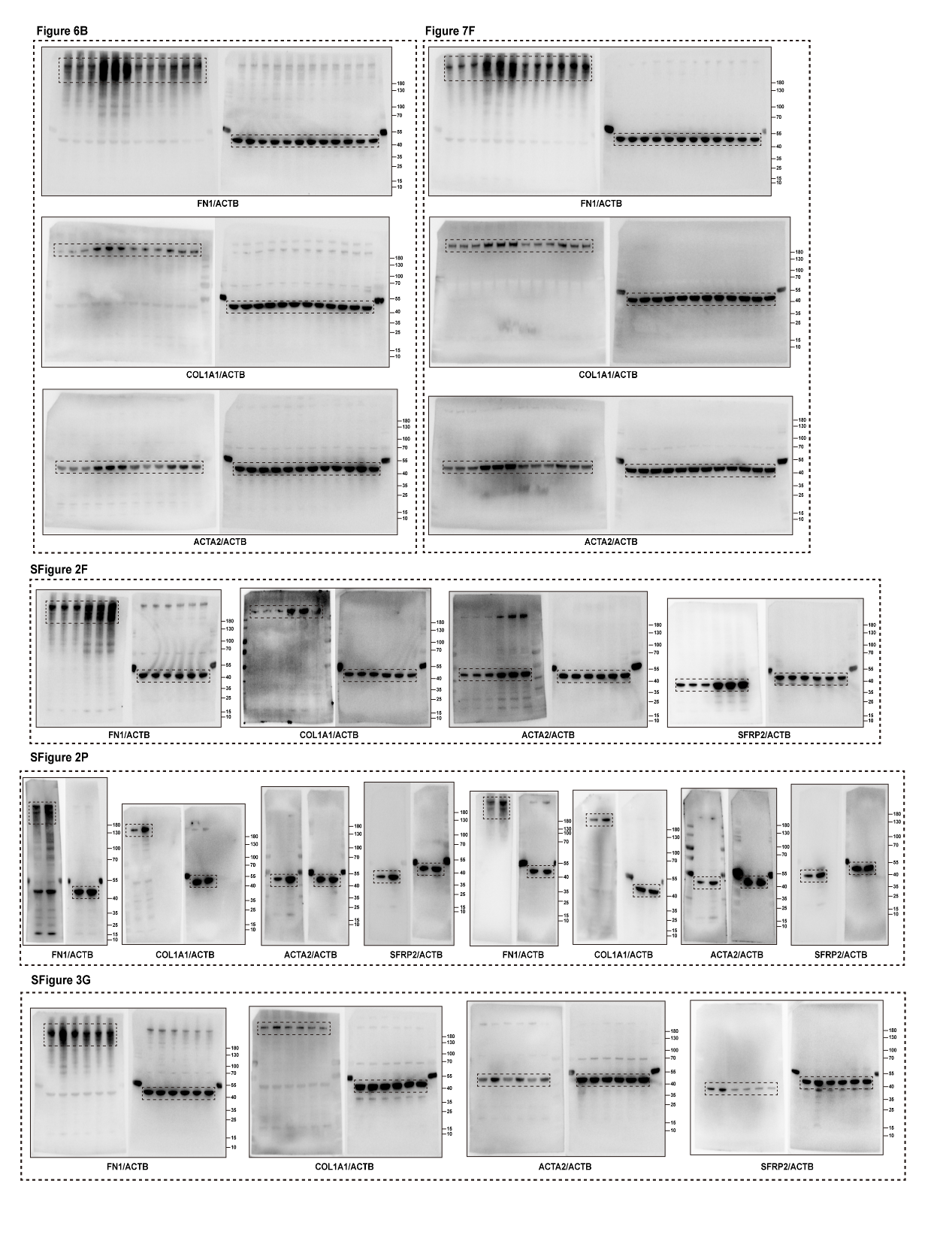


**Data S4**. Full-length and uncropped western blot images corresponding to Figure 6, Figure 7, Figure S2 and Figure S3.


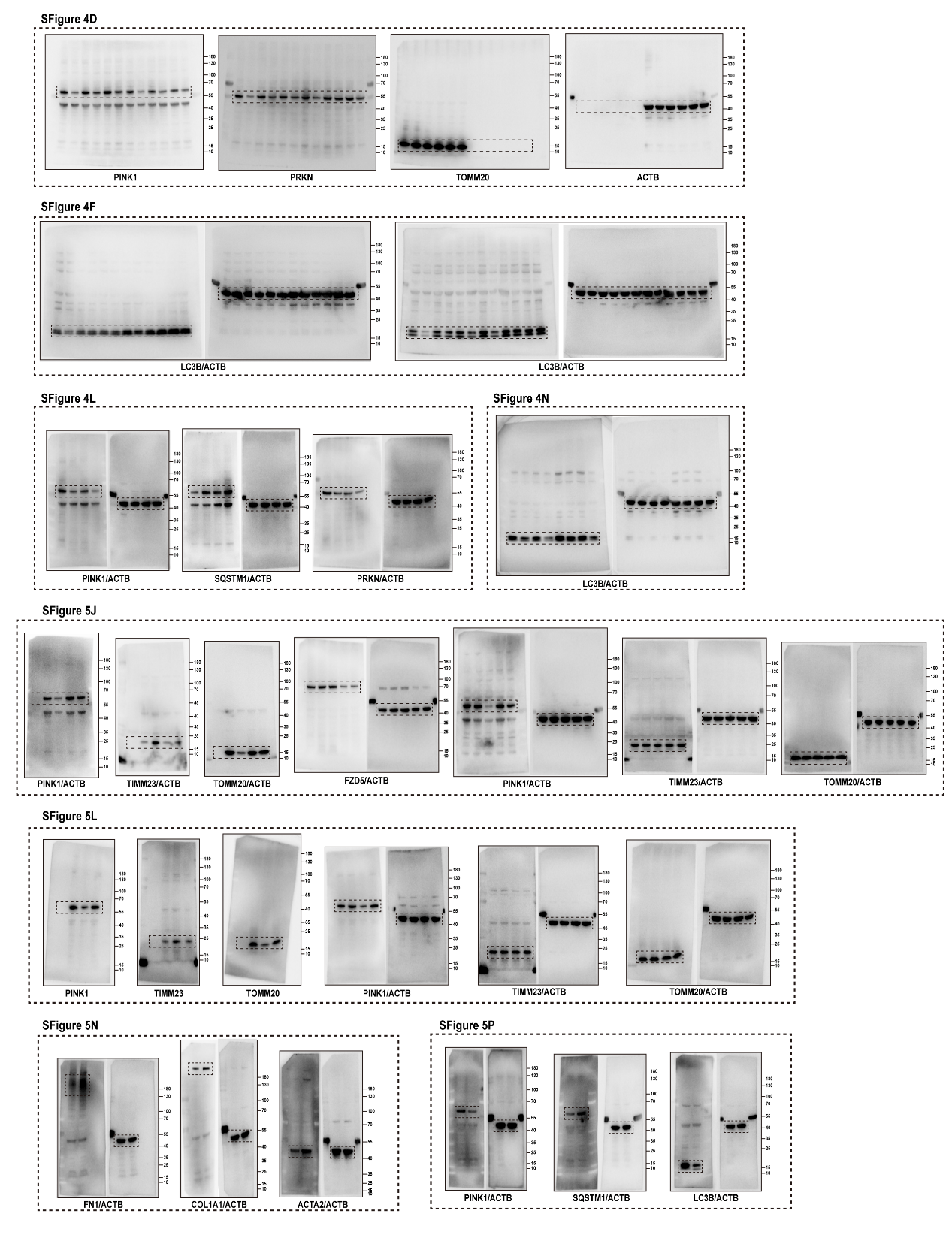


**Data S5**. Full-length and uncropped western blot images corresponding to Figure S4 and Figure S5.


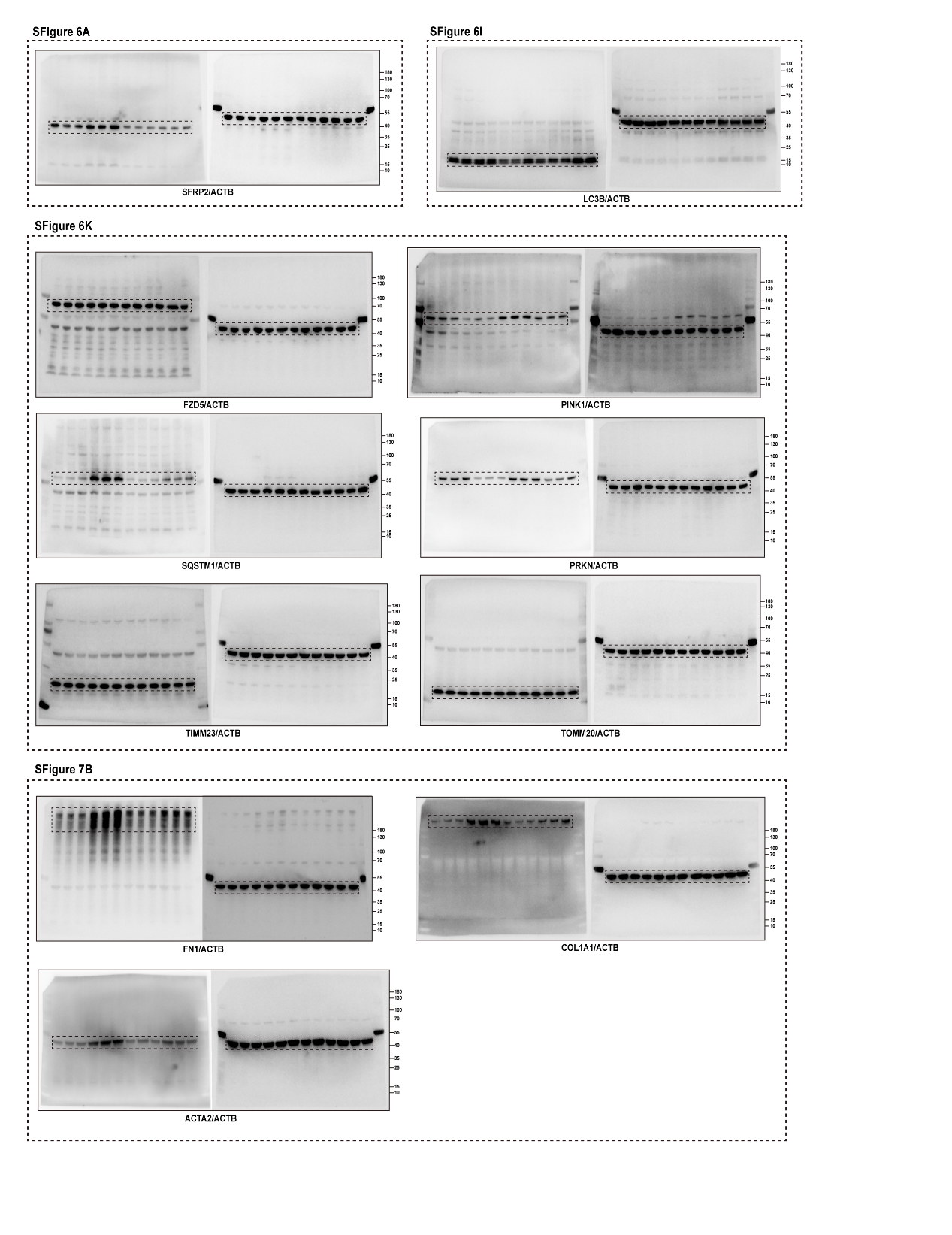


**Data S6**. Full-length and uncropped western blot images corresponding to Figure S6 and Figure S7.
